# Evolution of full-length genomes of HBV quasispecies in sera of patients with a coexistence of HBsAg and anti-HBs antibodies

**DOI:** 10.1038/s41598-017-00694-8

**Published:** 2017-04-06

**Authors:** Tai-Cheng Zhou, Xiao Li, Long Li, Xiao-Fei Li, Liang Zhang, Jia Wei

**Affiliations:** 1 Central lab, Liver disease research center, the second people’s hospital of Yunnan Province, Kunming, Yunnan Province China; 2 Clinical laboratory, the third people’s hospital of Kunming City, Kunming, Yunnan Province China

## Abstract

Although the evolutionary changes of viral quasispecies are correlated to the pathological status of a disease, little is known in the coexistence of hepatitis B surface antigen (HBsAg) and antibodies to these antigens (anti-HBs). To examine evolutionary changes in hepatitis B virus (HBV) and their relationship to the coexistence of HBsAg and anti-HBs antibodies, HBV genomes in patients with a coexistence of HBsAg and anti-HBs antibodies (experimental group) and HBsAg positive without anti-HBs (control group) were assessed. Our results showed that quasispecies diversity was significantly higher in the experimental group for large HBsAg (LHBsAg), middle HBsAg (MHBsAg), and HBsAg genes. LHBsAg harbored dN/dS values eight times higher in the experimental group; however, the mean dN/dS ratios in genes HbxAg, Pol and PreC/C of the experimental patients had an opposite trend. Phylogenetic trees in the experimental group were more complex than the control group. More positive selection sites, mutations and deletions were observed in the experimental group in specific regions. Furthermore, several amino acid variants in epitopes were potentially associated with the immune evasion. In conclusion, cumulative evolutionary changes in HBV genome that facilitate immune evasion provide insights into the genetic mechanism of a coexistence of HBsAg and anti-HBs antibodies.

## Introduction

Hepatitis B virus (HBV) infection is a serious global health problem, which can cause a series of liver diseases (including chronic hepatitis B (CHB), cirrhosis, hepatic failure, and hepato-cellular carcinoma (HCC))^1^. According to the World Health Organization, more than 2 billion people worldwide have been exposed to HBV^2^, with an annual death toll of 650,000 from the associated diseases^3^.

It is well-known in the medical field that antibodies to hepatitis B surface antigen (HBsAg), anti-HBs, are capable of neutralizing HBsAg and clearance of HBV, which is characterized by the emergence of anti-HBs and the disappearance of HBsAg from peripheral blood^4^. Hence it is a conundrum when both HBsAg and anti-HBs are present in same serological profile^5^. However, incidences of coexisting of HBsAg and anti-HBs among HBsAg positive patients has been increasingly reported, which is nearly 5% in China^5^, 21% in Japan^6^, 36% in Netherlands^7^, 2.8% in France^8^, 2.9% in South Korea^9^, varies from 2.8% to 36%^5–10^ and progressively increases with patient age from 40 to 70 years old^11^.

The molecular mechanism underlying the emergence of coexisting HBsAg and anti-HBs remains unclear. Several studies have been attributed to the selection of immune escape mutations in HBV genome, especially the determinant region (amino acids 124–147) of HBsAg, from one or a few viral strains^4, 8, 12–14^. Viral replication and the host immune response are two vital processes that interplay during HBV infection. The compact HBV genome contains four overlapping regions: preS/S completely overlaps with polymerase region, and HBxAg and HBcAg each partly overlap with polymerase region^15, 16^. These regions encode 7 proteins: DNA polymerase (Pol), three envelope proteins (LHBsAg, MHBsAg and HBsAg), core protein (HBcAg), X protein (HBx), and e protein (HBeAg). LHBsAg, MHBsAg, and HBsAg are structural proteins, while HBx regulates viral DNA replication and interferes with the host cell^17^. There are three types of Pol acting on DNA replication, and HBeAg may contribute to T cell immunological tolerance^18^.

Owing to a high replication rate and a lack of proof-reading capacity during reverse transcription, HBV exist as quasispecies^19^. Quasispecies is a population of closely related, but genetically distinct variants capable surviving living in a mutagenic environment^19^. The spectrum of mutants possess differing levels of fitness in a range of environments^20^. Thus, the serological profiles of HBV patients may be affected by competition among viral variants within quasispecies (differences in replicative efficiency)^19^ and/or host immune reactivity^21^.

In addition, because of amplification and sequencing problems, previous studies that compared HBV mutants between patients with the coexistence of HBsAg and anti-HBs antibodies and controls (patients who were HBsAg positive but anti-HBs negative) focused only on individual parts of the genome (preS, HBsAg and RT) which is associated with viral replication and immunoreaction^12, 22–24^. However, the viability and function of viruses are the result of interplay between all genes. Moreover, mutations in genes encoding HBxAg, HBcAg, and precore have been emphasized in HBV-associated diseases^25^. Thus the possible importance of multiple and concomitant mutations in these regions may be missed. Since compact HBV genomes contain overlapping genes, a mutation may impact functions of multiple genes^26^. Therefore, it is unreasonable to research coexistence of HBsAg and anti-HBs antibodies from the perspective of a partial HBV genome.

In light of coexisting of HBsAg and anti-HBs antibodies, it would be interesting to measure genome variability of HBV quasispecies in patients with this coexistence. Hence, this study aims to investigate the characteristics of HBV full-length genomes quasispecies in patients to elucidate the evolution of HBV quasispecies in patients with coexistence of HBsAg and anti-HBs.

## Results

### Background profiles of study subjects

Patients with a coexistence of HBsAg and anti-HBs antibodies (n = 6) and patients who were HBsAg positive only (n = 6) had similar characteristics and metabolite profiles: mean age, alanine aminotransferase (ALT) level, aspartate transaminase (AST) level, total bilirubin (TB), direct bilirubin (DB), total protein (TP), total albumin (ALB), HBeAg seropositive, and the HBV genotype, during the study period (*p* > 0.05 for all, Table 1). This suggests similar disease profiles between the experimental group and control group. Six males were in the experimental group while 3 females and 3 males were in the control group. The HBV-DNA levels in the experimental group were significantly lower than in the control group (6.57 ± 0.59 vs 7.57 ± 0.84 log_10_IU/ml, HBV DNA level >10^3^, *p* = 0.038, Table 1), which is consistent with a previous study^27^. In total, 178 full-length genomes of HBV were analyzed for viral diversity, phylogenetic divergence, selection pressure, and positive selection.

**Table 1:** Demographic and clinical features of studied patients.

Characteristic	Experimental group (n = 6)	Control group (n = 6)	*P* value
Age (mean ± SD) (yr)	40.16 ± 14.22	29.33 ± 8.68	0.140
Gender (no. of males/no. of females)	6/0	3/3	—
No. of HBeAg(+)/sum	5/6	6/6	1.000
ALT (mean ± SD) (IU/ml)	161.67 ± 107.14	74.83 ± 43.19	0.100
HBV DNA (mean ± SD) (log_10_ IU/ml)	6.57 ± 0.59	7.57 ± 0.84	**0.038**
AST (mean ± SD) (IU/ml)	91.5 ± 68.23	39.67 ± 13.59	0.120
TB (mean ± SD) (umol/liter)	18.15 ± 8.72	13.05 ± 4.21	0.240
DB (mean ± SD) (umol/liter)	5.93 ± 3.39	3.97 ± 1.27	0.210
TP (mean ± SD) (mg/ml)	66.82 ± 7.88	72.83 ± 4.89	0.140
ALB (mean ± SD) (mg/ml)	41 ± 5	45.88 ± 4.32	0.100
HBsAb (mean ± SD) (mIU/ml)	31.6 ± 20.81	—	—
No. with HBV genotype (B, C, I)	1/4/1	1/4/1	1.000

### Complexity and diversity of HBV quasispecies

**Figure 1:**
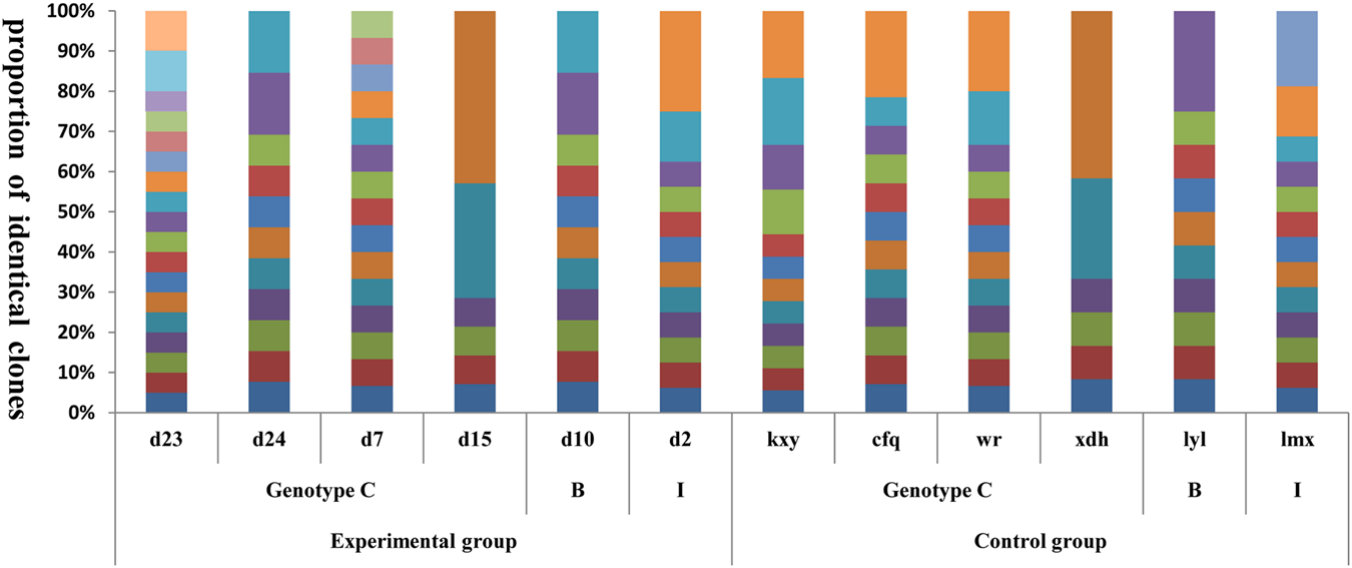
HBV full-length quasispecies in the experimental group and control group. The vertical bars indicate the proportion of viral quasispecies within each patient. Each color represents one kind of quasispecies. Y axis: proportion of identical clones; X axis: each patient.

Comparable quasispecies complexity and diversity were found among the full-length HBV genomes (Fig. 1) and individual genes in all patients for each group (Tables 2 and 3). Sn of nucleotides and amino acids for HBV full-length genome (at the nucleotide level only), genes encoding LHBsAg, MHBsAg, HBsAg, HBxAg, HBcAg, Pol, reverse transcriptase (RT), and core promoter (CP) region (at the nucleotide level only) were not significantly greater in the experimental group than the control group (Table 2). The QS diversity (d, dS, and dN) distribution was statistically significant among the experimental and control groups (Table 3). The experimental group had a significantly higher mean genetic distance (d) and Dn in the LHBsAg, MHBsAg, and HBsAg (*p* < 0.05) at the nucleotide and amino acid levels. All other genes were statistically similar among the groups.

**Table 2:** Quasispecies complexity (Sn) between the patients with coexisting HBsAg and anti-HBs and controls.

Region of variation (level)	Experimental group (mean ± SE) (n = 6)	Control group (mean ± SE) (n = 6)	*P* value
Full length (nt)	0.865 ± 0.0647	0.8334 ± 0.0445	0.695
PreS/S			
Large HBsAg (nt)	0.8488 ± 0.0615	0.8241 ± 0.057	0.774
Large HBsAg (aa)	0.7889 ± 0.0646	0.6929 ± 0.0593	0.300
Middle HBsAg (nt)	0.7819 ± 0.0562	0.7673 ± 0.0499	0.850
Middle HBsAg (aa)	0.7434 ± 0.051	0.5897 ± 0.0773	0.128
HBsAg (nt)	0.7484 ± 0.0548	0.6535 ± 0.0691	0.307
HBsAg (aa)	0.6756 ± 0.0646	0.517 ± 0.0942	0.195
X			
HBxAg (nt)	0.6766 ± 0.0683	0.6321 ± 0.0742	0.668
HBxAg (aa)	0.5322 ± 0.0871	0.5177 ± 0.0423	0.884
CP/C			
HBcAg (nt)	0.6154 ± 0.0724	0.6715 ± 0.0519	0.543
HBcAg (aa)	0.452 ± 0.0627	0.5362 ± 0.0904	0.462
Core promoter (nt)	0.5367 ± 0.0297	0.5113 ± 0.0443	0.645
procore (nt)	0.6758 ± 0.0555	0.6997 ± 0.065	0.785
P			
Polymerase (nt)	0.865 ± 0.0647	0.8055 ± 0.0521	0.490
Polymerase (aa)	0.8381 ± 0.0652	0.8052 ± 0.0405	0.677
RT region (nt)	0.7889 ± 0.0613	0.7562 ± 0.0496	0.687
RT region (aa)	0.7631 ± 0.0503	0.6989 ± 0.0601	0.432

**Table 3:** Quasispecies diversity (genetic distance, dS, and dN) between the patients with coexisting HBsAg and anti-HBs and controls.

Region of variation (level)	Experimental group	Control group	*P* value
	(mean ± SE) (n = 6)	(mean ± SE) (n = 6)	
Full length (nt)	0.0076 ± 0.0021	0.0037 ± 0.0005	0.122
PreS/S			
Large HBsAg (nt)	0.0088 ± 0.0021	0.003 ± 0.0004	0.04
Large HBsAg (aa)	0.0183 ± 0.0044	0.0057 ± 0.001	0.034
Middle HBsAg (nt)	0.0087 ± 0.0019	0.003 ± 0.0004	0.033
Middle HBsAg (aa)	0.0187 ± 0.0044	0.0061 ± 0.0014	0.036
HBsAg (nt)	0.009 ± 0.002	0.0039 ± 0.0012	0.05
HBsAg (aa)	0.0196 ± 0.0048	0.0069 ± 0.0016	0.045
X			
HBxAg (nt)	0.0068 ± 0.002	0.0051 ± 0.0011	0.47
HBxAg (aa)	0.0126 ± 0.0039	0.0095 ± 0.0013	0.481
C/CP			
HBcAg (nt)	0.009 ± 0.0032	0.0048 ± 0.0008	0.235
HBcAg (aa)	0.0152 ± 0.0065	0.0101 ± 0.0023	0.48
Core promoter (nt)	0.0069 ± 0.0028	0.0063 ± 0.0017	0.876
P			
Polymerase (nt)	0.0072 ± 0.0018	0.0034 ± 0.0004	0.091
Polymerase (aa)	0.0117 ± 0.0026	0.0063 ± 0.0008	0.097
RT region (nt)	0.0079 ± 0.002	0.0032 ± 0.0005	0.071
RT region (aa)	0.0114 ± 0.0025	0.0059 ± 0.0009	0.066
PreS/S			
Large HBsAg (dS)	0.0089 ± 0.0021	0.0042 ± 0.001	0.068
Large HBsAg (dN)	0.0085 ± 0.0021	0.0025 ± 0.0004	0.037
Middle HBsAg (dS)	0.0073 ± 0.0014	0.0038 ± 0.0007	0.05
Middle HBsAg (dN)	0.0088 ± 0.0022	0.0027 ± 0.0006	0.043
HBsAg (dS)	0.0085 ± 0.003	0.0063 ± 0.0034	0.633
HBsAg (dN)	0.009 ± 0.0023	0.0031 ± 0.0007	0.049
X			
HBxAg (dS)	0.0099 ± 0.0031	0.0064 ± 0.0023	0.39
HBxAg (dN)	0.0058 ± 0.0018	0.0044 ± 0.0007	0.512
C			
HBcAg (dS)	0.0149 ± 0.0052	0.0051 ± 0.0019	0.127
HBcAg (dN)	0.007 ± 0.0031	0.0045 ± 0.001	0.469
P			
Polymerase (dS)	0.0129 ± 0.0037	0.0052 ± 0.0009	0.094
Polymerase (dN)	0.0053 ± 0.0012	0.0028 ± 0.0004	0.093
RT region (dS)	0.0163 ± 0.005	0.0051 ± 0.0016	0.079
RT region (dN)	0.0052 ± 0.0012	0.0026 ± 0.0004	0.08

**Figure 2:**
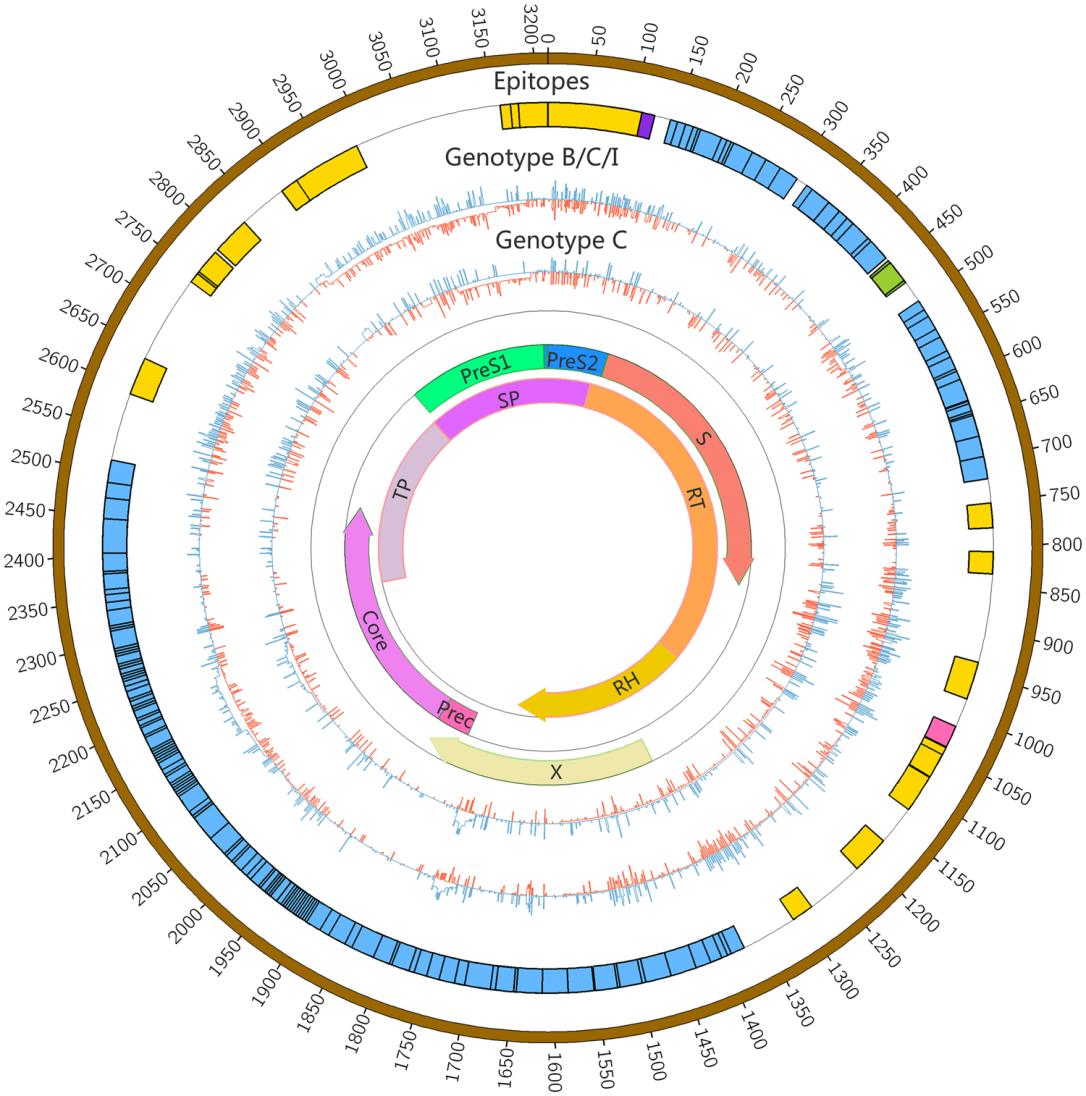
HBV nucleic acid (nt 1 to 3215) complexities for the experimental group and control group. The colored bars indicate the complexity of each nucleotide for the experimental group (red lines) and control group (blue lines) for the full-length HBV genome. The insertions were discarded. Epitope distribution is as follows: B cell epitopes (purple), HLA I T cell epitopes (yellow), HLA II T cell epitopes (carnation), overlap of two types of epitopes (green), overlap of three types of epitopes (blue).

The complexity of nucleotide positions varied among the three HBV genome genotypes (Fig. 2). Among the three genotypes, the control groups typically had fewer mutations than the experimental groups, while an exceptional number of mutations were found in the genes encoding LHBsAg, MHBsAg, and HBsAg in genotype C (*p* < 0.001, Supplementary Table 1), which located in HLA I T cell epitopes or multiple types epitopes (Fig. 2 and Supplementary Table 2).

### Phylogenetic analysis

**Figure 3:**
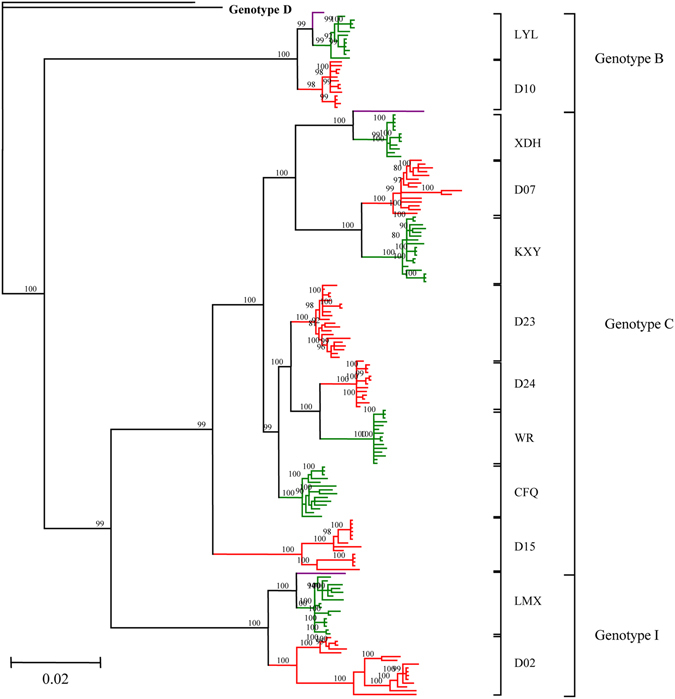
Phylogenetic trees were constructed for sequences from both the experimental group (red) and the control group (green) with a Bayesian method. Two complete genomes of HBV genotype D (AB090269 and AB104710) served as the outgroup sequences and three genomes (nucleotide sequences D00330 for Genotype B, AB033556 for Genotype C and FJ023667 for Genotype I) served as experimental sequences. Bootstrap values >90% are shown.

Two complete HBV genotype D genomes (AB090269 and AB104710) served as the outgroup sequences for the other 3 genotypes B, C, and I (GU815660, FJ562300, and GU357844, respectively) in the Bayesian phylogenetic analysis (Fig. 3). Generally, all sequences clustered together according to genotypes. Sequences from the same patient clustered together as expected, which suggests that they were homologous. The branch lengths of trees from the experimental group were consistently longer than those of the control group, indicating a higher rate of mutation accumulation in the HBV experimental group genotypes, which concurs with the higher quasispecies diversity in the experimental group.

### Selective pressure

**Figure 4:**
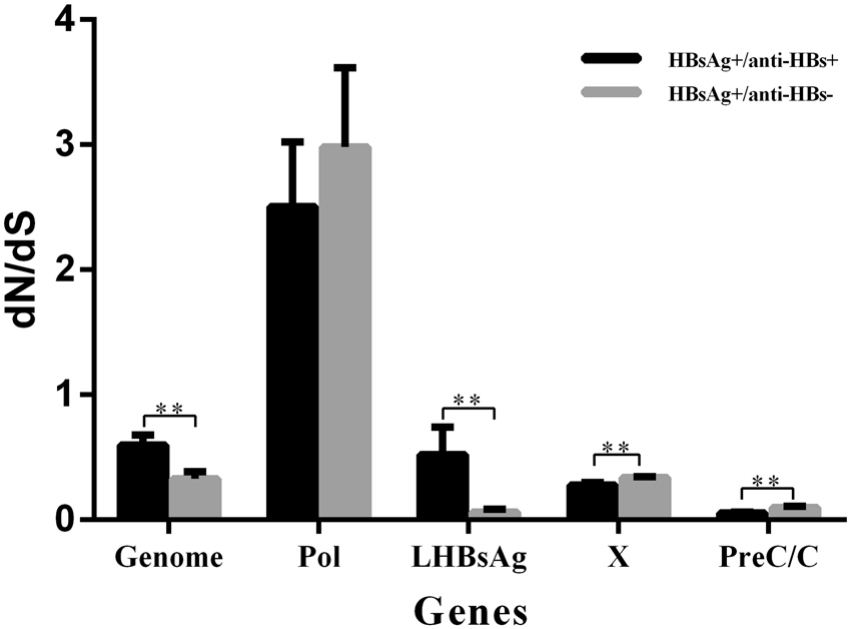
dN/dS ratios for HBV genes LHBsAg, Pol, HBxAg, and PreC/C.

**Figure 5:**
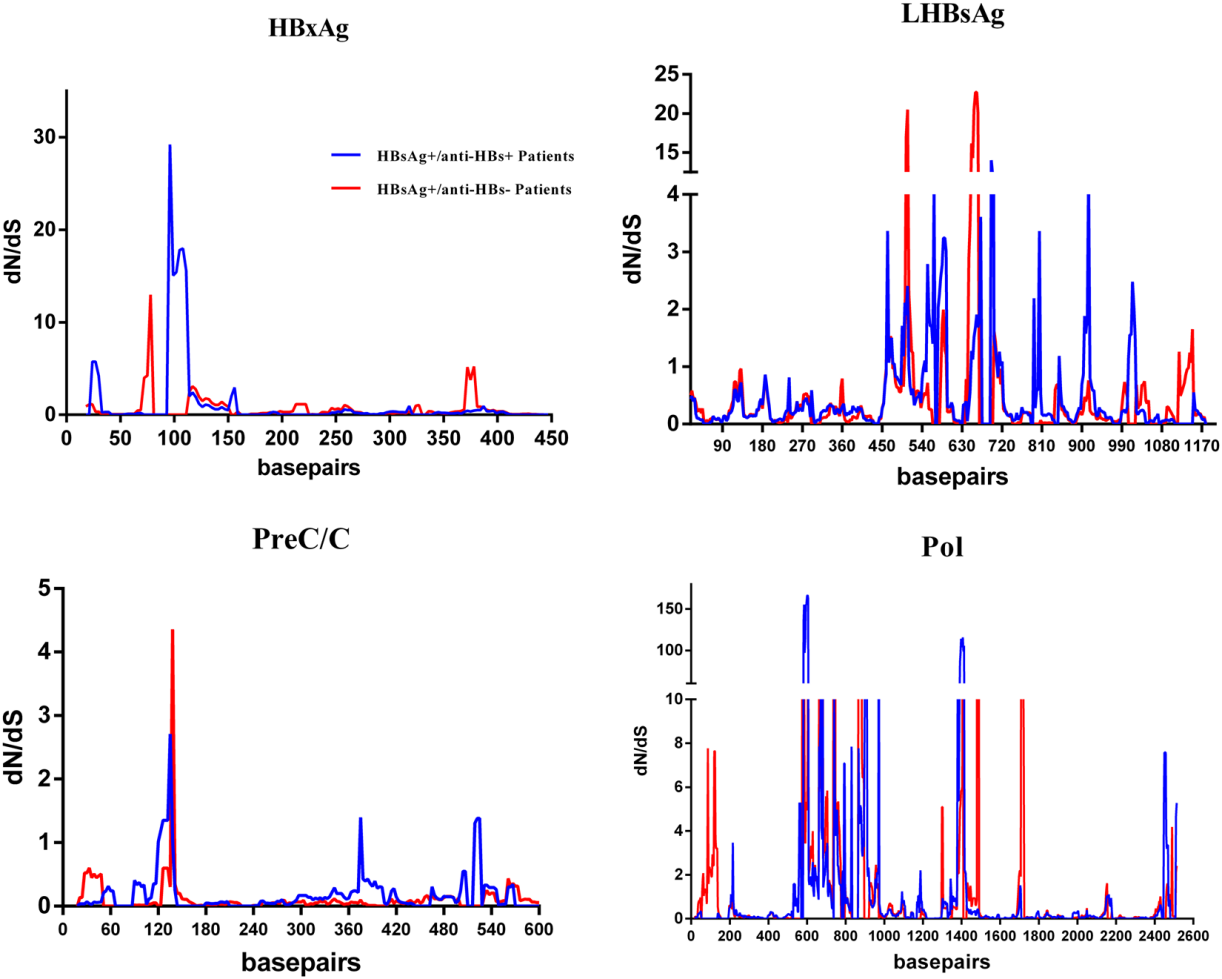
dN/dS in four genes (LHBsAg, Pol, HbxAg, and PreC/C) were verified by a sliding windowanalysis using the Nei-Gojobori distance estimation method (window length, 15 nt; window step, 3 nt).

The dN/dS ratio was used to measure the selective pressure across individual lineages established with 65 experimental group sequences and 68 control group sequences (Supplementary Figure 1). We utilized the free ratio model (M1) in PAML4 to calculate the dN/dS ratio on the 4 protein-coding genes individually (Pol, LHBsAg, HBxAg and PreC/C), and concatenated. Only dN/dS values associated with terminal branches were used in the subsequent analyses. Because outliers with larger dN/dS may generate deviations in evaluating the overall selection pressure, our dataset was filtered to remove values outside of the standard error, yielding 57 experimental group values and 60 control group values for the concatenated genes dataset, and 52 vs 55 values for PreC/C, 51 vs 52 for HBxAg, 51 vs 55 for Pol, and 46 vs 51 for LHBsAg. While the dN/dS ratio did not indicate directional selection, the ratios were statistically higher in the experimental group (0.5996 ± 0.0798, 45.3% larger, *p* = 0.04). The LHBsAg gene harbored dN/dS values eight times higher in the experimental group (0.5218 ± 0.2184 vs 0.0612 ± 0.0247, *p* = 0.03) (Supplementary Table 3 and Fig. 4). However, the mean dN/dS ratios in genes HBxAg or PreC/C were significantly lower in the control group 0.3398 ± 0.0061 or 0.0981 ± 0.01 respectively (*p* = 0.001). Both groups had dN/dS >1 in the Pol gene without a significant difference (*p* = 0.56). The variability of dN/dS in these four genes was further detailed using a sliding window analysis (Fig. 5).

### Positive selection

A codon-based molecular evolution model was used to identify positive selection in four genes (Pol, LHBsAg, HBxAg, and PreC/C). In both groups, Pol, LHBsAg and HBxAg were all under positive selection using the M8 model (Supplementary Table 4). Fifteen positively selected sites were identified in these genes using the Bayes empirical method (LHBsAg, 1; HBxAg, 5; Pol, 9) (Table 4 and Supplementary Table 4). The numbers of clones with 10 positive selection sites were significantly different (*p* < 0.01) between the experimental group and the control group. In particular, significantly more positive selection sites (*p* < 0.05) were found with more clones in the experimental group (7/10) than in the control group (3/10). When the positive selection sites were mapped to previously identified epitopes, 6 of 10 positive selection sites were found in one or more epitopes. Interestingly, positively selected codon positions 300 of LHBsAg and 480 of Pol are from the same HBV genome site, however, compared with the control patients, the number of clones with the LHBsAg 300 mutation are significantly higher in the experimental group (*p* = 0.037) while the opposite trend is seen in Pol 480 mutation (*p* = 0.005) (Table 4).

**Table 4:** Codons in the HBV genes that were under positive selection.

Gene	Codon	Wild type amino acid (B, C, I genotype)	Posterior probability	Omega (±s.e.m.)	Subjects and clones at each site^d^		*p* value^e^
					Experimental group 91(6)	Control group 87(6)	
LHBsAg	**300**	**T**, **I**, **I** ^**a**,**b**,**c**^	0.936	1.633 ± 0.479	29(3)	15(1)	**0.037**
**HBxAg**	36	A, P, D	0.999	3.753 ± 0.823	62(5)	59(4)	1.000
	**44**	**V**, **A**, **A** ^**c**^	0.91	3.430 ± 1.074	14(2)	12(1)	0.930
	**127**	**V**, **I**, **I** ^**a**,**b**^	0.979	3.688 ± 0.902	45(3)	8(2)	**1.149E-08**
	**130**	**K** ^**a**,**b**^	1	3.755 ± 0.820	78(5)	45(5)	**2.100E-06**
	**131**	**V** ^**a**,**b**^	1	3.756 ± 0.819	78(5)	30(4)	**7.854E-12**
**Pol**	136	Y, H, Q	0.908	2.381 ± 0.544	33(3)	14(1)	**0.004**
	237	Q, S, S	0.925	2.411 ± 0.509	30(3)	14(1)	**0.015**
	251	P, T, T	0.927	2.414 ± 0.505	25(2)	31(2)	0.312
	480	N, D, D	0.914	2.392 ± 0.531	4(1)	15(1)	**0.005**
	613	Q, L, Q	0.902	2.370 ± 0.563	49(3)	15(2)	**8.179E-07**
	615	I, L, I	0.936	2.429 ± 0.479	14(1)	29(3)	**0.009**
	713	Q, R, Q	0.933	2.424 ± 0.489	36(3)	30(2)	0.585
	**803**	**R**, **H**, **H** ^**a**^	0.992	2.525 ± 0.278	29(4)	49(4)	**0.002**
	841	R, K, R	1	2.538 ± 0.235	53(4)	39(5)	0.101

^a^HLA I T cell epitopes, ^b^HLA II T cell epitopes, ^c^B cell epitopes.

^d^The number of clones with positive selection (the number of affected patients).

^e^The number of clones with positive selection in HBsAg+/anti-HBs + group vs HBsAg+/anti-HBs- group, chi-square testing.

### Hot spot mutations

None of the primary lamivudine-, telbivudine-, entecavir-, adefovir-, or tenofovir- related resistance mutations were found in the RT region among 178 clones, but lamivudine compensatory mutations were found in 10 clones from two patients at rtA181T(d02,1/16 clones), rtM204V(d02,1/16 clones), and rtN236T(d10,8/13 clones).

**Figure 6:**
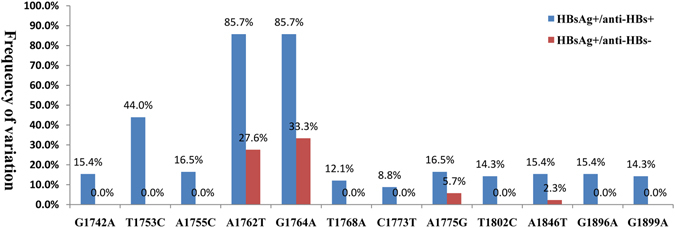
Frequency of nucleotide point mutations in the core promoter and precore regions (1742–1900 bp), p < 0.05.

For the HBV genomes, the experimental group accumulated 1154 mutations while the control group had 812 according to respective references (Supplementary Table 5). There were more than 50 mutations in each patient, of which patient d02 had the most mutations (214). Significant aa substitution diversity was observed within the “a” determinant of HBV between the experimental group and the control group (4.76 vs. 0.91, for substitution per 100 aa, p < 0.01), which is consistent with previous research^13, 14^ (Supplementary Table 6). The frequency of potential influential variations associated with the clinical spectrum at the core promoter and precore regions, compared to the reference sequences in HBV treatment-naive patients, is summarized in Fig. 6 and Supplementary Table 7. We identified 15 mutations in this region between the experimental group and the control group. The experimental group had a higher frequency of mutations at G1742A (15.4%), T1753C (44.0%), A1755C (16.5%), A1762T (85.7%), G1764A (85.7%), T1768A (12.1%), C1773T (8.8%), A1775G (16.5%), T1802C (14.3%), A1846T (15.4%), G1896A (15.4%), and G1899A (14.3%) than the control group (*p* < 0.01). In addition, the number of basal core promoter (BCP) A1762T/G1764A double mutations in the experimental group patients (78/91 clones) was significantly higher than in control patients (24/87 clones) (*p* < 0.01).

Because few patients had HBV genotype B and I, only patients with genotype C were used for amino acid variation analyses. Eighty-nine sites had statistical differences in frequency of variationbetween the experimental group and the control group: HBcAg(10), HBxAg(16), LHBsAg(44), and RT(29) (p < 0.05, Supplementary Table 8). The experimental group had a higher mutation frequency in LHBsAg (45.1% [28/62 clones]) than the control group (25.4% [15/59 clones]) (*p* < 0.05) at I300. More than half (74.2% [46/62]) of the clones in the experimental group had a mutation at N177, while only 30.5% (18/59 clones) were mutated at this site in the control group. The distribution of RT amino acid mutations (S7, P271, and S280) had the highest frequency of variation in each group of patients. The high frequency of variation (100%) in HBxAg amino acids in the control group were S29, N48, and Y94, but G36, L42, and Y132 were the most common in the experimental group.

### Deletion, insertion, and stop codon mutations

DNA insertions were only observed in the HBxAg gene in the experimental group (Table 5 and Supplementary Table 9). Nonsense mutations were observed more frequently in the experimental group (35/91) than in the control (4/87); however, deletions displayed the opposite trend ([33/91] vs [54/87]). In experimental group patients, nonsense mutations were mostly found in the LHBsAg and PreC/C genes. Likewise, deletions were also found in the LHBsAg gene in the experimental group, yet most deletions in the control group were found mainly in the in HBxAg and PreC/C genes.

**Table 5:** The number of deletion, insertion, and stop codon mutations of HBV quasispecies observed.

Genes	Mutations	Control group (72)	Experimental group (91)	*P* value
Genome	Del	54	33	—
	Ins	3	0	—
	Stop codon mutations	4	35	*p* < 0.05
	Sum	61	68	—
LHBsAg	Del	12	30	*p* < 0.05
	Stopcodon mutations	2	16	*p* < 0.05
	Sum	14	46	*p* < 0.05
HBxAg	Del	23	2	*p* < 0.05
	Ins	3	0	—
	Sum	26	2	*p* < 0.05
PreC/C	Del	19	0	*p* < 0.05
	Stop codon mutations	0	14	*p* < 0.05
	Sum	19	14	—
Pol	Del	13	31	—
	Stop codon mutations	2	5	—
	Sum	15	36	*p* < 0.05

## Discussion

The coexistence of HBsAg and anti-HBs antibodies is a special serological profile which has been increasingly reported^11, 12, 27^. In this study, the full-length genomes of HBV quasispecies in patients with a coexistence of HBsAg and anti-HBs antibodies were examined in Southwest China. Demographic and clinical features did not have a statistical difference between the control and the experimental group patients, with the exception of increased HBV DNA levels. Compared with the control patients, the patients with positive anti-HBs antibodies had lower HBV DNA levels (*p* = 0.038), which is consistent with a previous large sample study, which hints that anti-HBs antibodies can partially neutralize HBsAg and clear circulating HBV particles^27^.

Recently, many studies argue that selection for immune escape mutations explains the coexistence of HBsAg and anti-HBs antibodies^4, 28, 29^. Mutations in the preS/S gene are hot spots for studies^28, 30^, yet results from the different studies are not wholly comparable. To combat this problem, we analyzed the full-length genomes of HBV quasispecies in patients with coexisting HBsAg and anti-HBs antibodies. Analysis of 178 full-length genome sequences from 12 HBV carriers showed that the viral diversity was significantly greater in the experimental group for the LHBsAg, MHBsAg, and HBsAg genes (Table 3, *p* < 0.05). This is consistent with the higher nucleotide Sn (frequency of mutated positions) and Dn for these genes in the experimental group (*p* < 0.001). The phylogenetic trees were complex in experimental group patients, and relatively simple for control patients, which may be attributed the diversity of HBV genes (LHBsAg, MHBsAg, and HBsAg).

The ratio of dN/dS is used to measure the strength of selection acting on protein-coding genes^31^. To estimate the immune selection pressure on HBV genome, we calculated the ratio of dN/dS on protein-coding genes (LHBsAg, HBxAg, PreC/C, and Pol). The dN/dS ratios of two genes (HBxAg and PreC/C) <1 in the experimental group was significantly lower than that of the control group (*p* < 0.01), indicating relatively strong negative selection in the experimental group compared to the control group. This is in agreement with a previous study that patients with a coexistence of HBsAg and anti-HBs are under strong humoral immune-pressure^12^. Therefore, viral strains with highly mutated HBsAg may be enriched by this selection pressure thereby reducing the exposure of HBsAg immunogenic surface^32^. The mean dN/dS ratio of LHBsAg in the experimental group were 8 times greater than the control groups, which when combined with a higher Dn value, suggests that the experimental group accumulated more amino acid variants in LHBsAg. It is important to note that mutations in the LHBsAg gene may imperil efficient viral replication^33^, which could also account for the relatively lower serum HBV-DNA levels observed in the experimental group.

Positive selection is the process by which amino acid sites are preferentially selected to enhance viral fitness^34^. In a plausible response to immunological pressure, 15 positively selected amino acid sites were detected in three genes (LHBsAg, HBxAg, and Pol). Compared to the control group, an increased frequency (7/10) of positively selected sites was identified in the experimental group, more than half of which are located in an epitope site (4/7). This suggests that positively selected amino acid sites may change the characteristics of epitopes to escape immune detection. Interestingly, codon position 300 of LHBsAg (126aa of HBsAg at “a” determinant) whose first and second bases overlap the second and third bases of codon position 480 of Pol, is closely related to immune evasion in many studies^27, 35, 36^. In our study, both of these genes have undergone positive selection. Considering the functional importance of LHBsAg codon 300, positive selection on codon 480 of Pol may attribute to the hitchhiking effect of a strongly selected substitution (codon 300 of LHBsAg)^37, 38^. However, further research needs to be conducted to test these potential functional changes in LHBsAg and Pol.

We also noted related mutations in the HBV genome which are rarely reported in patients with the coexistence of HBsAg and anti-HBs antibodies. No NA resistance-related primary mutations were found, which is in accordance with the treatment-naive HBV-infected samples. The pathogenesis of coexisting HBsAg and anti-HBs antibodies remains unclear; however increasing evidence suggests that the coexistence of HBsAg and anti-HBs antibodies is associated with the severity of liver disease and the active replication/reactivation of virus^6, 8, 39^. Mutations of the core promoter (CP) and precore are prevalent in HBV chronic infection^40^ and the severity of liver disease associated with core promoter (T1753C, A1762T, G1764A, T1768A and A1846T/C) and precore (G1896A and G1899A) mutations has been widely discussed^41–44^. BCP A1762T/G1764A are dual mutations which enhance viral genome replication^41, 42^, and may partially resist neutralization of anti-HBs. In addition to the above mutations, the frequency of mutations (G1742A, A1755C, A1775G, C1773T and T1802C) in the experimental group was significantly higher than in the control group (*p* < 0.05). In addition, the coexistence of HBsAg and anti-HBs antibodies with HBV-related liver disease may be attributed to a high mutation frequency in the core promoter and precore regions; however, further study is needed to reinforce this conclusion. Reports show that HBcAg and HBxAg peptides are more conserved than surface and polymerase peptides in HBV^45^. In our study, we also found that the LHBsAg and RT genes had more mutations than HBxAg and HBcAg in genotype C. However, with the exclusion of HBxAg, most mutations were found in the experimental group and located in the HLA I, HLA II, or B epitopes, which suggests that accumulating mutations may provide an opportunity to escape from host immune pressure.

Deletions, insertions, and nonsense mutations in HBV are associated with viral hepatitis progression^46^. Several nucleotide deletions and nonsense mutations in LHBsAg result in truncations of LHBsAg (preS1/S2) or HBsAg, potentially altering HBV immunogenicity^47^. Interestingly, these mutations were more frequent and complex in the experimental group than in the control group (Table 5). The coexistence of wild-type HBV and strains with deletions in the core gene has been shown to enhance viral replication^12^. In our study, deletions in PreC/C were only detected in the control group which is consistent with their significantly higher viral load. A precore stop codon (PSC) mutation (G1896A), resulted in reduced or a lack of HBeAg production^48^, was only detected in the experimental group.

Compared to next-generation sequencing (NGS), clone-based sequencing can cover the full length HBV genome and is still prevalent for studying heterogeneous viral QS^46^. The present study is the first to describe the characteristics of full-length genome QS patients with a coexistence of HBsAg and anti-HBs antibodies; possibly planting a seed for future studies that can include more patients and clones to capture a greater proportion of the variation in the HBsAg population.

## Conclusion

Understanding the evolution of HBV quasispecies can increase our understanding of HBV pathogenesis^49^. In our study, we have shown that HBV quasispecies viral diversity was increased in the experimental group, which was associated with increased mutations in the core promoter (CP) and precore, dN/dS of LHBsAg, deletions of LHBsAg, amino acid variations of LHBsAg and RT, followed by a reduction in HBV-DNA levels and positively selected amino acids. These findings suggest that patients with a coexistence of HBsAg and anti-HBs antibodies may have dynamic shifts in the serum HBV quasispecies. We can speculate that increased HBV genetic diversity may lead to altered T and B-cell epitopes, stimulation of innate immune responses, and possible alterations in HBV physiology. Further insights into the pathophysiology of this coexistence will rely on investigating these potential mechanisms.

## Materials and Methods

### Patients

Twelve treatment-naive HBV-infected inpatients from the Second People’s Hospital of Yunnan Province and the Third People’s Hospital of Kunming City were enrolled in this study. Six chronic HBV carriers with a coexistence of HBsAg and anti-HBs antibodies were assigned to an experimental group and the other 6 matched chronic HBV-infected patients with negative for anti-HBs antibodies were a control group. No patients received the nucleotide/nucleoside analogue therapy. Written informed consent was obtained from all patients, and the study protocol was approved by the Ethics Committee of The second People’s Hospital of Yunnan Province in accordance with the Declaration of Helsinki. All patients met the following criteria: presence of HBsAg for at least 6 months, elevated HBV DNA levels (10^3^ IU/ml) with elevated or normal serum alanine aminotransferase (ALT) levels (upper limit of normal, 40 U/liter), and no signs of human immuno-deficiency virus, hepatitis C virus, or hepatitisvirus coinfection and decompensated liver disease (e.g., variceal bleeding, ascites, or encephalopathy). The experimental methods were carried out in accordance with the approved guidelines. All experimental protocols of this study were approved by the institutional review board/Ethics Committee of the Second People’s Hospital of Yunnan Province.

### Liver biochemistry, HBV serology, and HBV DNA tests

Liver biochemical (ALT and total bilirubinemia [TB]) and coagulation (prothrombin time [PT]) parameters were tested using an automated chemistry analysis system (Beckman Coulter, Fullerton, CA, USA). HBV serological markers were determined with a chemiluminescent microparticle immunoassay using the Abbott Architect immunoassay system (Abbott Laboratories, Abbott Park, IL, USA). The HBV DNA levels were measured by PCR using the Cobas Amplicor HBV monitor test (Roche Diagnostics, Mannheim, Germany), with a lower limit of quantification at 100 IU/ml. Only patients with serum HBV DNA levels more than 10^3^ IU/ml were included in this study (Table 1).

### Amplification and cloning of full-length HBV genomes

HBV genomes were extracted from 200 µl serum samples of treatment-naive patients using the Tianlong DNA/RNA virus mini-kit (Tianlong, Xi’an, Shaanxi, China). HBV full-length genomic DNA was amplified by PCR in a 50 µl volume containing 5 µl of HBV DNA template (20 ng/µl), 0.5 µl of high-fidelity LA TaqDNA Polymerase (TaKaRa Bio Inc., Shiga, Japan), 5 µl LA PCR buffer, 7 µl dNTPs, and 0.5 µl primer each [5′-TTT TTC ACC TCT GCC TAA TCA-3′ (forward, nt 1821–1841) and 5′-AAA AAG TTG CAT GGT GCT GG (reverse, nt 1825–1806)], which were designed in a previous study^15^. Amplification was performed under the following conditions: 95 °C for 3 min, 10 cycles at 94 °C for 40 s, 60 °C for 90 s, and 68 °C for 3 min, 5 min and 7 min successively, with a final extension of 72 °C for 10 min. PCR products of 3.5 kb were purified using the Tsingke gel extraction kit (Tsingke, Beijing, China), cloned into the pclone007 vector (Tsingke), and transformed into *Escherichia coli* strain DH5a competent cells (Tsingke). An average of 15 (range, 12 to 20) positive clones per sample were sequenced by six primers according to Yang *et al*.^46^ using an ABI 3730 automated sequencer (Applied Biosystems, Foster City, CA, USA). A total of 178 clones were sequenced from 12 treatment-naive HBV-infected patients.

### Sequence analysis

The quality of sequencing was analyzed using CodonCode aligner software (V. 3.7.1; CodonCode Corporation; http://www.codoncode.com/aligner) and all mutations were refined manually. The DNAstar Seqman software (DNASTAR Inc., Madison, WI, USA) was used to assemble the sequences into a complete HBV genome sequence. The following regions were aligned with the following reference sequences (GU815660, Genotype B; FJ562300, Genotype C; GU357844, Genotype I) with Muscle 3.8.31^50^ and then were divided into 14 regions according to Yang *et al*.^46^. The RDP3 software was used to identify potential recombinant sequences^51^. HBV genotyping was performed using the NCBI Genotyping tool (http://www.ncbi.nlm.nih.gov/projects/genotyping/form-page.cgi). HBV linear peptide epitopes were predicted by Immune Epitope Database and Analysis Resource (http://www.immuneepitope.org/). Newly determined genomes were deposited in GenBank (accession numbers: KY470830-KY471007).

### Quasispecies analysis

The characteristics of HBV quasispecies were evaluated at two levels: complexity and diversity quasispecies complexity is an index of the distribution of different variants in the viral population, and is expressed as normalized Shannon entropy (Sn)^52^. The complexity of each nucleotide position (nt1 to 3215) was calculated as the Shannon entropy [Sn = *i*∑_[A,T,C,G,-]_(pilnpi)/lnN] where pi represents the relative frequency of nucleotides or deletion at this position and N represents the total number of clones^53^. The Figure for complexity (Fig. 1) was generated using the Circos visualization tool^54^. QS diversity was evaluated using MEGA6^55^ with three parameters: the mean genetic distance (d), the number of synonymous substitutions per synonymous site (dS), and the number of non-synonymous substitutions per non-synonymous site (dN).

All clones were compared with the reference sequences mentioned above to identify variations in the core promoter and precore regions at the nucleotide level and variations in HBcAg, LHBsAg, HBxAg, and RT at the amino acid level.

### Phylogenetic analysis

Phylogenetic trees were reconstructed from either the HBV full-length genomes or the concatenated nucleotide alignments (four protein-coding: Pol, LHBsAg, HBxAg, and PreC/C). We employed ModelTest 3.7 to choose the (GTR + I + G) model of sequence evolution for the ML analysis, under the Akaike Information Criterion^56^. PhyML 3.0 was then used to create maximum likelihood phylogenies^57^ with 1000 bootstraps. MrBayes 3.1.2 was used for Bayesian inference^58^ and was conducted twice with four Markov chains each. Runs were conducted for 5 * 10^6^ generations and were sampled every 100 generations. When the log likelihood scores were found to stabilize, a consensus tree was calculated after omitting the first 25% of the trees as burn-in. Since both phylogenetic methods generated similar topologies, the MrBayes phylogeny is displayed only.

### Analysis of selection pressureand positive selection

Phylogenetic trees were reconstructed from the four concatenated protein-coding nucleotide alignments excluding sequences with stop codon mutations, deletions, and insertions. We used the dN/dS ratio, a measure of selection to measure the selection pressure acting on a lineage of HBV. The values of dN, dS, and the dN/dS ratio were estimated using the free-ratio model (model = 1, NSsites = 0, fi_omega = 0, omega = 1) in PAML4^59^. Lineage-specific mean values were estimated with concatenated alignments of all orthologs. SWAAP 1.0.2 was used to calculate dN/dS on a sliding window and scale (http://www.bacteriamuseum.org/SWAAP/SwaapPage.htm) using the Nei-Gojobori distance estimation method (window length, 15 nt; window step, 3 nt). The direction of selection pressure was determined by measuring the variable ω, representing the non-synonymous/synonymous substitution ratios (w = dN/dS) at each codon site, with values of ω < 1, ω = 1, and ω > 1 indicating purifying selection, neutral evolution, and positive selection, respectively. The extent of positive selection was analyzed using a site model employing two different pairs of models (M1/M2, M7/M8). Model M1 assumes negative selection and neutral evolution, while model M2 assumes an additional level of positive selection. The pair of model M7 and M8 assumes beta distributions for ω among sites, providing a sensitive test for positive selection. Likelihood ratio tests were used to compare the nested models, and empirical Bayes methodology was used to identify the amino acid sites under positive selection, due to a more reliable posterior probability calculation for small datasets^60^.

### Statistical analysis

The distribution of point mutations within the core promoter and precore regions at the nucleotide level and amino acid substitutions within the large HBsAg, HBcAg, HBxAg, and RT at the amino acid level among the two groups was analyzed using a chi-square test using SPSS 19.0. Quasispecies complexity or diversity was analyzed with a Student’s t-test or Wilcoxon rank sum test, and p-values of <0.05 were considered significant.

## Electronic supplementary material

Table supplementary
