# Supplementary material for: Evolution of full-length genomes of HBV quasispecies in sera of patients with a coexistence of HBsAg and anti-HBs antibodies
Source: Sci Rep. 2017 Apr 6;7:661. doi: 10.1038/s41598-017-00694-8 (PMC5428874; doi:10.1038/s41598-017-00694-8)
Supplement: Supplementary file 1 — Table supplementary [file 41598_2017_694_MOESM1_ESM.pdf]

Evolution of full-length genomes of HBV quasispecies in sera of patients with a coexistence of HBsAg and anti-HBs antibodies

Tai-Cheng Zhou[1a], Xiao Li[1a], Long Li[1], Xiao-Fei Li[2], Liang Zhang[1]*, Jia Wei[1]*

[1]Central lab, Liver disease research center, the second people's hospital of Yunnan Province, Kunming,Yunnan Province, China

[2]Clinical laboratory, the third people's hospital of Kunming City, Kunming,Yunnan Province, China

*E-mail: Liang_tkl@163.com

*E-mail: weijia19631225@163.com

[a]These authors contributed equally to this work.

Figure S1 Phylogenetic tree reconstructed for filtered sequences, which excluded sequences containing premature termination codons. This tree was used to calculate selective pressure. Phylogenetic trees were reconstructed from the concatenated four protein-coding nucleotide alignments excluding sequences with stop codon mutations, deletion and insertion frameshifts.

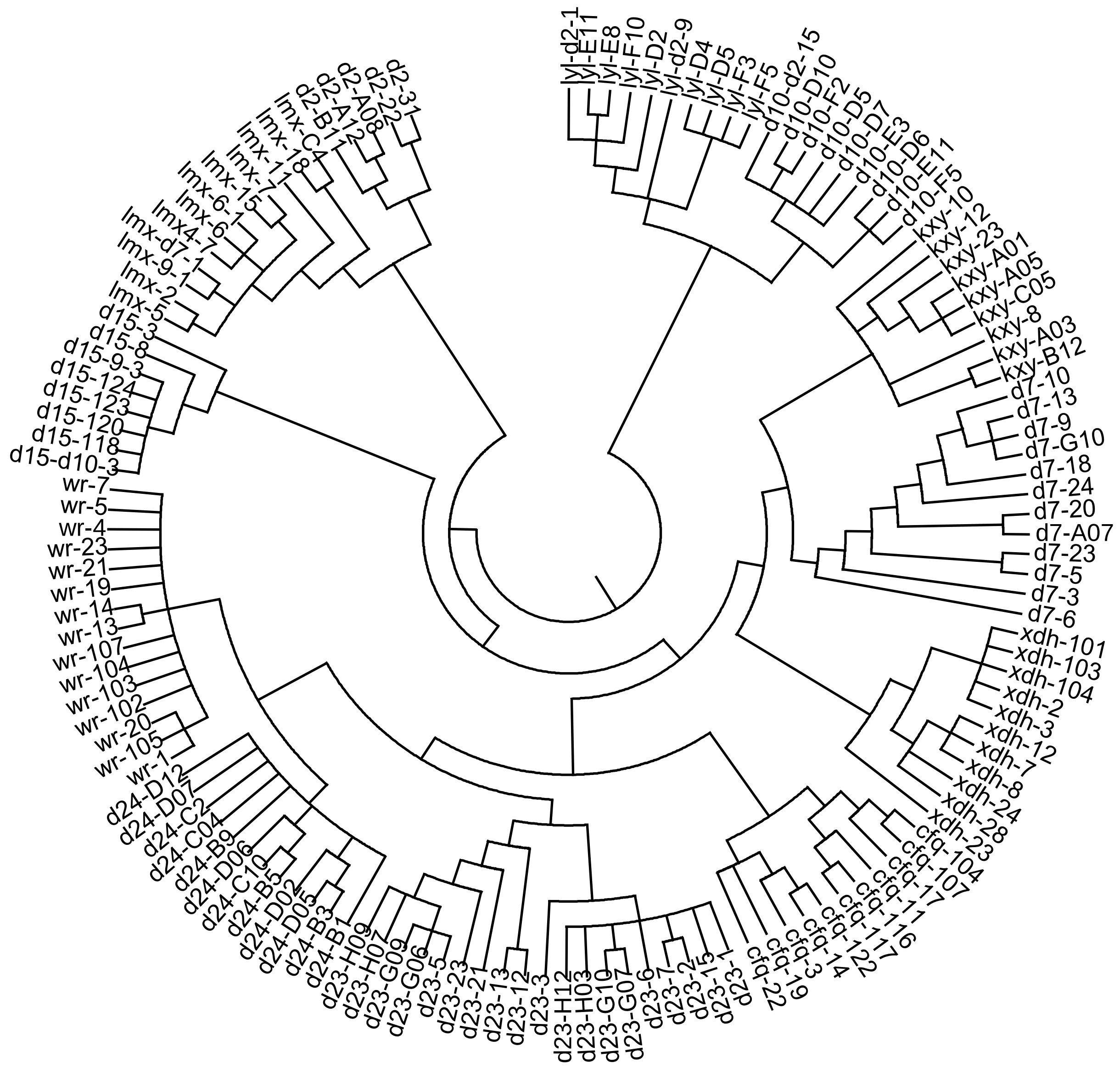

Table S1 Quasispecies complexity (Sn) of each nucleotide position (nt1 to 3215) between the patients with coexisting HBsAg and anti-HBs and controls.

Region of variation (level)	Experimental group(mean ± SE)(n=6)	Control group (mean ±SE)(n=6)	P value*
Genotype C			
Full length (nt)	0.0362±0.0012	0.0318±0.0013	0.001
Large HBsAg (nt)	0.0496±0.0027	0.021±0.0021	<0.001
Middle HBsAg (nt)	0.0343±0.003	0.0163±0.0022	<0.001
HBsAg (nt)	0.0305±0.0033	0.0137±0.0023	<0.001
HBxAg (nt)	0.0333±0.0023	0.0251±0.0025	0.002
Procore/core(nt)	0.027±0.0017	0.031±0.002	0.058
Polymerase (nt)	0.0474±0.0019	0.0259±0.0017	<0.001
RT region (nt)	0.0365±0.0011	0.0303±0.0011	0.001
Genotype B/C/I			
Full length (nt)	0.0484±0.0012	0.0387±0.0013	<0.001
Large HBsAg (nt)	0.0593±0.001	0.0406±0.0009	<0.001
Middle HBsAg (nt)	0.0588±0.0009	0.0403±0.0008	<0.001
HBsAg (nt)	0.0581±0.0009	0.0398±0.0008	<0.001
HBxAg (nt)	0.0578±0.0008	0.0401±0.0008	<0.001
Procore/core(nt)	0.0566±0.0009	0.0412±0.0008	<0.001
Polymerase (nt)	0.0551±0.001	0.0405±0.001	<0.001
RT region (nt)	0.0566±0.0008	0.0413±0.0008	<0.001

*Paired-samples T test

Table S2 Epitopes for HBxAg, LHBsAg, PreC/C and Polymerase.

Epitope ID	Linear sequence	nt Starting Position	nt Ending Position	Antigen Name	Epitope types
34101	VGAESCGRPFSGSLG	1434	1478	HBxAg	HLAI T cell epitopes
37187	KVLHKRTLGLSAMST	1644	1688	HBxAg	HLAI T cell epitopes
36354	CAFSSAGPCALRFTS	1554	1598	HBxAg	HLAII T cell epitopes
31898	QLDPARDVL	1395	1421	HBxAg	HLAI T cell epitopes
34027	VLCLRPVGA	1416	1442	HBxAg	HLAI T cell epitopes
36402	LRFTSARRMETTVN	1584	1625	HBxAg	HLAI T cell epitopes
37181	TTVNAHQILPKVLHK	1614	1658	HBxAg	HLAI T cell epitopes
37466	KVLHKRTLGL	1644	1673	HBxAg	HLAI T cell epitopes
37919	VLHKRTLGL	1647	1673	HBxAg	HLAI T cell epitopes
38701	SAMSTTDLEAYFKDC	1674	1718	HBxAg	HLAI T cell epitopes
40624	EIRLMIFVLGGCRHK	1749	1793	HBxAg	HLAI T cell epitopes
40664	ETRLMIFVLGGCRHK	1749	1793	HBxAg	HLAI T cell epitopes
28447	MAARLCCQLDPARDV	1374	1418	HBxAg	HLAII T cell epitopes
34102	SGSLGTLSSPSPSAV	1464	1508	HBxAg	HLAII T cell epitopes
37786	KVLHKRTLGLSAMSTT	1644	1691	HBxAg	HLAII T cell epitopes
37943	GLSAMSTTDL	1668	1697	HBxAg	HLAII T cell epitopes
39347	YFKDCLFKDWEELGEE	1704	1751	HBxAg	HLAII T cell epitopes
39706	CLFKDWEEL	1716	1742	HBxAg	HLAII T cell epitopes
41238	VFVLGGCRHKLVCAPAPC	1764	1823	HBxAg	HLAII T cell epitopes
42384	VLGGCRHKL	1770	1796	HBxAg	HLAII T cell epitopes
42443	KLVCSPAPC	1791	1817	HBxAg	HLAII T cell epitopes
35398	HLSLRGLFV	1527	1553	HBxAg	B cell epitopes
39089	YFKDCLFKDWEELGE	1704	1748	HBxAg	B cell epitopes
40283	EELGEEIRLKVFVLG	1734	1778	HBxAg	B cell epitopes
41004	VFVLGGCRHKLVCAP	1764	1808	HBxAg	B cell epitopes
41238	VFVLGGCRHKLVCAPAPC	1764	1823	HBxAg	B cell epitopes
42443	KLVCSPAPC	1791	1817	HBxAg	B cell epitopes
44684	LVCAPAPCNFFTSA	1794	1835	HBxAg	B cell epitopes
34103	SPSAVPTDHGAHLSL	1494	1538	HBxAg	B cell epitopes
2758	FLGPLLVLQA	143	172	LHBsAg	HLAI T cell epitopes
6556	FFLLTRILTI	176	205	LHBsAg	HLAI T cell epitopes
6631	FLLTRILTI	179	205	LHBsAg	HLAI T cell epitopes
16755	STGPCKTCTT	503	532	LHBsAg	HLAI T cell epitopes
16795	SMYPSCCCTK	515	544	LHBsAg	HLAI T cell epitopes
16796	CTTPAQGNSMFPSC	524	565	LHBsAg	HLAI T cell epitopes
16836	MFPSCCCT	551	574	LHBsAg	HLAI T cell epitopes
16857	IPIPSSWAF	569	595	LHBsAg	HLAI T cell epitopes
21139	LWEWASVRF	605	631	LHBsAg	HLAI T cell epitopes
21145	SWLSLLVPF	632	658	LHBsAg	HLAI T cell epitopes
21146	YLWEWASVR	635	661	LHBsAg	HLAI T cell epitopes
23151	GLSPTVWLSV	674	703	LHBsAg	HLAI T cell epitopes
24299	PFMPLLPIFF	752	781	LHBsAg	HLAI T cell epitopes
423053	TNLSVPNPLGFFPDHQLDP	2887	2943	LHBsAg	HLAI T cell epitopes
423063	PLGFFPDHQLDPAFGANSN NPDWDFNP	2908	2988	LHBsAg	HLAI T cell epitopes
462003	PTPFSPPLRD	3160	3189	LHBsAg	HLAI T cell epitopes
462107	MQWNSTTFHQTLQDPRVR GLYFPAGG	3172	34	LHBsAg	HLAI T cell epitopes
462159	NSTTFHQTLQDPRVRGLYF PAGGSSSGTVNPVPTTVSPI SSIFSRIGD	3181	109	LHBsAg	HLAI T cell epitopes
5911	VLQAGFFLL	161	187	LHBsAg	HLAII T cell epitopes
5976	AGFFLLTRILTIPQS	170	214	LHBsAg	HLAII T cell epitopes
7328	GGTPACPG	281	304	LHBsAg	HLAII T cell epitopes
11748	GYRWMCLRR	365	391	LHBsAg	HLAII T cell epitopes
11997	ILLLCLIFL	377	403	LHBsAg	HLAII T cell epitopes
12566	ILLLCLIFLL	377	406	LHBsAg	HLAII T cell epitopes
15063	LLDYQGMLPV	410	439	LHBsAg	HLAII T cell epitopes
22023	LLVPFVQWFV	644	673	LHBsAg	HLAII T cell epitopes
22025	LVPFVQWFVGLSPTV	647	691	LHBsAg	HLAII T cell epitopes
23991	SVIWMMWYW	698	724	LHBsAg	HLAII T cell epitopes

1799	SSGTVSPAQNTVSAISSI	71	124	LHBsAg	B cell epitopes
6632	FLLTKILTI	212	238	LHBsAg	B cell epitopes
7332	SQISSHSPTCCPPICPGYRW	317	376	LHBsAg	B cell epitopes
11748	GYRWMCLRR	365	391	LHBsAg	B cell epitopes
15061	VIWMMWYWGR	386	415	LHBsAg	HLAI T cell epitopes
16814	CTIPAQGTSVFPSCCCTKPS DGNC	524	595	LHBsAg	HLAI T cell epitopes
16815	CTIPAQGTSMFPSCCCTKPS DGNC	524	595	LHBsAg	HLAI T cell epitopes
21242	FLWEWASA	635	658	LHBsAg	HLAI T cell epitopes
18802	CTKPTDGNC	569	595	LHBsAg	HLAII T cell epitopes
5191	TPARVTGGVF	151	180	Polymeras	HLAI T cell epitopes
15879	SGLPRYVARL	445	474	Polymeras	HLAI T cell epitopes
16751	GLSRYVARL	448	474	Polymeras	HLAI T cell epitopes
16753	GLSRYVARLS	448	477	Polymeras	HLAI T cell epitopes
16832	YIDDVVLGA	541	567	Polymeras	HLAI T cell epitopes
16833	YVDDVVLGA	541	567	Polymeras	HLAI T cell epitopes
19351	TYGRKLHLYSHPIILGFRKI	577	636	Polymeras	HLAI T cell epitopes
21070	LHLYSHPIILGFRKI	592	636	Polymeras	HLAI T cell epitopes
21116	KLHLYSHPI	595	621	Polymeras	HLAI T cell epitopes
24302	FLLSLGIHL	808	834	Polymeras	HLAI T cell epitopes
24943	KQCFRKLPVNRPIDW	937	981	Polymeras	HLAI T cell epitopes
27168	YPALMPLSACIQAKR	1042	1086	Polymeras	HLAI T cell epitopes
27169	QAFTFSPTYK	1051	1080	Polymeras	HLAI T cell epitopes
27285	KQAFTFSPTYKAFLC	1081	1125	Polymeras	HLAI T cell epitopes
27365	LCQVFADATPTGWGL	1171	1215	Polymeras	HLAI T cell epitopes
27878	YMDDVVLGA	1283	1309	Polymeras	HLAI T cell epitopes
32955	ILRGTSFVYV	1402	1431	Polymeras	HLAI T cell epitopes
34616	SLYADSPSV	1498	1524	Polymeras	HLAI T cell epitopes
226775	NVSIPWTHK	2445	2471	Polymeras	HLAI T cell epitopes
226786	KVGNFTGLY	2469	2495	Polymeras	HLAI T cell epitopes
226809	GLYSSTVPV	2487	2513	Polymeras	HLAI T cell epitopes
422990	VGPLTVNEKRRLKLI	2592	2636	Polymeras	HLAI T cell epitopes
422997	RHYLHTLWKAGILYK	2739	2783	Polymeras	HLAI T cell epitopes
423031	HTLWKAGILYK	2751	2783	Polymeras	HLAI T cell epitopes
423032	TLWKAGILYK	2754	2783	Polymeras	HLAI T cell epitopes
423036	ESTRSASFCGSPYSW	2787	2831	Polymeras	HLAI T cell epitopes
5191	TPARVTGGVF	151	180	Polymeras	HLAII T cell epitopes
7171	ESRLVVDFSQFSRGN	244	288	Polymeras	HLAII T cell epitopes
7230	VVDFSQFSR	262	288	Polymeras	HLAII T cell epitopes
8608	LQSLTNLLSSNLSWL	325	369	Polymeras	HLAII T cell epitopes
9815	SSNLSWLSLDVSAAF	349	393	Polymeras	HLAII T cell epitopes
15848	GVSRYVARL	415	441	Polymeras	HLAII T cell epitopes
15957	GLPRYVARL	448	474	Polymeras	HLAII T cell epitopes
16833	YVDDVVLGA	541	567	Polymeras	HLAII T cell epitopes
25650	ALMPLYACI	1015	1041	Polymeras	HLAII T cell epitopes
30373	AANWILRGTSFVYVP	1390	1434	Polymeras	HLAII T cell epitopes
7161	VDKNPHNTTESRLVV	217	261	Polymeras	B cell epitopes
7162	LVVDFSQFSR	220	249	Polymeras	B cell epitopes
7171	ESRLVVDFSQFSRGN	244	288	Polymeras	B cell epitopes
23068	SAICSVVRR	649	675	Polymeras	B cell epitopes
51311	GATVELLSFLPSDFF	1928	1972	PreC/C	HLAI T cell epitopes
56814	FLPSDFFPSI	1952	1981	PreC/C	HLAI T cell epitopes
59585	PSDFFPSVRDLLDTA	1958	2002	PreC/C	HLAI T cell epitopes
59786	SDFFPSVRDLLDTASALYR	1961	2020	PreC/C	HLAI T cell epitopes
61741	LLDTASALY	1988	2014	PreC/C	HLAI T cell epitopes
62477	YRDALESPEHCSPHHTA	2012	2062	PreC/C	HLAI T cell epitopes
62504	ALESPEHCSPHHTALRQAIL	2021	2080	PreC/C	HLAI T cell epitopes
65148	LESQDHCSP	2024	2050	PreC/C	HLAI T cell epitopes
65509	PHHTALRQAILCWGELMT	2048	2107	PreC/C	HLAI T cell epitopes
69398	TWVGTNLEDPASRDLVVS	2108	2167	PreC/C	HLAI T cell epitopes
69474	WVGVNLEDPASRDLVVSY	2111	2170	PreC/C	HLAI T cell epitopes
69500	VGSNLEDPASRELVVSYVN	2114	2170	PreC/C	HLAI T cell epitopes
69524	GVNLEDPASRDL	2117	2152	PreC/C	HLAI T cell epitopes
71631	TNLEDPASRDLVVSY	2120	2164	PreC/C	HLAI T cell epitopes

71847	NLEDPASRDL	2123	2152	PreC/C	HLAI T cell epitopes
74180	ASRDLVVSYVNTNMGLKF RQLLWFHISCLTFGRETVIE	2138	2254	PreC/C	HLAI T cell epitopes
75040	VVSYVNTNMGLKFRQL	2153	2200	PreC/C	HLAI T cell epitopes
76245	YVNTNMGLK	2162	2188	PreC/C	HLAI T cell epitopes
76370	YVNVNMGLK	2162	2188	PreC/C	HLAI T cell epitopes
79601	LWFHISCLMF	2201	2230	PreC/C	HLAI T cell epitopes
93224	GRETVIEYLVSFGVW	2231	2275	PreC/C	HLAI T cell epitopes
93431	GRETVLEYLVSFGVW	2231	2275	PreC/C	HLAI T cell epitopes
93536	RETVIEYLVSF	2234	2266	PreC/C	HLAI T cell epitopes
110917	VSFGVWIRTPPA	2258	2293	PreC/C	HLAI T cell epitopes
178829	VSFGVWIRTPPAYRPPNAPI	2258	2317	PreC/C	HLAI T cell epitopes
187197	LSTLPETTVVRRRGRSPRR RTPSPR	2318	2392	PreC/C	HLAI T cell epitopes
190409	STLPETTVVRR	2321	2353	PreC/C	HLAI T cell epitopes
190443	STLPETTVV	2321	2347	PreC/C	HLAI T cell epitopes
190455	STLPETTVVRRRGRSPRRR	2321	2380	PreC/C	HLAI T cell epitopes
190488	TVVRRRGRSPRRRTP	2339	2383	PreC/C	HLAI T cell epitopes
190509	TVVRRRGRSP	2339	2368	PreC/C	HLAI T cell epitopes
190556	RRRGRSPRRR	2348	2377	PreC/C	HLAI T cell epitopes
190568	RRRGRSPRRRTPSPRRRRS QSPRRRRSQSRESQC	2348	2449	PreC/C	HLAI T cell epitopes
190569	GRSPRRRTPSPRRRRSQSPR RRRSQSRESQC	2357	2449	PreC/C	HLAI T cell epitopes
190577	SPRRRTPSPRRRRSQ	2363	2407	PreC/C	HLAI T cell epitopes
190589	PSPRRRRS	2381	2404	PreC/C	HLAI T cell epitopes
190596	SPRRRRSQSPRRRRS	2384	2428	PreC/C	HLAI T cell epitopes
191095	QSPRRRRSQSRESQC	2405	2449	PreC/C	HLAI T cell epitopes
47828	MDIDPYKEFGATVELLSFL	1901	1960	PreC/C	HLAII T cell epitopes
47829	DIDPYKEFGATVELLSFLPS DFFPSVRDLLDTASA	1904	2008	PreC/C	HLAII T cell epitopes
47830	DIDPYKEFGATVELL	1904	1948	PreC/C	HLAII T cell epitopes
47832	IDPYKEFGATVELLS	1907	1951	PreC/C	HLAII T cell epitopes
47833	DPYKEFGATVELLSF	1910	1954	PreC/C	HLAII T cell epitopes
47834	PYKEFGATVELLSFL	1913	1957	PreC/C	HLAII T cell epitopes
47836	YKEFGATVELLSFLP	1916	1960	PreC/C	HLAII T cell epitopes
48757	KEFGASVELL	1919	1948	PreC/C	HLAII T cell epitopes
49308	EFGATVELLSFLPSD	1922	1966	PreC/C	HLAII T cell epitopes
50118	FGATVELLSFLPSDF	1925	1969	PreC/C	HLAII T cell epitopes
52404	ATVELLSFLPSDFFPSV	1931	1981	PreC/C	HLAII T cell epitopes
53382	TVELLSFLPSDFFPS	1934	1978	PreC/C	HLAII T cell epitopes
53383	VELLSFLPSDFFPSV	1937	1981	PreC/C	HLAII T cell epitopes
53394	LLSFLPSDFFPSVRD	1943	1987	PreC/C	HLAII T cell epitopes
58876	LPSDFFPSV	1955	1981	PreC/C	HLAII T cell epitopes
60227	FFPSIRDLL	1967	1993	PreC/C	HLAII T cell epitopes
60229	FPSVRDLLDTASALYREAL ESPEHCSPHHTA	1970	2062	PreC/C	HLAII T cell epitopes
61746	LYREALESPEHCSPHHTAL	2009	2074	PreC/C	HLAII T cell epitopes
65475	PHHTALRQAI	2048	2077	PreC/C	HLAII T cell epitopes
67204	LCWGELMTLATWVGVNL EDPASRDL	2078	2152	PreC/C	HLAII T cell epitopes
67300	LCWGELMTLATWVGVNL	2078	2128	PreC/C	HLAII T cell epitopes
68276	VCWGELMNL	2078	2104	PreC/C	HLAII T cell epitopes
68599	CWGELMTLATWVGVNLE	2081	2155	PreC/C	HLAII T cell epitopes
70928	VNLEDPASRDL	2120	2152	PreC/C	HLAII T cell epitopes
70929	VNLEDPASRDLVVSYVNT	2120	2200	PreC/C	HLAII T cell epitopes
71847	NLEDPASRDL	2123	2152	PreC/C	HLAII T cell epitopes
73842	PHHTALRQAILCWGD	2135	2179	PreC/C	HLAII T cell epitopes
73843	PHHTALRQAILCWGDLMN	2135	2194	PreC/C	HLAII T cell epitopes
75585	VSYVNTNMGLKFRQL	2156	2200	PreC/C	HLAII T cell epitopes
93536	RETVIEYLVSF	2234	2266	PreC/C	HLAII T cell epitopes
48337	KEFGATVELLSFLPS	1919	1963	PreC/C	B cell epitopes
55712	FLPADFFPSV	1952	1981	PreC/C	B cell epitopes

55713	FLPSDFFPSV	1952	1981	PreC/C	B cell epitopes
60249	RDLLDTAAALYRDALESPE	1982	2041	PreC/C	B cell epitopes
61746	LYREALESPEHCSPHHTAL	2009	2074	PreC/C	B cell epitopes
65360	HHHTALRQAILCWGELMN	2048	2107	PreC/C	B cell epitopes
68600	CWGDLMTLATWVGTNLE	2081	2140	PreC/C	B cell epitopes
68618	GELMTLATW	2087	2113	PreC/C	B cell epitopes
71938	LEDPASRDLVVSY	2126	2164	PreC/C	B cell epitopes
72649	LEDPASRDLVVSYV	2126	2167	PreC/C	B cell epitopes
73843	PHHTALRQAILCWGDLMN	2135	2194	PreC/C	B cell epitopes
75662	SYVNTNMGLKFRQLLWFH ISCLTFGRETVIEY	2159	2254	PreC/C	B cell epitopes
79531	NVNMGLKIRQ	2168	2197	PreC/C	B cell epitopes
79549	KFRQLLWFHISCLTFGRET	2186	2254	PreC/C	B cell epitopes
79577	LLWFHISCLTFGRETVLEY	2198	2257	PreC/C	B cell epitopes
79583	LWFHISCLTFGRETVIEYLV	2201	2260	PreC/C	B cell epitopes
79594	LWFHISCLTF	2201	2230	PreC/C	B cell epitopes
79613	HISCLTFGR	2210	2236	PreC/C	B cell epitopes
79654	CLTFGRETV	2219	2245	PreC/C	B cell epitopes
79672	CLTFGRETVIEY	2219	2254	PreC/C	B cell epitopes
79673	LTFGRETVLEYLVSF	2222	2266	PreC/C	B cell epitopes
96088	VLEYLVSFGV	2243	2272	PreC/C	B cell epitopes
96630	EYLVSFGVWIRTPPA	2249	2293	PreC/C	B cell epitopes
96862	EYLVSFGVW	2249	2275	PreC/C	B cell epitopes
97104	LVSFGVWIR	2255	2281	PreC/C	B cell epitopes
180906	SFGVWIRTPPAYRPPNAPIL	2261	2320	PreC/C	B cell epitopes
180913	FGVWIRTPPAYR	2264	2299	PreC/C	B cell epitopes
180914	VWIRTPPAYR	2270	2299	PreC/C	B cell epitopes
180915	WIRTPPAYR	2273	2299	PreC/C	B cell epitopes
180916	IRTPPAYRPPNAPILSTLPET	2276	2344	PreC/C	B cell epitopes
180917	IRTPPAYRPPNAPILSTLPET	2276	2338	PreC/C	B cell epitopes
180928	PAYRPPNAPIL	2288	2320	PreC/C	B cell epitopes
180929	AYRPPNAPILSTLPE	2291	2335	PreC/C	B cell epitopes
180930	YRPPNAPILSTLPETT	2294	2341	PreC/C	B cell epitopes
56900	FLPPDFFPSV	1952	1981	PreC/C	HLAII T cell epitopes
56900	FLPPDFFPSV	1952	1981	PreC/C	B cell epitopes
46480	ISCSCPTVQASKLCLGWLW GMD	1841	1906	PreC/C	HLAI T cell epitopes
50253	FGATVELLSFLPSDFFPSV	1925	1981	PreC/C	HLAI T cell epitopes
58122	FLPADFFPSI	1952	1981	PreC/C	HLAI T cell epitopes
61745	PHHTALRQAIVCWGELMT	1997	2056	PreC/C	HLAI T cell epitopes
69715	GGNLEDPISRDLVVSYVNT	2117	2173	PreC/C	HLAI T cell epitopes
190466	STLPETTVVRQ	2321	2353	PreC/C	HLAI T cell epitopes
46927	SCPTVQASKLCLGWLWG	1850	1900	PreC/C	HLAII T cell epitopes
47826	SKLCLGWLWGMD	1871	1906	PreC/C	HLAII T cell epitopes
47827	LGWLWGMDIDPYKEF	1883	1927	PreC/C	HLAII T cell epitopes
57129	FLPVDFFPSV	1952	1981	PreC/C	HLAII T cell epitopes
66988	PHHTALRQAILCWGDLMT	2048	2107	PreC/C	HLAII T cell epitopes
67096	PHHTALRQAILCWGELMN	2048	2107	PreC/C	HLAII T cell epitopes
67203	PHHTALRQAVLCWGELMT	2048	2107	PreC/C	HLAII T cell epitopes
74445	RDLVVSYVNTNVGLKFRQ	2144	2203	PreC/C	HLAII T cell epitopes
46013	MQLFHLCLIISCTCPTVQAS	1814	1873	PreC/C	B cell epitopes
46927	SCPTVQASKLCLGWLWG	1850	1900	PreC/C	B cell epitopes
57784	FLPNDFFPSV	1952	1981	PreC/C	B cell epitopes
66895	PHHTALRQAILCWGDLMN	2048	2107	PreC/C	B cell epitopes
79521	VNTNMGLKFRQLLWFHIS	2165	2221	PreC/C	B cell epitopes
79546	TNVGLKFRQLLWFHISCLT	2171	2230	PreC/C	B cell epitopes
79561	FRQLLWFHISCLTFGRETVI	2189	2257	PreC/C	B cell epitopes
79609	FHISCLTFGRETVIEYLVSF	2207	2269	PreC/C	B cell epitopes
79677	TFGRETVIEYLVSFGVWIR	2225	2281	PreC/C	B cell epitopes
180919	PPAYRPPNAPILSTL	2285	2329	PreC/C	B cell epitopes
180931	ILSTLPETTV	2315	2344	PreC/C	B cell epitopes
58234	FLPSDFLPSV	1952	1981	PreC/C	B cell epitopes

note: LHBsAg, large HBsAg; PreC/C, precore/core.

Table S3 dN/dS ratios of genome, pol, HbxAg, PreC/C and LHBsAg.

Groups	Patients No	dN/dS Genome	Pol	HBxAg	PreC/C	LHBsAg
Experimental	d10	0.7288	0.626	0.0001	0.0001	425.2716
Experimental	d10	0.4379	0.0001	0.364	0.0001	339.4327
Experimental	d10	0.0001	10.397	0.3556	0.0001	0.0001
Experimental	d10	0.6569	999	0.3625	0.0001	0.4561
Experimental	d10	0.8765	1.2549	0.0001	0.0001	339.6112
Experimental	d10	2.6121	1.2346	999	0.0001	7.0964
Experimental	d10	0.0001	8.3203	0.3084	0.0001	0.0001
Experimental	d10	0.0001	6.6045	0.3107	0.0001	0.0001
Experimental	d10	1.3149	0.4171	0.3064	870.3481	425.6478
Experimental	d15	0.0001	8.4696	0.3116	0.0508	0.0001
Experimental	d15	0.0001	8.5683	0.3166	0.1047	0.0001
Experimental	d15	0.0001	12.9322	0.3128	0.0852	0.0001
Experimental	d15	0.0001	7.7717	0.3148	0.251	0.0001
Experimental	d15	0.0001	7.5559	0.3152	0.037	0.0001
Experimental	d15	0.0001	9.2665	0.3115	0.0881	0.0001
Experimental	d15	1.0444	0.5089	0.3182	0.56	487.2994
Experimental	d15	0.6654	0.3098	1.3778	0.0001	6.6585
Experimental	d2	0.4333	0.1648	993.5894	0.0761	422.4457
Experimental	d2	0.0001	6.7914	0.3008	0.0629	0.0001
Experimental	d2	173.0408	13.7259	0.3091	875.8592	0.0001
Experimental	d2	1.311	999	0.0001	0.0804	338.4502
Experimental	d2	0.8708	0.8308	0.4575	0.8376	337.7533
Experimental	d23	1.0202	0.4146	999	0.0001	485.2943
Experimental	d23	0.0001	0.0001	0.3429	0.1028	0.0001
Experimental	d23	0.0001	0.0001	0.3377	0.0001	0.0001
Experimental	d23	0.0001	0.0001	0.3398	0.0001	0.0001
Experimental	d23	326.9184	999	996.5506	873.0142	425.0353
Experimental	d23	0.0001	0.0001	0.3262	0.1159	0.2285
Experimental	d23	0.0001	0.0001	0.334	0.0988	0.0001
Experimental	d23	2.1868	999	998.535	0.0001	0.919
Experimental	d23	0.2621	0.2766	0.3364	0.0695	534.7221
Experimental	d23	0.1456	0.1382	0.3455	0.0001	1.3785
Experimental	d23	0.6567	999	0.3234	0.1025	0.0001
Experimental	d23	1.4605	2.5031	0.3355	0.1036	0.4591
Experimental	d23	390.536	999	999	0.0001	0.0001
Experimental	d23	0.4367	0.4142	0.4589	0.4184	0.0001
Experimental	d23	0.4382	999	0.3225	0.0436	486.5935
Experimental	d23	1.1705	2.0873	997.9338	0.1262	0.2285
Experimental	d23	0.6578	0.8339	999	0.1243	425.1518
Experimental	d23	1.3162	0.8345	999	0.0631	0.0001
Experimental	d23	2.6322	0.833	998.6699	0.1581	0.0001
Experimental	d24	1.7552	0.4175	0.3134	863.7041	426.0316
Experimental	d24	222.284	811.4317	0.3112	0.0001	340.8202
Experimental	d24	0.0001	0.0001	0.3101	999	0.0001
Experimental	d24	257.6399	1.2483	0.3188	0.0001	0.0001
Experimental	d24	0.0001	1.6619	0.3423	0.0001	0.0001
Experimental	d24	0.0001	1.6666	0.3422	0.0001	0.0001
Experimental	d24	0.0001	0.0001	0.3409	0.0001	0.0001
Experimental	d24	0.7311	999	0.0001	864.7285	0.0001
Experimental	d24	0.5847	0.2778	0.3148	0.0001	426.2213
Experimental	d24	0.6577	1.2522	0.3094	0.0001	0.0001
Experimental	d24	222.2845	810.7659	0.3096	0.0001	340.517
Experimental	d24	1.7548	999	0.0001	0.0001	426.133
Experimental	d7	0.5235	0.8238	0.0001	0.1395	0.4602
Experimental	d7	0.4365	999	0.3128	0.0001	0.0001
Experimental	d7	0.3491	0.412	0.4584	0.1132	0.0001
Experimental	d7	2.183	999	0.3186	0.0743	0.4605
Experimental	d7	0.727	2.4769	0.0001	0.1043	0.0001
Experimental	d7	0.6011	0.2064	0.3478	0.4192	426.4093
Experimental	d7	0.421	0.2742	998.3971	876.5726	0.0001
Experimental	d7	1.32	999	0.3087	0.4189	0.0001
Experimental	d7	0.5222	0.5776	994.8217	0.1476	0.3643
Experimental	d7	0.6957	0.6855	999	0.174	0.6879
Experimental	d7	1.7474	1.0988	0.2276	0.1035	3.2281
Experimental	d7	1.0763	0.5464	0.0001	999	1.373
Control	cfq	0.0001	6.9379	0.3282	0.103	0.0001
Control	cfq	0.0001	4.5017	0.3377	0.1054	0.0001
Control	cfq	0.2186	0.0001	0.2266	0.1177	0.0001
Control	cfq	0.3282	0.416	0.0001	876.1373	0.0001

Control	cfq	0.4041	0.1039	0.0001	989.1077	425.7829
Control	cfq	0.7662	0.4162	996.2854	0.6405	0.0001
Control	cfq	1.1697	0.6955	0.2577	0.0501	486.1918
Control	cfq	0.3749	0.4161	0.0001	878.2697	0.9184
Control	cfq	1.3152	0.4165	998.2267	874.4811	0.0001
Control	cfq	1.7562	0.6939	999	876.5491	534.0365
Control	cfq	307.1197	999	0.3384	877.5599	340.1642
Control	kxy	1.3118	999	0.4597	0.193	0.0001
Control	kxy	0.8742	0.4123	999	0.1961	1.8528
Control	kxy	2.1902	999	0.3602	883.6503	0.4607
Control	kxy	0.0001	0.0001	0.34	0.2523	0.0001
Control	kxy	0.0001	0.0001	0.3442	0.2292	0.0001
Control	kxy	0.0001	0.0001	0.3417	0.2784	0.0001
Control	kxy	1.6052	999	0.3545	0.2127	1.3859
Control	kxy	0.0001	0.0001	0.3629	0.087	0.0001
Control	kxy	0.0001	0.0001	0.3554	0.1593	0.0001
Control	lmx	0.0001	0.0001	0.3228	0.1875	0.0001
Control	lmx	1.0912	1.0428	0.3289	0.1575	0.0001
Control	lmx	172.3826	0.0001	0.3485	877.8005	0.0001
Control	lmx	0.0001	17.8258	0.3541	0.0248	0.0001
Control	lmx	0.0001	0.0001	0.3325	0.1897	0.0001
Control	lmx	0.0001	0.0001	0.3426	0.1399	0.0001
Control	lmx	0.0001	11.1316	0.3445	0.1584	0.0001
Control	lmx	0.0001	0.0001	0.3102	0.1132	0.0001
Control	lmx	0.0001	0.0001	0.3129	0.1647	0.0001
Control	lmx	254.1254	999	0.3431	0.1722	338.2032
Control	lmx	1.3088	0.0001	998.3381	999	338.7426
Control	lmx	0.2175	807.6278	0.0001	0.0001	0.0001
Control	lyl	256.147	999	0.4189	0.0001	339.2307
Control	lyl	0.0001	0.0001	0.4199	0.0001	0.0001
Control	lyl	0.4367	0.0001	0.4136	0.0001	0.0001
Control	lyl	0.6548	807.5884	999	0.0001	0.2263
Control	lyl	0.3734	2.0821	0.4096	0.0001	0.4529
Control	lyl	0.2181	999	0.4015	0.0001	338.6178
Control	lyl	0.0001	9.8987	0.4018	0.0001	0.0001
Control	lyl	0.0001	13.9145	0.4097	0.0001	0.0001
Control	lyl	1.3103	0.4169	0.4047	0.1082	423.5669
Control	lyl	0.0001	8.523	0.4057	872.326	0.0001
Control	wr	0.0001	3.0271	0.3051	0.1253	0.0001
Control	wr	0.0001	47.2565	0.3089	0.0567	0.0001
Control	wr	0.0001	2.6024	0.3039	0.0657	0.0001
Control	wr	0.7317	0.4184	997.1649	0.1035	424.8489
Control	wr	0.4385	0.4183	0.9132	0.1093	0.0001
Control	wr	0.878	0.0001	995.74	0.0982	339.7319
Control	wr	2.1963	1.6752	0.2948	0.1625	340.0721
Control	wr	0.0001	29.5669	0.3113	0.142	0.0001
Control	wr	0.0001	28.9053	0.3073	0.1442	0.0001
Control	wr	0.4387	0.1043	0.3093	999	485.1787
Control	wr	1.757	1.6754	0.3022	876.2383	0.4548
Control	wr	0.8779	0.4182	0.3105	0.1103	339.7
Control	wr	0.548	0.2782	999	0.0975	0.4557
Control	wr	0.0001	13.7643	0.3101	0.0677	0.0001
Control	wr	0.4386	0.0001	0.3086	0.0001	339.6812
Control	xdh	0.0001	8.6739	0.322	0.1274	0.0001
Control	xdh	0.0001	8.1242	0.3177	0.0842	0.0001
Control	xdh	0.0001	11.8047	0.3221	0.0001	0.0001
Control	xdh	0.0001	9.329	0.315	0.0758	0.0001
Control	xdh	0.0001	0.3086	0.3186	0.1182	0.0001
Control	xdh	0.0001	12.5591	0.3197	0.1094	0.0001
Control	xdh	0.0001	0.0001	0.3155	0.071	0.0001
Control	xdh	0.0001	7.7991	0.3107	0.0001	0.0001
Control	xdh	0.2189	0.4178	0.0001	0.0727	0.0001
Control	xdh	0.8769	999	0.324	0.0614	0.0001
Control	xdh	0.2642	0.8379	0.0001	0.0001	0.1522

Table S4 Summary of positive selection analyses for genes LHBsAg, HBxAg, PreC/C and Pol.

Gene	LHBsAg	HBxAg	PreC/C	Pol
**Model 1**				
lnL	-4900.388	-1997.998	-2309.669	-10905.244
d_N/d_S (p_0)	0.099(0.470)	0.047(0.511)	0.095(0.693)	0.118(0.696)
d_N/d_S (p_1)	1.000(0.51)	1.000(0.489)	1.000(0.307)	1.000(0.304)
**Model 2**				
lnL	-4898.999	-1986.074626	-2308.911	-10892.446
d_N/d_S (p_0)	0.23(0.667)	0.11(0.532)	0.113(0.713)	0.138(0.71)
d_N/d_S (p_1)	1.000(0.000)	1.000(0.379)	1.000(0.274)	1.000(0.267)
d_N/d_S (p_2)	1.412(0.333)	4.148(0.09)	3.41(0.013)	3.401(0.023)
2ΔlnL(LRT) (2vs1)	2.778	23.846748	1.516	25.596
P Value	0.096	**1.04E-06**	0.218	**4.21E-07**
Postitively selected sites*		36A,130K,131V		803R,841R
**Model 7**				
lnL	-4900.904	-1998.123	-2309.669	-10910.344
p	0.109	0.02	0.182	0.211
q	0.075	0.017	0.314	0.323
**Model 8**				
lnL	-4898.959	-1983.569	-2308.308	-10892.589
p	7.762	0.237	0.323	0.453
q	24.354	0.278	0.658	0.911
p_0	0.677	0.901	0.979	0.953
d_N/d_S (p_1)	1.427(0.323)	4.016(0.099)	3.1(0.021)	2.726(0.047)
2ΔlnL(LRT) (8vs7)	3.89	29.108	2.722	35.51
P Value	**0.048**	**6.85E-08**	0.099	**2.54E-09**
Postitively selected sites*	300T	36A,44L,127V,130K,131V	159P	136Y,237Q,251P,480N,613Q,615I,713Q,803R,841R

*:BEB>0.9

Table S5 HBV genome mutations in experimental group and control group.

Experimental group

Patients	Ref Pos	Ref	Clone count	Mutant count A	Mutant count C	Mutant count G	Mutant count T	Deletion
D10	2	T	13	0	1	0	0	0
D10	44	G	13	0	1	0	0	0
D10	51	A	13	0	0	1	0	0
D10	87	G	13	0	13	0	0	0
D10	113	A	13	0	0	1	0	0
D10	254	T	13	0	2	0	0	0
D10	286	A	13	0	0	13	0	0
D10	401	T	13	0	1	0	0	0
D10	453	A	13	0	0	4	0	0
D10	475	T	13	0	1	0	0	0
D10	592	C	13	0	0	0	13	0
D10	685	G	13	4	0	0	0	0
D10	793	G	13	13	0	0	0	0
D10	796	G	13	0	2	0	0	0
D10	835	A	13	0	0	1	0	0
D10	836	A	13	0	9	0	0	0
D10	849	A	13	0	0	0	13	0
D10	903	C	13	4	0	0	0	0
D10	915	A	13	0	0	13	0	0
D10	987	A	13	0	12	0	1	0
D10	988	T	13	0	2	0	0	0
D10	996	A	13	0	9	0	0	0
D10	1009	T	13	0	13	0	0	0
D10	1074	T	13	13	0	0	0	0
D10	1171	C	13	0	0	0	4	0
D10	1218	C	13	13	0	0	0	0
D10	1269	T	13	0	1	0	0	0
D10	1318	A	13	0	1	0	0	0
D10	1320	A	13	0	1	0	0	0
D10	1365	A	13	0	1	0	0	0
D10	1368	A	13	0	13	0	0	0
D10	1484	A	13	0	0	1	0	0
D10	1503	G	13	13	0	0	0	0
D10	1504	T	13	0	9	0	0	0
D10	1613	G	13	13	0	0	0	0
D10	1640	G	13	0	1	0	0	0
D10	1672	T	13	0	1	0	0	0
D10	1676	A	13	0	4	0	0	0
D10	1727	T	13	0	0	13	0	0
D10	1762	A	13	0	0	0	13	0
D10	1764	G	13	13	0	0	0	0
D10	1802	T	13	0	13	0	0	0
D10	1846	A	13	0	0	0	13	0
D10	1896	G	13	2	0	0	0	0
D10	1969	T	13	0	13	0	0	0
D10	1993	C	13	0	0	0	1	0
D10	2095	A	13	0	0	13	0	0
D10	2131	A	13	0	0	0	9	0
D10	2151	T	13	0	4	0	0	0
D10	2159	A	13	0	0	13	0	0
D10	2183	T	13	0	13	0	0	0
D10	2189	A	13	0	13	0	0	0
D10	2234	A	13	0	0	1	0	0
D10	2235	G	13	2	0	0	0	0
D10	2244	T	13	0	1	0	0	0
D10	2288	C	13	0	0	0	13	0
D10	2303	C	13	0	0	0	2	0
D10	2525	A	13	0	0	0	13	0
D10	2541	T	13	1	0	0	0	0
D10	2661	G	13	13	0	0	0	0
D10	2712	T	13	13	0	0	0	0
D10	2738	A	13	0	0	13	0	0
D10	2789	G	13	13	0	0	0	0
D10	2805	T	13	0	1	0	0	0
D10	2832	G	13	1	0	0	0	0
D10	2993	A	13	0	0	1	0	0
D10	3059	-	13	0	1	0	0	12
D10	3061	G	13	0	0	0	0	1
D10	3163	A	13	0	0	2	0	0
D10	3185	G	13	2	0	0	0	0
D10	3205	A	13	0	0	4	0	0
D07	1	C	15	0	0	0	1	0
D07	6	C	15	0	0	15	0	0
D07	7	A	15	0	15	0	0	0
D07	26	C	15	0	0	0	1	0
D07	31	T	15	0	2	0	0	0
D07	33	C	15	1	0	0	0	0
D07	54	T	15	0	0	2	0	0
D07	64	T	15	0	1	0	0	0
D07	70	C	15	15	0	0	0	0
D07	126	T	15	0	1	0	0	0
D07	149	G	15	0	15	0	0	0
D07	154	C	15	0	0	0	13	0
D07	162	A	15	0	0	13	0	0
D07	166	A	15	0	15	0	0	0
D07	167	A	15	0	0	1	0	0
D07	182	G	15	2	0	0	0	0
D07	211	T	15	0	1	0	0	0
D07	213	T	15	0	2	0	0	0
D07	216	T	15	0	1	0	0	0
D07	220	G	15	1	0	0	0	0
D07	225	G	15	2	0	0	0	0
D07	288	C	15	0	0	0	2	0
D07	312	T	15	0	2	0	0	0
D07	336	C	15	0	0	0	5	0
D07	339	C	15	0	0	0	10	0
D07	340	A	15	0	0	1	0	0
D07	342	C	15	0	0	0	2	0
D07	357	C	15	0	0	0	15	0
D07	367	C	15	0	0	0	15	0
D07	373	C	15	0	0	0	15	0
D07	374	T	15	0	1	0	0	0
D07	400	A	15	0	13	0	0	0
D07	414	T	15	0	1	0	0	0
D07	438	T	15	0	0	2	0	0
D07	446	C	15	0	0	1	0	0
D07	493	T	15	15	0	0	0	0
D07	524	T	15	1	0	0	0	0
D07	529	G	15	0	0	0	1	0
D07	530	A	15	0	0	3	0	0
D07	531	T	15	0	0	1	0	0
D07	532	A	15	0	0	0	15	0
D07	562	T	15	2	0	0	0	0

Control group

Patients	Ref Pos	Ref	Clone count	Mutant count A	Mutant count C	Mutant count G	Mutant count T	Deletion
LYL	44	G	12	0	1	0	0	0
LYL	66	G	12	0	1	0	0	0
LYL	172	A	12	0	0	1	0	0
LYL	218	T	12	0	3	0	0	0
LYL	272	A	12	0	0	1	0	0
LYL	283	G	12	1	0	0	0	0
LYL	287	A	12	0	0	1	0	0
LYL	333	A	12	0	0	1	0	0
LYL	481	A	12	0	12	0	0	0
LYL	893	G	12	1	0	0	0	0
LYL	914	A	12	0	0	1	0	0
LYL	915	A	12	0	0	12	0	0
LYL	963	A	12	0	0	0	1	0
LYL	967	A	12	0	12	0	0	0
LYL	996	A	12	0	5	0	0	0
LYL	1002	G	12	12	0	0	0	0
LYL	1074	T	12	12	0	0	0	0
LYL	1111	A	12	0	0	3	0	0
LYL	1182	G	12	5	0	0	0	0
LYL	1221	A	12	0	0	0	12	0
LYL	1306	A	12	0	1	0	0	0
LYL	1320	A	12	0	12	0	0	0
LYL	1350	C	12	0	0	0	6	0
LYL	1358	A	12	0	0	1	0	0
LYL	1425	T	12	0	0	5	0	0
LYL	1503	G	12	0	12	0	0	0
LYL	1506	G	12	0	12	0	0	0
LYL	1512	A	12	0	0	0	3	0
LYL	1613	G	12	6	0	0	0	0
LYL	1731	T	12	0	1	0	0	0
LYL	1747	A	12	0	1	0	0	0
LYL	1961	T	12	0	3	0	0	0
LYL	1969	T	12	0	11	0	0	0
LYL	1972	T	12	0	3	0	0	0
LYL	1978	T	12	0	3	0	0	0
LYL	1986	A	12	0	0	1	0	0
LYL	1993	C	12	0	0	0	1	0
LYL	2006	G	12	3	0	0	0	0
LYL	2017	G	12	1	0	0	0	0
LYL	2059	G	12	1	0	0	0	0
LYL	2062	A	12	0	0	0	11	0
LYL	2095	A	12	0	0	12	0	0
LYL	2183	T	12	0	12	0	0	0
LYL	2266	T	12	0	1	0	0	0
LYL	2437	G	12	1	0	0	0	0
LYL	2480	C	12	0	0	0	12	0
LYL	2489	G	12	1	0	0	0	0
LYL	2523	A	12	0	12	0	0	0
LYL	2643	G	12	5	0	0	0	0
LYL	2660	T	12	0	0	1	0	0
LYL	2720	T	12	0	1	0	0	0
LYL	2738	A	12	0	0	12	0	0
LYL	2901	C	12	0	0	0	1	0
LYL	3000	C	12	12	0	0	0	0
LYL	3087	G	12	0	1	0	0	0
LYL	3096	C	12	0	0	0	1	0
LYL	3185	G	12	1	0	0	0	0
CFO	21	C	14	0	0	0	1	0
CFO	26	C	14	1	0	0	0	0
CFO	52	C	14	0	0	0	14	0
CFO	90	C	14	0	0	0	1	0
CFO	109	T	14	14	0	0	0	0
CFO	149	G	14	0	14	0	0	0
CFO	153	A	14	0	0	1	0	0
CFO	197	T	14	0	1	0	0	0
CFO	254	T	14	0	1	0	0	0
CFO	293	A	14	0	0	3	0	0
CFO	312	T	14	0	14	0	0	0
CFO	336	C	14	0	0	0	6	0
CFO	337	A	14	0	0	1	0	0
CFO	339	C	14	0	0	0	1	0
CFO	357	C	14	0	0	0	14	0
CFO	411	T	14	0	1	0	0	0
CFO	480	T	14	0	4	0	0	0
CFO	483	T	14	0	1	0	0	0
CFO	493	T	14	14	0	0	0	0
CFO	504	G	14	1	0	0	0	0
CFO	532	A	14	0	0	0	14	0
CFO	552	T	14	0	1	0	0	0
CFO	592	T	14	0	14	0	0	0
CFO	631	A	14	0	0	1	0	0
CFO	735	C	14	0	0	0	14	0
CFO	782	A	14	0	0	1	0	0
CFO	820	C	14	0	0	0	14	0
CFO	834	A	14	0	0	13	0	0
CFO	840	C	14	0	0	0	14	0
CFO	843	C	14	0	0	0	14	0
CFO	847	A	14	0	1	0	0	0
CFO	867	T	14	0	14	0	0	0
CFO	876	T	14	0	14	0	0	0
CFO	882	C	14	14	0	0	0	0
CFO	906	A	14	0	0	0	14	0
CFO	929	T	14	14	0	0	0	0
CFO	930	A	14	0	14	0	0	0
CFO	934	A	14	0	12	0	0	0
CFO	941	C	14	14	0	0	0	0
CFO	951	G	14	14	0	0	0	0
CFO	955	C	14	0	0	0	14	0
CFO	969	C	14	14	0	0	0	0
CFO	984	A	14	0	0	3	0	0
CFO	988	T	14	0	3	0	0	0
CFO	1003	T	14	0	0	14	0	0
CFO	1007	T	14	0	1	0	0	0
CFO	1008	T	14	0	1	0	0	0
CFO	1014	T	14	0	14	0	0	0
CFO	1020	C	14	0	0	0	13	0
CFO	1023	T	14	0	14	0	0	0
CFO	1026	C	14	0	0	0	14	0
CFO	1047	G	14	0	0	0	14	0
CFO	1050	A	14	0	14	0	0	0
CFO	1053	A	14	0	0	14	0	0
CFO	1062	G	14	14	0	0	0	0
CFO	1078	G	14	0	0	0	14	0

D07	619	C	15	0	0	0	15	0	CFQ	1083	G	14	3	0	0	0	0
D07	620	T	15	1	0	0	0	0	CFQ	1101	G	14	1	0	0	0	0
D07	645	A	15	0	0	1	0	0	CFQ	1158	G	14	1	0	0	0	0
D07	658	C	15	1	0	0	0	0	CFQ	1191	C	14	0	0	0	1	0
D07	735	C	15	0	0	0	15	0	CFQ	1206	C	14	14	0	0	0	0
D07	744	T	15	0	1	0	0	0	CFQ	1218	A	14	0	14	0	0	0
D07	768	T	15	0	1	0	0	0	CFQ	1219	C	14	14	0	0	0	0
D07	771	A	15	0	0	1	0	0	CFQ	1228	A	14	0	14	0	0	0
D07	774	A	15	0	1	0	0	0	CFQ	1229	A	14	0	0	14	0	0
D07	777	T	15	0	1	0	0	0	CFQ	1234	C	14	14	0	0	0	0
D07	783	G	15	15	0	0	0	0	CFQ	1317	-	14	0	0	14	0	0
D07	791	T	15	1	0	0	0	0	CFQ	1321	A	14	0	0	0	0	14
D07	796	T	15	0	0	15	0	0	CFQ	1322	T	14	0	1	0	0	0
D07	799	A	15	0	0	2	0	0	CFQ	1323	C	14	0	0	0	14	0
D07	820	C	15	0	0	0	15	0	CFQ	1329	A	14	0	14	0	0	0
D07	837	C	15	0	0	0	1	0	CFQ	1332	T	14	0	14	0	0	0
D07	840	C	15	0	0	0	14	0	CFQ	1341	C	14	0	0	0	14	0
D07	841	A	15	0	1	0	0	0	CFQ	1347	T	14	0	14	0	0	0
D07	843	C	15	0	0	0	15	0	CFQ	1356	T	14	0	0	14	0	0
D07	867	T	15	0	15	0	0	0	CFQ	1368	C	14	14	0	0	0	0
D07	872	A	15	0	0	1	0	0	CFQ	1386	C	14	0	0	14	0	0
D07	876	T	15	0	15	0	0	0	CFQ	1399	T	14	0	1	0	0	0
D07	882	C	15	15	0	0	0	0	CFQ	1407	G	14	1	0	0	0	0
D07	896	G	15	1	0	0	0	0	CFQ	1421	T	14	0	1	0	0	0
D07	906	A	15	0	15	0	0	0	CFQ	1436	C	14	0	0	0	1	0
D07	923	T	15	1	0	0	0	0	CFQ	1458	T	14	0	14	0	0	0
D07	929	T	15	15	0	0	0	0	CFQ	1477	G	14	1	0	0	0	0
D07	930	A	15	0	0	0	1	0	CFQ	1479	G	14	0	0	0	14	0
D07	934	A	15	0	0	0	15	0	CFQ	1480	G	14	0	14	0	0	0
D07	936	C	15	0	0	15	0	0	CFQ	1485	C	14	0	0	0	1	0
D07	939	G	15	15	0	0	0	0	CFQ	1498	T	14	0	14	0	0	0
D07	941	C	15	15	0	0	0	0	CFQ	1499	G	14	11	0	0	0	0
D07	944	G	15	1	0	0	0	0	CFQ	1515	A	14	0	0	14	0	0
D07	957	G	15	0	0	0	15	0	CFQ	1526	G	14	1	0	0	0	0
D07	960	T	15	0	1	0	0	0	CFQ	1554	T	14	0	1	0	0	0
D07	961	G	15	15	0	0	0	0	CFQ	1613	A	14	0	0	4	0	0
D07	962	T	15	0	1	0	0	0	CFQ	1624	A	14	0	0	1	0	0
D07	965	A	15	0	1	0	0	0	CFQ	1637	A	14	0	14	0	0	0
D07	969	C	15	15	0	0	0	0	CFQ	1638	T	14	1	0	0	0	0
D07	1003	T	15	0	0	15	0	0	CFQ	1653	T	14	0	14	0	0	0
D07	1009	T	15	0	15	0	0	0	CFQ	1655	T	14	0	1	0	0	0
D07	1014	T	15	0	15	0	0	0	CFQ	1658	G	14	0	14	0	0	0
D07	1023	T	15	0	15	0	0	0	CFQ	1679	G	14	12	0	0	0	0
D07	1026	C	15	0	0	0	15	0	CFQ	1685	A	14	0	0	1	0	0
D07	1032	A	15	0	0	0	15	0	CFQ	1721	A	14	0	0	14	0	0
D07	1044	T	15	0	15	0	0	0	CFQ	1731	T	14	0	1	0	0	0
D07	1047	G	15	15	0	0	0	0	CFQ	1740	T	14	0	1	0	0	0
D07	1062	G	15	15	0	0	0	0	CFQ	1762	A	14	0	0	0	2	0
D07	1081	A	15	0	0	1	0	0	CFQ	1763	G	14	5	0	0	0	0
D07	1082	A	15	0	0	15	0	0	CFQ	1764	A	14	0	0	6	0	0
D07	1120	C	15	0	0	0	15	0	CFQ	1765	T	14	0	5	0	0	0
D07	1130	A	15	0	0	0	1	0	CFQ	1766	T	14	0	13	0	0	0
D07	1155	C	15	0	0	0	15	0	CFQ	1768	A	14	0	0	0	13	0
D07	1173	C	15	0	0	0	15	0	CFQ	1846	T	14	12	0	0	0	0
D07	1191	C	15	0	0	0	15	0	CFQ	1871	T	14	0	1	0	0	0
D07	1199	C	15	1	0	0	0	0	CFQ	1896	A	14	0	0	14	0	0
D07	1203	T	15	0	0	1	0	0	CFQ	1913	A	14	0	12	1	0	0
D07	1206	C	15	0	0	0	14	0	CFQ	1915	G	14	14	0	0	0	0
D07	1218	A	15	0	15	0	0	0	CFQ	1922	C	14	0	0	14	0	0
D07	1219	C	15	15	0	0	0	0	CFQ	1923	C	14	14	0	0	0	0
D07	1221	A	15	0	0	11	0	0	CFQ	1928	G	14	1	0	0	0	0
D07	1228	A	15	0	15	0	0	0	CFQ	1933	A	14	0	0	0	14	0
D07	1229	A	15	0	0	1	0	0	CFQ	1961	T	14	0	0	1	0	0
D07	1234	C	15	15	0	0	0	0	CFQ	1969	C	14	0	0	0	1	0
D07	1317	-	15	0	0	15	0	0	CFQ	1979	G	14	13	0	0	0	0
D07	1321	A	15	0	0	0	0	15	CFQ	1984	T	14	14	0	0	0	0
D07	1323	C	15	0	0	0	15	0	CFQ	2011	G	14	1	0	0	0	0
D07	1329	A	15	0	15	0	0	0	CFQ	2015	C	14	0	0	0	1	0
D07	1332	T	15	0	15	0	0	0	CFQ	2078	G	14	0	14	0	0	0
D07	1341	C	15	0	0	1	14	0	CFQ	2107	A	14	0	11	0	1	0
D07	1347	T	15	0	15	0	0	0	CFQ	2134	T	14	0	11	0	0	0
D07	1351	T	15	0	1	0	0	0	CFQ	2149	A	14	0	0	0	2	0
D07	1356	T	15	0	0	15	0	0	CFQ	2158	A	14	0	10	0	0	0
D07	1365	C	15	1	0	0	14	0	CFQ	2159	A	14	0	0	2	0	0
D07	1368	C	15	2	0	0	0	0	CFQ	2189	A	14	0	2	0	0	0
D07	1409	G	15	1	0	0	0	0	CFQ	2197	A	14	0	0	1	0	0
D07	1411	G	15	1	0	0	0	0	CFQ	2209	C	14	0	0	0	10	0
D07	1417	T	15	0	1	0	0	0	CFQ	2227	T	14	0	1	0	0	0
D07	1440	G	15	1	0	0	0	0	CFQ	2242	T	14	0	14	0	0	0
D07	1458	T	15	0	15	0	0	0	CFQ	2243	G	14	1	0	0	0	0
D07	1464	T	15	0	1	0	0	0	CFQ	2259	T	14	0	3	0	0	0
D07	1479	G	15	13	0	0	0	0	CFQ	2288	C	14	1	0	0	0	0
D07	1480	G	15	0	15	0	0	0	CFQ	2304	C	14	1	0	0	0	0
D07	1481	T	15	0	1	0	0	0	CFQ	2330	C	14	1	0	0	0	0
D07	1498	T	15	0	15	0	0	0	CFQ	2357	G	14	0	0	0	2	0
D07	1504	C	15	1	0	0	0	0	CFQ	2401	G	14	14	0	0	0	0
D07	1515	A	15	0	0	15	0	0	CFQ	2410	A	14	0	0	14	0	0
D07	1535	T	15	0	1	0	0	0	CFQ	2423	A	14	0	0	1	0	0
D07	1580	T	15	0	1	0	0	0	CFQ	2444	C	14	0	0	0	2	0
D07	1613	A	15	0	0	6	0	0	CFQ	2459	T	14	0	14	0	0	0
D07	1624	A	15	0	1	0	0	0	CFQ	2501	T	14	1	0	0	0	0
D07	1637	A	15	0	15	0	0	0	CFQ	2537	T	14	0	14	0	0	0
D07	1653	T	15	0	15	0	0	0	CFQ	2558	G	14	14	0	0	0	0
D07	1673	C	15	0	0	0	1	0	CFQ	2560	G	14	14	0	0	0	0
D07	1679	G	15	15	0	0	0	0	CFQ	2564	A	14	0	0	14	0	0
D07	1721	A	15	0	0	15	0	0	CFQ	2567	C	14	0	0	0	14	0
D07	1750	A	15	0	0	1	0	0	CFQ	2573	C	14	0	0	0	14	0
D07	1753	T	15	0	15	0	0	0	CFQ	2579	A	14	0	0	1	0	0
D07	1755	A	15	0	15	0	0	0	CFQ	2582	C	14	0	0	0	14	0
D07	1762	A	15	0	0	0	15	0	CFQ	2588	A	14	0	0	1	0	0
D07	1766	T	15	0	15	0	0	0	CFQ	2591	C	14	0	0	0	14	0
D07	1768	A	15	0	0	0	15	0	CFQ	2598	C	14	0	0	0	7	0
D07	1775	A	15	0	0	15	0	0	CFQ	2603	C	14	0	0	0	14	0
D07	1779	G	15	1	0	0	0	0	CFQ	2609	T	14	0	0	3	0	0
D07	1826	C	15	0	0	0	1	0	CFQ	2612	C	14	0	0	0	14	0
D07	1846	T	15	15	0	0	0	0	CFQ	2635	T	14	0	1	0	0	0
D07	1896	A	15	0	0	15	0	0	CFQ	2652	T	14	0	1	0	0	0
D07	1899	G	15	3	0	0	0	0	CFQ	2662	T	14	0	0	3	0	0
D07	1913	A	15	0	15	0	0	0	CFQ	2681	A	14	0	0	14	0	0
D07	1922	C	15	0	0	15	0	0	CFQ	2684	T	14	0	14	0	0	0
D07	1923	C	15	15	0	0	0	0	CFQ	2699	T	14	0	0	14	0	0
D07	1932	C	15	0	0	0	1	0	CFQ	2712	C	14	0	0	0	14	0
D07	1933	A	15	0	0	0	15	0	CFQ	2721	A	14	0	0	2	0	0
D07	1939	G	15	1	0	0	0	0	CFQ	2731	T	14	0	1	0	0	0
D07	1979	G	15	15	0	0	0	0	CFQ	2732	T	14	0	14	0	0	0
D07	1984	T	15	15	0	0	0	0	CFQ	2733	A	14	0	14	0	0	0
D07	1993	C	15	0	0	0	2	0	CFQ	2848	A	14	0	0	1	0	0
D07	2009	C	15	0	0	0	15	0	CFQ	2859	G	14	1	0	0	0	0
D07	2065	C	15	15	0	0	0	0	CFQ	2871	T	14	0	1	0	0	0
D07	2066	A	15	0	0	1	0	0	CFQ	2875	A	14	0	14	0	0	0
D07	2068	G	15	1	0	0	0	0	CFQ	2894	T	14	0	1	0	0	0
D07	2075	A	15	0	0	0	1	0	CFQ	2898	G	14	0	0	0	14	0

D07	2078	G	15	0	15	0	0	0
D07	2091	A	15	0	0	0	1	0
D07	2107	A	15	0	15	0	0	0
D07	2120	A	15	0	0	1	0	0
D07	2134	T	15	0	15	0	0	0
D07	2159	A	15	0	0	1	0	0
D07	2168	A	15	0	0	1	0	0
D07	2170	T	15	0	4	0	0	0
D07	2188	A	15	0	0	0	2	0
D07	2189	A	15	0	13	0	0	0
D07	2196	A	15	0	0	1	0	0
D07	2198	C	15	12	0	0	0	0
D07	2209	C	15	0	0	0	15	0
D07	2221	T	15	0	2	0	0	0
D07	2224	T	15	1	0	0	0	0
D07	2248	T	15	15	0	0	0	0
D07	2260	G	15	1	0	0	0	0
D07	2282	A	15	0	0	1	0	0
D07	2288	C	15	12	0	0	2	0
D07	2290	C	15	1	0	0	0	0
D07	2299	A	15	0	0	1	0	0
D07	2335	A	15	0	0	1	0	0
D07	2363	T	15	2	0	0	0	0
D07	2410	A	15	0	0	15	0	0
D07	2504	T	15	0	1	0	0	0
D07	2507	A	15	0	0	1	0	0
D07	2525	G	15	0	15	0	0	0
D07	2537	T	15	0	15	0	0	0
D07	2540	C	15	0	0	0	15	0
D07	2546	T	15	0	1	0	0	0
D07	2549	C	15	15	0	0	0	0
D07	2559	C	15	15	0	0	0	0
D07	2560	G	15	15	0	0	0	0
D07	2564	A	15	0	0	15	0	0
D07	2575	A	15	0	15	0	0	0
D07	2576	T	15	0	15	0	0	0
D07	2590	A	15	0	0	0	14	0
D07	2591	C	15	0	0	0	15	0
D07	2597	C	15	0	0	0	1	0
D07	2603	C	15	0	0	0	15	0
D07	2626	T	15	0	1	0	0	0
D07	2633	A	15	0	0	15	0	0
D07	2651	C	15	0	0	0	1	0
D07	2660	C	15	0	0	0	1	0
D07	2666	C	15	0	0	0	15	0
D07	2681	A	15	0	0	1	0	0
D07	2715	G	15	15	0	0	0	0
D07	2732	T	15	0	15	0	0	0
D07	2733	A	15	0	15	0	0	0
D07	2741	A	15	0	0	15	0	0
D07	2743	A	15	0	0	1	0	0
D07	2766	G	15	1	0	0	0	0
D07	2768	T	15	0	0	15	0	0
D07	2771	C	15	0	0	0	15	0
D07	2772	A	15	0	0	1	0	0
D07	2775	C	15	0	0	0	15	0
D07	2812	G	15	5	0	0	0	0
D07	2860	T	15	1	0	0	0	0
D07	2875	A	15	0	15	0	0	0
D07	2892	T	15	0	15	0	0	0
D07	2898	G	15	0	0	0	15	0
D07	2926	G	15	1	0	0	0	0
D07	2927	A	15	0	0	15	0	0
D07	2943	T	15	0	0	15	0	0
D07	2946	G	15	12	0	0	0	0
D07	2955	C	15	1	0	0	0	0
D07	2969	C	15	0	0	0	1	0
D07	2996	A	15	0	0	2	0	0
D07	3000	C	15	15	0	0	0	0
D07	3015	T	15	0	15	0	0	0
D07	3016	C	15	1	0	0	0	0
D07	3022	G	15	0	0	0	2	0
D07	3026	C	15	0	0	0	15	0
D07	3030	A	15	0	0	0	1	0
D07	3031	G	15	0	0	0	15	0
D07	3039	G	15	0	0	0	1	0
D07	3046	T	15	0	1	0	0	0
D07	3057	C	15	15	0	0	0	0
D07	3062	G	15	1	0	0	0	0
D07	3064	A	15	0	0	1	0	0
D07	3075	G	15	0	0	0	2	0
D07	3084	G	15	0	0	0	15	0
D07	3105	T	15	15	0	0	0	0
D07	3109	G	15	2	0	0	0	0
D07	3121	C	15	1	0	0	0	0
D07	3122	C	15	0	0	0	1	0
D07	3140	A	15	0	2	0	0	0
D07	3151	G	15	2	0	0	0	0
D07	3153	A	15	0	0	2	0	0
D07	3162	T	15	0	1	0	0	0
D07	3165	T	15	0	1	0	0	0
D07	3171	C	15	1	0	0	0	0
D07	3188	A	15	0	0	1	0	0
D07	3192	T	15	0	1	0	0	0
D07	3204	T	15	0	15	0	0	0
D07	3209	A	15	0	0	2	0	0
D15	10	A	14	0	14	0	0	0
D15	15	A	14	0	0	0	14	0
D15	18	A	14	0	0	1	0	0
D15	20	G	14	13	0	0	0	0
D15	27	T	14	14	0	0	0	0
D15	37	A	14	0	0	13	0	0
D15	41	A	14	0	0	0	4	0
D15	43	G	14	14	0	0	0	0
D15	49	A	14	0	0	14	0	0
D15	52	C	14	0	0	0	14	0
D15	55	T	14	0	14	0	0	0
D15	76	C	14	14	0	0	0	0
D15	105	C	14	0	0	0	7	0
D15	126	T	14	0	13	0	0	0
D15	135	C	14	0	0	0	13	0
D15	148	A	14	0	0	0	0	14
D15	151	-	14	0	0	0	14	0
D15	163	C	14	1	0	0	0	0
D15	165	C	14	0	0	0	14	0
D15	166	A	14	0	14	0	0	0
D15	182	G	14	14	0	0	0	0
D15	183	G	14	5	9	0	0	0
D15	195	T	14	0	14	0	0	0
D15	216	T	14	0	1	4	0	0
D15	241	A	14	0	0	14	0	0

CFQ	2937	G	14	1	0	0	0	0
CFQ	2958	T	14	0	14	0	0	0
CFQ	2984	A	14	0	0	1	0	0
CFQ	3008	C	14	14	0	0	0	0
CFQ	3039	G	14	0	0	0	14	0
CFQ	3051	T	14	0	14	0	0	0
CFQ	3057	C	14	14	0	0	0	0
CFQ	3064	A	14	0	0	14	0	0
CFQ	3084	G	14	0	0	0	14	0
CFQ	3105	T	14	14	0	0	0	0
CFQ	3115	G	14	1	0	0	0	0
CFQ	3120	G	14	14	0	0	0	0
CFQ	3204	T	14	0	14	0	0	0
CFQ	3208	C	14	0	0	0	1	0
KXY	6	C	18	0	0	18	0	0
KXY	7	A	18	0	18	0	0	0
KXY	35	A	18	0	1	0	0	0
KXY	48	T	18	0	1	0	0	0
KXY	70	C	18	18	0	0	0	0
KXY	79	A	18	0	0	1	0	0
KXY	113	A	18	0	0	18	0	0
KXY	119	T	18	0	1	0	0	0
KXY	149	G	18	0	18	0	0	0
KXY	153	A	18	0	0	3	0	0
KXY	154	C	18	0	0	0	17	0
KXY	157	G	18	1	0	0	0	0
KXY	162	A	18	0	0	18	0	0
KXY	166	A	18	0	18	0	0	0
KXY	223	A	18	0	0	0	1	0
KXY	300	C	18	0	0	0	1	0
KXY	357	C	18	0	0	0	18	0
KXY	367	C	18	0	0	0	18	0
KXY	400	A	18	0	18	0	0	0
KXY	454	C	18	0	0	0	18	0
KXY	491	A	18	0	3	0	0	0
KXY	493	T	18	17	0	1	0	0
KXY	511	A	18	0	0	18	0	0
KXY	532	A	18	0	0	0	18	0
KXY	540	A	18	0	0	2	0	0
KXY	575	A	18	0	0	1	0	0
KXY	619	C	18	0	0	0	18	0
KXY	657	T	18	0	0	1	0	0
KXY	673	C	18	0	0	0	1	0
KXY	684	T	18	0	3	0	0	0
KXY	710	C	18	0	0	0	1	0
KXY	729	T	18	0	1	0	0	0
KXY	734	G	18	0	1	0	0	0
KXY	735	C	18	0	0	0	18	0
KXY	765	G	18	1	0	0	0	0
KXY	783	G	18	18	0	0	0	0
KXY	820	C	18	0	0	0	18	0
KXY	840	C	18	0	0	0	18	0
KXY	843	C	18	0	0	0	18	0
KXY	867	T	18	0	18	0	0	0
KXY	876	T	18	0	18	0	0	0
KXY	881	G	18	1	0	0	0	0
KXY	906	A	18	0	18	0	0	0
KXY	929	T	18	18	0	0	0	0
KXY	934	A	18	0	0	0	18	0
KXY	936	C	18	0	0	18	0	0
KXY	939	G	18	18	0	0	0	0
KXY	941	C	18	18	0	0	0	0
KXY	957	G	18	0	0	0	18	0
KXY	961	G	18	18	0	0	0	0
KXY	969	C	18	18	0	0	0	0
KXY	995	G	18	1	0	0	0	0
KXY	1003	T	18	0	0	18	0	0
KXY	1009	T	18	0	18	0	0	0
KXY	1014	T	18	0	18	0	0	0
KXY	1015	T	18	0	1	0	0	0
KXY	1023	T	18	0	18	0	0	0
KXY	1026	C	18	0	0	0	18	0
KXY	1044	T	18	0	18	0	0	0
KXY	1047	G	18	18	0	0	0	0
KXY	1050	A	18	0	18	0	0	0
KXY	1062	G	18	18	0	0	0	0
KXY	1083	G	18	18	0	0	0	0
KXY	1155	C	18	0	0	0	18	0
KXY	1158	G	18	18	0	0	0	0
KXY	1173	C	18	0	0	0	18	0
KXY	1206	C	18	0	0	0	18	0
KXY	1218	A	18	0	0	0	18	0
KXY	1219	C	18	18	0	0	0	0
KXY	1228	A	18	0	18	0	0	0
KXY	1234	C	18	18	0	0	0	0
KXY	1278	A	18	0	1	0	0	0
KXY	1301	G	18	3	0	0	0	0
KXY	1307	G	18	2	0	0	0	0
KXY	1317	-	18	0	3	10	5	0
KXY	1321	A	18	0	0	0	0	18
KXY	1323	C	18	0	0	0	18	0
KXY	1329	A	18	0	18	0	0	0
KXY	1332	T	18	0	18	0	0	0
KXY	1341	C	18	0	0	0	18	0
KXY	1347	T	18	0	18	0	0	0
KXY	1356	T	18	0	0	18	0	0
KXY	1382	T	18	0	3	0	0	0
KXY	1387	T	18	0	1	0	0	0
KXY	1396	A	18	0	0	1	0	0
KXY	1458	T	18	0	18	0	0	0
KXY	1461	G	18	18	0	0	0	0
KXY	1480	G	18	0	18	0	0	0
KXY	1498	T	18	0	18	0	0	0
KXY	1515	A	18	0	0	18	0	0
KXY	1548	C	18	0	0	0	2	0
KXY	1613	A	18	0	0	5	0	0
KXY	1637	A	18	0	18	0	0	0
KXY	1653	T	18	0	18	0	0	0
KXY	1679	G	18	18	0	0	0	0
KXY	1702	C	18	0	0	0	1	0
KXY	1741	T	18	0	3	0	0	0
KXY	1747	A	18	0	0	1	0	0
KXY	1764	A	18	0	0	2	0	0
KXY	1766	T	18	0	2	0	0	0
KXY	1768	A	18	0	0	0	2	0
KXY	1775	A	18	0	0	5	0	0
KXY	1778	A	18	0	0	3	0	0
KXY	1792	A	18	0	0	1	0	0
KXY	1846	T	18	18	0	0	0	0
KXY	1896	A	18	0	0	18	0	0
KXY	1913	A	18	0	18	0	0	0

D15	273	A	14	0	0	1	0	0
D15	287	G	14	14	0	0	0	0
D15	289	A	14	0	0	0	14	0
D15	290	C	14	14	0	0	0	0
D15	293	A	14	0	0	14	0	0
D15	294	C	14	0	0	0	14	0
D15	300	C	14	0	0	14	0	0
D15	312	T	14	0	14	0	0	0
D15	346	T	14	0	14	0	0	0
D15	367	C	14	0	0	0	14	0
D15	400	A	14	0	14	0	0	0
D15	454	C	14	0	0	0	14	0
D15	482	C	14	9	0	0	0	0
D15	491	A	14	0	0	0	9	0
D15	492	C	14	5	0	0	0	0
D15	496	A	14	0	0	14	0	0
D15	499	T	14	0	14	0	0	0
D15	512	C	14	0	0	0	1	0
D15	514	A	14	0	13	0	0	0
D15	519	A	14	0	0	14	0	0
D15	520	G	14	14	0	0	0	0
D15	531	T	14	0	14	0	0	0
D15	532	A	14	0	0	0	14	0
D15	542	G	14	14	0	0	0	0
D15	543	G	14	9	0	0	0	0
D15	544	A	14	0	9	0	0	0
D15	546	C	14	1	0	0	1	0
D15	552	T	14	0	5	0	0	0
D15	555	T	14	14	0	0	0	0
D15	562	T	14	0	14	0	0	0
D15	585	A	14	0	3	0	6	0
D15	598	T	14	0	14	0	0	0
D15	628	C	14	1	0	0	0	0
D15	630	C	14	0	0	14	0	0
D15	633	G	14	14	0	0	0	0
D15	657	T	14	0	14	0	0	0
D15	667	C	14	0	0	0	14	0
D15	672	T	14	0	1	0	0	0
D15	735	C	14	0	0	0	14	0
D15	762	C	14	4	0	0	0	0
D15	766	T	14	14	0	0	0	0
D15	774	A	14	0	0	14	0	0
D15	777	T	14	0	14	0	0	0
D15	794	C	14	1	0	0	0	0
D15	796	T	14	0	0	14	0	0
D15	799	A	14	0	0	14	0	0
D15	801	T	14	0	4	0	0	0
D15	804	C	14	0	0	0	4	0
D15	813	T	14	1	0	0	0	0
D15	820	C	14	0	0	0	14	0
D15	825	T	14	0	4	0	0	0
D15	834	A	14	0	0	14	0	0
D15	840	C	14	0	0	0	14	0
D15	843	C	14	0	0	0	14	0
D15	867	T	14	0	14	0	0	0
D15	876	T	14	0	14	0	0	0
D15	882	C	14	14	0	0	0	0
D15	886	G	14	14	0	0	0	0
D15	887	T	14	0	0	14	0	0
D15	888	A	14	0	0	4	0	0
D15	906	A	14	0	0	0	14	0
D15	909	G	14	14	0	0	0	0
D15	918	A	14	0	14	0	0	0
D15	927	A	14	0	0	1	0	0
D15	929	T	14	14	0	0	0	0
D15	930	A	14	0	9	0	0	0
D15	941	C	14	14	0	0	0	0
D15	955	C	14	0	0	0	14	0
D15	963	A	14	0	1	0	4	0
D15	969	C	14	14	0	0	0	0
D15	973	A	14	0	0	14	0	0
D15	977	A	14	0	0	6	0	0
D15	1003	T	14	0	0	14	0	0
D15	1014	T	14	0	14	0	0	0
D15	1019	C	14	0	0	0	6	0
D15	1020	C	14	0	0	0	14	0
D15	1023	T	14	0	14	0	0	0
D15	1026	C	14	0	0	0	14	0
D15	1041	T	14	13	0	0	0	0
D15	1047	G	14	0	0	0	14	0
D15	1050	A	14	0	9	0	5	0
D15	1053	A	14	0	0	14	0	0
D15	1062	G	14	14	0	0	0	0
D15	1078	G	14	0	0	0	14	0
D15	1080	T	14	0	14	0	0	0
D15	1083	G	14	1	0	0	0	0
D15	1121	T	14	1	0	0	0	0
D15	1134	T	14	0	1	0	0	0
D15	1138	A	14	0	0	1	0	0
D15	1144	T	14	0	1	0	0	0
D15	1167	A	14	0	0	0	13	0
D15	1171	C	14	0	0	4	0	0
D15	1173	C	14	0	0	0	4	0
D15	1179	A	14	0	1	0	0	0
D15	1219	C	14	13	0	0	0	0
D15	1221	A	14	0	1	0	0	0
D15	1228	A	14	0	13	0	0	0
D15	1229	A	14	0	0	12	0	0
D15	1232	G	14	1	0	0	0	0
D15	1234	C	14	13	0	0	0	0
D15	1321	A	14	0	13	0	0	0
D15	1329	A	14	0	0	0	13	0
D15	1332	T	14	0	13	0	0	0
D15	1341	C	14	0	0	0	13	0
D15	1347	T	14	0	13	0	0	0
D15	1356	T	14	0	0	13	0	0
D15	1365	C	14	1	0	0	0	0
D15	1370	T	14	5	0	0	0	0
D15	1376	G	14	1	0	0	0	0
D15	1383	A	14	0	1	6	0	0
D15	1386	C	14	0	0	13	0	0
D15	1434	G	14	0	0	0	4	0
D15	1438	G	14	1	0	0	0	0
D15	1458	T	14	0	13	0	0	0
D15	1467	G	14	4	0	0	0	0
D15	1479	G	14	0	0	0	13	0
D15	1480	G	14	0	13	0	0	0
D15	1485	C	14	0	0	0	1	0
D15	1498	T	14	0	13	0	0	0
D15	1499	G	14	8	0	0	0	0
D15	1500	T	14	0	7	0	0	0
D15	1512	G	14	8	0	0	0	0

KXY	1915	G	18	18	0	0	0	0
KXY	1922	C	18	0	0	18	0	0
KXY	1923	C	18	16	0	2	0	0
KXY	1933	A	18	0	0	0	18	0
KXY	1969	C	18	0	0	0	3	0
KXY	1975	T	18	0	0	18	0	0
KXY	1979	G	18	18	0	0	0	0
KXY	1984	T	18	0	0	18	0	0
KXY	2016	G	18	1	0	0	0	0
KXY	2019	A	18	0	0	0	1	0
KXY	2026	A	18	0	0	1	0	0
KXY	2065	C	18	18	0	0	0	0
KXY	2078	G	18	0	18	0	0	0
KXY	2086	G	18	0	1	0	0	0
KXY	2107	A	18	0	18	0	0	0
KXY	2134	T	18	0	18	0	0	0
KXY	2176	T	18	0	18	0	0	0
KXY	2178	T	18	0	1	0	0	0
KXY	2202	T	18	0	0	1	0	0
KXY	2209	C	18	0	0	0	16	0
KXY	2220	G	18	1	0	0	0	0
KXY	2232	G	18	1	0	0	0	0
KXY	2234	A	18	0	0	3	0	0
KXY	2248	T	18	18	0	0	0	0
KXY	2288	C	18	14	0	0	0	0
KXY	2350	A	18	0	0	1	0	0
KXY	2385	C	18	0	0	0	3	0
KXY	2408	T	18	0	3	0	0	0
KXY	2410	A	18	0	0	18	0	0
KXY	2430	A	18	0	0	5	0	0
KXY	2431	A	18	0	0	18	0	0
KXY	2443	T	18	0	1	0	0	0
KXY	2469	A	18	0	0	1	0	0
KXY	2525	G	18	0	18	0	0	0
KXY	2537	T	18	0	18	0	0	0
KXY	2540	C	18	0	0	0	18	0
KXY	2559	C	18	16	0	2	0	0
KXY	2560	G	18	18	0	0	0	0
KXY	2561	G	18	18	0	0	0	0
KXY	2564	A	18	0	0	18	0	0
KXY	2567	C	18	0	0	0	17	0
KXY	2576	T	18	0	18	0	0	0
KXY	2582	C	18	0	0	0	18	0
KXY	2591	C	18	0	0	0	18	0
KXY	2602	T	18	0	1	0	0	0
KXY	2603	C	18	0	0	0	18	0
KXY	2612	C	18	0	0	0	18	0
KXY	2633	A	18	0	0	18	0	0
KXY	2678	C	18	0	0	0	18	0
KXY	2715	G	18	18	0	0	0	0
KXY	2732	T	18	0	18	0	0	0
KXY	2733	A	18	0	18	0	0	0
KXY	2736	A	18	0	0	2	0	0
KXY	2738	T	18	0	18	0	0	0
KXY	2771	C	18	0	0	0	18	0
KXY	2775	C	18	0	0	0	18	0
KXY	2801	C	18	0	0	0	18	0
KXY	2848	A	18	0	0	1	0	0
KXY	2875	A	18	0	18	0	0	0
KXY	2892	T	18	0	18	0	0	0
KXY	2898	G	18	0	0	0	18	0
KXY	2927	A	18	0	0	18	0	0
KXY	2943	T	18	18	0	0	0	0
KXY	2950	G	18	3	0	0	0	0
KXY	3000	C	18	18	0	0	0	0
KXY	3009	G	18	17	0	0	0	0
KXY	3015	T	18	0	18	0	0	0
KXY	3020	T	18	0	1	0	0	0
KXY	3026	C	18	0	0	0	18	0
KXY	3030	A	18	0	0	3	0	0
KXY	3031	G	18	0	0	0	18	0
KXY	3057	C	18	18	0	0	0	0
KXY	3075	G	18	1	0	0	0	0
KXY	3079	A	18	0	0	1	0	0
KXY	3084	G	18	0	0	0	18	0
KXY	3105	T	18	17	1	0	0	0
KXY	3120	G	18	18	0	0	0	0
KXY	3195	T	18	0	1	0	0	0
KXY	3204	T	18	0	18	0	0	0
KXY	3210	G	18	1	0	0	0	0
KXY	3215	A	18	0	0	1	0	0
WR	30	A	15	0	0	2	0	0
WR	31	T	15	0	15	0	0	0
WR	87	A	15	0	0	1	0	0
WR	109	T	15	15	0	0	0	0
WR	136	T	15	1	0	0	0	0
WR	149	G	15	0	15	0	0	0
WR	235	A	15	0	0	1	0	0
WR	312	T	15	0	15	0	0	0
WR	357	C	15	0	0	0	15	0
WR	396	T	15	0	1	0	0	0
WR	400	A	15	0	0	1	0	0
WR	450	A	15	0	0	3	0	0
WR	493	T	15	15	0	0	0	0
WR	502	C	15	1	0	0	0	0
WR	530	A	15	0	0	2	0	0
WR	531	T	15	0	0	15	0	0
WR	532	A	15	0	0	0	15	0
WR	592	T	15	0	15	0	0	0
WR	593	T	15	0	1	0	0	0
WR	599	T	15	0	3	0	0	0
WR	639	T	15	15	0	0	0	0
WR	660	G	15	1	0	0	0	0
WR	690	T	15	0	1	0	0	0
WR	735	C	15	0	0	0	15	0
WR	798	T	15	0	1	0	0	0
WR	820	C	15	0	0	0	15	0
WR	834	A	15	0	0	15	0	0
WR	840	C	15	0	0	0	15	0
WR	843	C	15	0	0	0	15	0
WR	867	T	15	0	15	0	0	0
WR	873	C	15	0	0	0	15	0
WR	876	T	15	0	15	0	0	0
WR	882	C	15	15	0	0	0	0
WR	906	A	15	0	0	0	15	0
WR	909	G	15	15	0	0	0	0
WR	915	G	15	15	0	0	0	0
WR	923	T	15	0	1	0	0	0
WR	930	A	15	0	0	0	15	0
WR	941	C	15	15	0	0	0	0
WR	951	G	15	15	0	0	0	0

D15	1515	A	14	0	0	13	0	0
D15	1551	G	14	1	0	0	0	0
D15	1598	T	14	0	1	0	0	0
D15	1610	G	14	4	0	0	0	0
D15	1630	G	14	14	0	0	0	0
D15	1637	A	14	0	14	0	0	0
D15	1652	A	14	0	0	1	0	0
D15	1653	T	14	0	14	0	0	0
D15	1679	G	14	14	0	0	0	0
D15	1721	A	14	0	0	14	0	0
D15	1748	G	14	0	0	0	1	0
D15	1753	T	14	0	14	0	0	0
D15	1762	A	14	0	0	0	14	0
D15	1766	T	14	0	14	0	0	0
D15	1768	A	14	0	0	0	14	0
D15	1774	T	14	0	1	0	0	0
D15	1846	T	14	14	0	0	0	0
D15	1896	A	14	0	0	14	0	0
D15	1899	G	14	9	0	0	0	0
D15	1913	A	14	0	14	0	0	0
D15	1922	C	14	0	0	14	0	0
D15	1923	C	14	14	0	0	0	0
D15	1933	A	14	0	0	0	14	0
D15	1936	T	14	0	1	0	0	0
D15	1937	G	14	0	0	0	14	0
D15	1979	G	14	14	0	0	0	0
D15	1984	T	14	0	14	0	0	0
D15	2003	T	14	0	0	14	0	0
D15	2017	G	14	6	0	0	0	0
D15	2050	T	14	0	1	0	0	0
D15	2073	C	14	14	0	0	0	0
D15	2078	G	14	0	14	0	0	0
D15	2107	A	14	0	14	0	0	0
D15	2134	T	14	0	5	0	0	0
D15	2149	A	14	0	0	0	4	0
D15	2150	T	14	0	9	0	0	0
D15	2158	A	14	0	14	0	0	0
D15	2161	C	14	4	0	0	0	0
D15	2209	C	14	0	0	0	14	0
D15	2342	G	14	14	0	0	0	0
D15	2355	G	14	4	0	0	0	0
D15	2365	C	14	1	0	0	0	0
D15	2410	A	14	0	0	14	0	0
D15	2443	T	14	0	4	0	0	0
D15	2444	C	14	0	0	0	4	0
D15	2537	T	14	0	14	0	0	0
D15	2558	G	14	14	0	0	0	0
D15	2560	G	14	14	0	0	0	0
D15	2564	A	14	0	0	14	0	0
D15	2573	C	14	0	0	0	14	0
D15	2574	A	14	0	0	9	0	0
D15	2582	C	14	0	0	0	14	0
D15	2585	A	14	0	0	1	0	0
D15	2591	C	14	0	0	0	14	0
D15	2594	G	14	14	0	0	0	0
D15	2603	C	14	0	0	0	14	0
D15	2606	A	14	0	0	0	9	0
D15	2612	C	14	0	0	0	14	0
D15	2657	T	14	0	0	1	0	0
D15	2662	T	14	1	0	8	0	0
D15	2681	A	14	0	0	14	0	0
D15	2684	T	14	0	14	0	0	0
D15	2687	A	14	0	0	14	0	0
D15	2699	T	14	0	0	14	0	0
D15	2713	A	14	0	0	1	0	0
D15	2717	A	14	0	6	0	0	0
D15	2732	T	14	0	14	0	0	0
D15	2733	A	14	0	14	0	0	0
D15	2741	A	14	0	0	14	0	0
D15	2743	A	14	0	6	0	0	0
D15	2774	T	14	0	1	0	0	0
D15	2780	T	14	0	5	0	0	0
D15	2798	C	14	14	0	0	0	0
D15	2875	A	14	0	10	0	0	0
D15	2898	G	14	0	1	0	13	0
D15	2958	T	14	0	14	0	0	0
D15	3008	C	14	14	0	0	0	0
D15	3012	A	14	0	14	0	0	0
D15	3015	T	14	0	0	5	0	0
D15	3026	C	14	0	0	0	10	0
D15	3030	A	14	0	0	1	0	0
D15	3057	C	14	0	0	10	0	0
D15	3064	A	14	0	0	10	0	0
D15	3077	G	14	0	10	0	0	0
D15	3084	G	14	0	0	0	10	0
D15	3100	T	14	0	1	0	0	0
D15	3105	T	14	10	0	0	0	0
D15	3123	T	14	0	1	0	0	0
D15	3148	T	14	0	0	1	0	0
D15	3192	T	14	0	9	0	0	0
D15	3195	T	14	0	1	0	0	0
D15	3204	T	14	0	14	0	0	0
D15	3205	A	14	0	0	9	0	0
D15	3206	T	14	0	5	0	0	0
D15	3207	G	14	9	0	0	0	0
D24	31	T	13	0	13	0	0	0
D24	58	T	13	3	0	0	0	0
D24	87	A	13	0	0	3	0	0
D24	109	T	13	13	0	0	0	0
D24	123	T	13	0	1	0	0	0
D24	145	T	13	0	1	0	0	0
D24	149	G	13	0	13	0	0	0
D24	162	A	13	0	0	13	0	0
D24	176	T	13	0	2	0	0	0
D24	184	A	13	0	0	1	0	0
D24	221	A	13	0	0	1	0	0
D24	245	A	13	0	0	1	0	0
D24	285	G	13	1	0	0	0	0
D24	290	C	13	0	0	0	2	0
D24	312	T	13	0	13	0	0	0
D24	357	C	13	0	0	0	13	0
D24	392	T	13	0	3	0	0	0
D24	493	T	13	13	0	0	0	0
D24	528	C	13	0	0	0	3	0
D24	532	A	13	0	0	0	13	0
D24	592	T	13	0	13	0	0	0
D24	637	C	13	0	0	0	3	0
D24	735	C	13	0	0	0	13	0
D24	820	C	13	0	0	0	13	0
D24	834	A	13	0	0	13	0	0
D24	840	C	13	0	0	0	13	0

WR	965	A	15	0	0	1	0	0
WR	969	C	15	15	0	0	0	0
WR	993	A	15	0	0	15	0	0
WR	1003	T	15	0	0	15	0	0
WR	1014	T	15	0	15	0	0	0
WR	1020	C	15	0	0	0	15	0
WR	1023	T	15	0	15	0	0	0
WR	1026	C	15	0	0	0	15	0
WR	1041	T	15	0	15	0	0	0
WR	1047	G	15	0	0	0	15	0
WR	1050	A	15	0	15	0	0	0
WR	1062	G	15	15	0	0	0	0
WR	1078	G	15	0	0	0	15	0
WR	1080	T	15	15	0	0	0	0
WR	1086	G	15	15	0	0	0	0
WR	1116	C	15	0	0	0	15	0
WR	1117	T	15	1	0	0	0	0
WR	1206	C	15	15	0	0	0	0
WR	1218	A	15	0	0	0	15	0
WR	1219	C	15	15	0	0	0	0
WR	1221	A	15	0	0	0	15	0
WR	1228	A	15	0	15	0	0	0
WR	1229	A	15	0	0	15	0	0
WR	1230	G	15	0	15	0	0	0
WR	1234	C	15	15	0	0	0	0
WR	1273	G	15	1	0	0	0	0
WR	1287	A	15	0	0	3	0	0
WR	1317	-	15	0	0	15	0	0
WR	1321	A	15	0	0	0	0	15
WR	1332	T	15	0	15	0	0	0
WR	1341	C	15	0	0	0	15	0
WR	1347	T	15	0	15	0	0	0
WR	1356	T	15	0	0	15	0	0
WR	1386	C	15	0	0	15	0	0
WR	1458	T	15	0	15	0	0	0
WR	1479	G	15	15	0	0	0	0
WR	1480	G	15	0	15	0	0	0
WR	1488	T	15	0	1	0	0	0
WR	1498	T	15	0	15	0	0	0
WR	1499	G	15	15	0	0	0	0
WR	1500	T	15	1	0	14	0	0
WR	1512	G	15	15	0	0	0	0
WR	1515	A	15	0	0	15	0	0
WR	1565	A	15	0	0	15	0	0
WR	1584	C	15	0	0	0	1	0
WR	1613	A	15	0	0	15	0	0
WR	1630	G	15	0	0	0	15	0
WR	1637	A	15	0	15	0	0	0
WR	1653	T	15	0	15	0	0	0
WR	1674	T	15	0	0	15	0	0
WR	1679	G	15	15	0	0	0	0
WR	1693	A	15	0	0	1	0	0
WR	1719	G	15	0	0	0	15	0
WR	1721	A	15	0	0	15	0	0
WR	1727	A	15	0	0	15	0	0
WR	1735	A	15	0	0	0	1	0
WR	1741	T	15	0	15	0	0	0
WR	1749	G	15	0	1	0	0	0
WR	1762	A	15	0	0	0	15	0
WR	1768	A	15	0	0	0	15	0
WR	1784	T	15	0	1	0	0	0
WR	1798	T	15	0	0	1	0	0
WR	1846	T	15	15	0	0	0	0
WR	1896	A	15	0	0	15	0	0
WR	1913	A	15	0	15	0	0	0
WR	1922	C	15	0	0	15	0	0
WR	1923	C	15	15	0	0	0	0
WR	1933	A	15	0	0	0	15	0
WR	1938	T	15	0	1	0	0	0
WR	1951	T	15	0	1	0	0	0
WR	1961	T	15	0	0	15	0	0
WR	1971	T	15	0	2	0	0	0
WR	1979	G	15	15	0	0	0	0
WR	1984	T	15	15	0	0	0	0
WR	2005	T	15	15	0	0	0	0
WR	2019	A	15	0	0	1	0	0
WR	2078	G	15	0	15	0	0	0
WR	2080	G	15	15	0	0	0	0
WR	2107	A	15	0	15	0	0	0
WR	2134	T	15	0	15	0	0	0
WR	2141	T	15	0	1	0	0	0
WR	2144	A	15	0	0	1	0	0
WR	2158	A	15	0	15	0	0	0
WR	2175	A	15	0	0	1	0	0
WR	2201	T	15	0	15	0	0	0
WR	2209	C	15	0	0	0	15	0
WR	2242	T	15	0	0	15	0	0
WR	2256	T	15	0	3	0	0	0
WR	2288	C	15	15	0	0	0	0
WR	2290	C	15	0	0	0	15	0
WR	2399	A	15	0	0	1	0	0
WR	2406	A	15	0	0	1	0	0
WR	2410	A	15	0	0	15	0	0
WR	2439	A	15	0	0	15	0	0
WR	2480	C	15	0	0	0	15	0
WR	2537	T	15	0	15	0	0	0
WR	2540	C	15	0	0	0	15	0
WR	2547	C	15	15	0	0	0	0
WR	2558	G	15	15	0	0	0	0
WR	2560	G	15	15	0	0	0	0
WR	2564	A	15	0	0	15	0	0
WR	2573	C	15	0	0	0	15	0
WR	2582	C	15	0	0	0	14	0
WR	2591	C	15	0	0	0	14	0
WR	2603	C	15	0	0	0	15	0
WR	2612	C	15	0	0	0	15	0
WR	2621	G	15	3	0	0	0	0
WR	2651	C	15	0	0	0	15	0
WR	2673	T	15	0	15	0	0	0
WR	2681	A	15	0	0	15	0	0
WR	2684	T	15	0	15	0	0	0
WR	2699	T	15	15	0	0	0	0
WR	2703	T	15	0	0	1	0	0
WR	2720	T	15	0	1	0	0	0
WR	2721	A	15	0	0	0	1	0
WR	2730	T	15	0	1	0	0	0
WR	2732	T	15	0	15	0	0	0
WR	2741	A	15	0	0	15	0	0
WR	2768	T	15	0	0	15	0	0
WR	2782	A	15	0	1	0	0	0
WR	2789	A	15	0	0	1	0	0
WR	2810	T	15	0	1	0	0	0

D24	843	C	13	0	0	0	13	0
D24	867	T	13	0	13	0	0	0
D24	876	T	13	0	13	0	0	0
D24	882	C	13	13	0	0	0	0
D24	906	A	13	0	0	0	13	0
D24	909	G	13	13	0	0	0	0
D24	912	A	13	0	0	13	0	0
D24	915	G	13	13	0	0	0	0
D24	934	A	13	0	13	0	0	0
D24	936	C	13	0	0	0	13	0
D24	941	C	13	13	0	0	0	0
D24	951	G	13	13	0	0	0	0
D24	967	A	13	0	13	0	0	0
D24	969	C	13	13	0	0	0	0
D24	983	A	13	0	0	1	0	0
D24	993	A	13	0	0	13	0	0
D24	1003	T	13	0	0	13	0	0
D24	1014	T	13	0	13	0	0	0
D24	1020	C	13	0	0	0	13	0
D24	1023	T	13	0	13	0	0	0
D24	1026	C	13	0	0	0	13	0
D24	1041	T	13	0	13	0	0	0
D24	1047	G	13	0	0	0	13	0
D24	1050	A	13	0	0	0	13	0
D24	1062	G	13	13	0	0	0	0
D24	1078	G	13	0	13	0	0	0
D24	1079	C	13	13	0	0	0	0
D24	1117	T	13	0	1	0	0	0
D24	1122	G	13	0	0	0	13	0
D24	1194	A	13	0	0	1	0	0
D24	1206	C	13	13	0	0	0	0
D24	1218	A	13	0	0	0	13	0
D24	1219	C	13	13	0	0	0	0
D24	1221	A	13	0	0	0	13	0
D24	1228	A	13	0	13	0	0	0
D24	1229	A	13	0	0	13	0	0
D24	1230	G	13	0	13	0	0	0
D24	1234	C	13	13	0	0	0	0
D24	1248	T	13	0	3	0	0	0
D24	1272	T	13	0	1	0	0	0
D24	1320	C	13	13	0	0	0	0
D24	1321	A	13	0	13	0	0	0
D24	1323	C	13	0	0	0	13	0
D24	1329	A	13	0	13	0	0	0
D24	1332	T	13	0	13	0	0	0
D24	1341	C	13	0	0	0	13	0
D24	1347	T	13	0	13	0	0	0
D24	1356	T	13	0	0	13	0	0
D24	1359	A	13	0	0	13	0	0
D24	1386	C	13	0	0	13	0	0
D24	1425	C	13	0	0	0	13	0
D24	1458	T	13	0	13	0	0	0
D24	1479	G	13	13	0	0	0	0
D24	1480	G	13	0	13	0	0	0
D24	1498	T	13	0	13	0	0	0
D24	1499	G	13	13	0	0	0	0
D24	1515	A	13	0	0	13	0	0
D24	1538	A	13	0	0	1	0	0
D24	1613	A	13	0	0	13	0	0
D24	1630	G	13	13	0	0	0	0
D24	1637	A	13	0	11	0	2	0
D24	1653	T	13	0	13	0	0	0
D24	1679	G	13	13	0	0	0	0
D24	1708	T	13	0	3	0	0	0
D24	1719	G	13	0	0	0	13	0
D24	1721	A	13	0	0	13	0	0
D24	1727	A	13	0	0	13	0	0
D24	1764	A	13	0	0	13	0	0
D24	1766	T	13	0	13	0	0	0
D24	1768	A	13	0	0	0	13	0
D24	1769	T	13	0	1	0	0	0
D24	1846	T	13	13	0	0	0	0
D24	1861	T	13	0	3	0	0	0
D24	1880	T	13	0	1	0	0	0
D24	1896	A	13	0	0	13	0	0
D24	1907	A	13	0	0	1	0	0
D24	1913	A	13	0	13	0	0	0
D24	1922	C	13	0	0	13	0	0
D24	1923	C	13	13	0	0	0	0
D24	1931	G	13	1	0	0	0	0
D24	1933	A	13	0	0	0	13	0
D24	1969	C	13	0	0	0	13	0
D24	1979	G	13	13	0	0	0	0
D24	1984	T	13	13	0	0	0	0
D24	1993	C	13	0	0	0	13	0
D24	2017	G	13	13	0	0	0	0
D24	2078	G	13	0	13	0	0	0
D24	2080	G	13	0	0	0	13	0
D24	2107	A	13	0	13	0	0	0
D24	2134	T	13	0	13	0	0	0
D24	2158	A	13	0	13	0	0	0
D24	2201	T	13	0	13	0	0	0
D24	2209	C	13	0	0	0	13	0
D24	2238	A	13	0	0	1	0	0
D24	2245	T	13	0	13	0	0	0
D24	2260	G	13	13	0	0	0	0
D24	2290	C	13	0	0	0	13	0
D24	2297	A	13	0	0	1	0	0
D24	2410	A	13	0	0	13	0	0
D24	2537	T	13	0	13	0	0	0
D24	2558	G	13	13	0	0	0	0
D24	2560	G	13	13	0	0	0	0
D24	2564	A	13	0	0	13	0	0
D24	2566	A	13	0	0	1	0	0
D24	2573	C	13	0	0	0	13	0
D24	2582	C	13	0	0	0	13	0
D24	2591	C	13	0	0	0	13	0
D24	2603	C	13	0	0	0	13	0
D24	2612	C	13	0	0	0	13	0
D24	2614	A	13	0	0	3	0	0
D24	2681	A	13	0	0	13	0	0
D24	2684	T	13	0	13	0	0	0
D24	2699	T	13	13	0	0	0	0
D24	2715	G	13	1	0	0	0	0
D24	2720	T	13	0	1	0	0	0
D24	2732	T	13	0	13	0	0	0
D24	2741	A	13	0	0	13	0	0
D24	2797	G	13	13	0	0	0	0
D24	2875	A	13	0	13	0	0	0
D24	2889	G	13	13	0	0	0	0
D24	2958	T	13	0	13	0	0	0
D24	2997	T	13	0	1	0	0	0

WR	2875	A	15	0	15	0	0	0
WR	2898	G	15	0	0	0	15	0
WR	2909	C	15	1	0	0	0	0
WR	2946	G	15	15	0	0	0	0
WR	2954	C	15	1	0	0	0	0
WR	2958	T	15	0	15	0	0	0
WR	3008	C	15	15	0	0	0	0
WR	3041	C	15	0	0	0	1	0
WR	3064	A	15	0	0	15	0	0
WR	3084	G	15	0	0	0	15	0
WR	3105	T	15	15	0	0	0	0
WR	3116	C	15	0	0	0	15	0
WR	3120	G	15	15	0	0	0	0
WR	3202	G	15	1	0	0	0	0
WR	3204	T	15	0	15	0	0	0

XDH	10	A	12	0	12	0	0	0
XDH	13	C	12	0	0	0	12	0
XDH	64	T	12	0	5	0	0	0
XDH	149	G	12	0	12	0	0	0
XDH	197	T	12	0	1	0	0	0
XDH	241	A	12	0	0	12	0	0
XDH	254	T	12	0	1	0	0	0
XDH	260	T	12	0	1	0	0	0
XDH	358	T	12	0	1	0	0	0
XDH	414	T	12	0	1	0	0	0
XDH	442	T	12	0	1	0	0	0
XDH	505	C	12	0	0	0	12	0
XDH	523	C	12	1	0	0	0	0
XDH	678	T	12	0	12	0	0	0
XDH	820	C	12	0	0	0	12	0
XDH	840	C	12	0	0	0	12	0
XDH	882	C	12	0	0	0	12	0
XDH	886	G	12	3	0	0	0	0
XDH	907	T	12	0	12	0	0	0
XDH	925	G	12	1	0	0	0	0
XDH	941	C	12	12	0	0	0	0
XDH	969	C	12	12	0	0	0	0
XDH	995	G	12	1	0	0	0	0
XDH	1003	T	12	0	0	12	0	0
XDH	1009	T	12	0	12	0	0	0
XDH	1014	T	12	0	12	0	0	0
XDH	1020	C	12	0	0	0	12	0
XDH	1023	T	12	0	12	0	0	0
XDH	1026	C	12	0	0	0	12	0
XDH	1047	G	12	0	12	0	0	0
XDH	1083	G	12	9	0	0	0	0
XDH	1206	C	12	12	0	0	0	0
XDH	1234	C	12	12	0	0	0	0
XDH	1296	T	12	0	1	0	0	0
XDH	1317	A	12	0	0	12	0	0
XDH	1323	C	12	0	0	0	12	0
XDH	1341	C	12	0	0	0	12	0
XDH	1347	T	12	0	12	0	0	0
XDH	1353	T	12	0	0	12	0	0
XDH	1356	T	12	0	0	12	0	0
XDH	1410	C	12	12	0	0	0	0
XDH	1458	T	12	0	12	0	0	0
XDH	1515	A	12	0	0	12	0	0
XDH	1555	G	12	5	0	0	0	0
XDH	1563	T	12	12	0	0	0	0
XDH	1580	T	12	0	1	0	0	0
XDH	1613	A	12	0	0	11	0	0
XDH	1629	C	12	3	0	0	0	0
XDH	1653	T	12	0	12	0	0	0
XDH	1721	A	12	0	0	12	0	0
XDH	1727	A	12	0	0	1	0	0
XDH	1762	A	12	0	0	0	1	0
XDH	1764	A	12	0	0	11	0	0
XDH	1766	T	12	0	12	0	0	0
XDH	1768	A	12	0	0	0	12	0
XDH	1846	T	12	12	0	0	0	0
XDH	1850	T	12	0	3	0	0	0
XDH	1896	A	12	0	0	12	0	0
XDH	1913	A	12	0	12	0	0	0
XDH	1922	C	12	0	0	12	0	0
XDH	1923	C	12	12	0	0	0	0
XDH	1933	A	12	0	0	0	12	0
XDH	1979	G	12	12	0	0	0	0
XDH	2008	T	12	0	12	0	0	0
XDH	2078	G	12	0	12	0	0	0
XDH	2134	T	12	0	12	0	0	0
XDH	2244	T	12	0	3	0	0	0
XDH	2377	A	12	0	0	5	0	0
XDH	2459	T	12	0	1	0	0	0
XDH	2537	T	12	0	12	0	0	0
XDH	2560	G	12	11	0	0	0	0
XDH	2574	A	12	0	0	12	0	0
XDH	2591	C	12	0	0	0	12	0
XDH	2594	G	12	12	0	0	0	0
XDH	2604	A	12	0	0	0	1	0
XDH	2607	G	12	12	0	0	0	0
XDH	2720	T	12	0	1	0	0	0
XDH	2732	T	12	0	12	0	0	0
XDH	2733	A	12	0	12	0	0	0
XDH	2801	C	12	0	0	0	12	0
XDH	2887	A	12	0	0	0	5	0
XDH	2898	G	12	0	0	0	12	0
XDH	2968	C	12	0	0	0	12	0
XDH	3009	G	12	12	0	0	0	0
XDH	3105	T	12	12	0	0	0	0
XDH	3171	C	12	0	0	0	12	0
XDH	3198	T	12	0	1	0	0	0
XDH	3204	T	12	0	12	0	0	0

LMX	25	G	16	15	0	0	0	0
LMX	36	G	16	1	0	0	0	0
LMX	107	T	16	0	1	0	0	0
LMX	134	A	16	0	0	3	0	0
LMX	159	A	16	0	0	1	0	0
LMX	168	C	16	0	0	1	0	0
LMX	172	A	16	0	0	0	1	0
LMX	286	A	16	0	0	1	0	0
LMX	302	G	16	1	0	0	0	0
LMX	364	T	16	0	1	0	0	0
LMX	433	C	16	0	0	0	1	0
LMX	509	G	16	1	0	0	0	0
LMX	512	C	16	1	0	0	0	0
LMX	574	C	16	4	0	0	0	0
LMX	614	T	16	2	0	0	0	0
LMX	756	G	16	1	0	0	0	0
LMX	763	A	16	0	0	2	0	0

D24	3008	C	13	13	0	0	0	0
D24	3049	A	13	0	0	1	0	0
D24	3051	T	13	0	13	0	0	0
D24	3057	C	13	13	0	0	0	0
D24	3064	A	13	0	0	13	0	0
D24	3084	G	13	0	0	0	13	0
D24	3100	T	13	0	13	0	0	0
D24	3105	T	13	13	0	0	0	0
D24	3110	T	13	0	3	0	0	0
D24	3116	C	13	0	0	0	13	0
D24	3120	G	13	13	0	0	0	0
D24	3160	C	13	0	0	0	4	0
D24	3204	T	13	0	13	0	0	0
D23	35	A	20	0	1	0	0	0
D23	49	A	20	0	0	1	0	0
D23	52	C	20	0	0	0	20	0
D23	64	T	20	0	0	1	0	0
D23	68	T	20	0	1	0	0	0
D23	93	T	20	0	0	1	0	0
D23	109	T	20	20	0	0	0	0
D23	113	A	20	0	1	0	0	0
D23	122	A	20	0	0	2	0	0
D23	125	T	20	1	1	0	0	0
D23	149	G	20	0	20	0	0	0
D23	162	A	20	0	0	20	0	0
D23	184	A	20	0	0	1	0	0
D23	195	T	20	0	1	0	0	0
D23	277	T	20	0	2	0	0	0
D23	286	A	20	0	0	1	0	0
D23	312	T	20	0	20	0	0	0
D23	362	C	20	0	0	0	1	0
D23	364	T	20	0	1	0	0	0
D23	381	G	20	7	0	0	0	0
D23	434	T	20	0	1	0	0	0
D23	441	T	20	0	1	0	0	0
D23	446	C	20	0	0	10	0	0
D23	450	A	20	0	0	1	0	0
D23	465	T	20	0	1	0	0	0
D23	473	T	20	0	1	0	0	0
D23	493	T	20	20	0	0	0	0
D23	514	A	20	0	0	5	0	0
D23	531	T	20	0	10	0	0	0
D23	532	A	20	0	0	0	20	0
D23	543	G	20	1	0	0	0	0
D23	592	T	20	0	20	0	0	0
D23	616	A	20	0	0	20	0	0
D23	634	A	20	0	0	1	0	0
D23	657	T	20	0	1	0	0	0
D23	674	A	20	0	0	0	1	0
D23	707	G	20	2	0	0	0	0
D23	735	C	20	0	0	0	20	0
D23	739	A	20	0	0	1	0	0
D23	744	T	20	0	1	0	0	0
D23	820	C	20	0	0	0	20	0
D23	832	T	20	0	2	0	0	0
D23	834	A	20	0	0	20	0	0
D23	837	C	20	0	0	0	20	0
D23	840	C	20	0	0	0	20	0
D23	843	C	20	0	0	0	20	0
D23	867	T	20	0	20	0	0	0
D23	876	T	20	0	20	0	0	0
D23	882	C	20	20	0	0	0	0
D23	906	A	20	0	0	0	20	0
D23	909	G	20	20	0	0	0	0
D23	912	A	20	0	0	20	0	0
D23	929	T	20	19	0	0	0	0
D23	934	A	20	0	20	0	0	0
D23	941	C	20	20	0	0	0	0
D23	955	C	20	0	0	0	20	0
D23	963	A	20	0	6	0	0	0
D23	969	C	20	20	0	0	0	0
D23	1001	T	20	0	0	0	0	0
D23	1002	G	20	2	0	0	0	0
D23	1003	T	20	0	0	20	0	0
D23	1011	G	20	14	0	0	0	0
D23	1014	T	20	0	20	0	0	0
D23	1020	C	20	0	0	0	20	0
D23	1023	T	20	0	20	0	0	0
D23	1026	C	20	0	0	0	20	0
D23	1041	T	20	0	20	0	0	0
D23	1047	G	20	0	0	0	20	0
D23	1050	A	20	0	20	0	0	0
D23	1062	G	20	20	0	0	0	0
D23	1114	G	20	1	0	0	0	0
D23	1116	C	20	0	0	0	20	0
D23	1139	A	20	0	0	1	0	0
D23	1183	T	20	0	1	0	0	0
D23	1206	C	20	20	0	0	0	0
D23	1218	A	20	0	20	0	0	0
D23	1219	C	20	20	0	0	0	0
D23	1228	A	20	0	20	0	0	0
D23	1234	C	20	20	0	0	0	0
D23	1306	C	20	20	0	0	0	0
D23	1311	T	20	0	1	0	0	0
D23	1317	-	20	0	0	20	0	0
D23	1321	A	20	0	0	0	0	20
D23	1323	C	20	0	0	0	20	0
D23	1329	A	20	0	19	0	1	0
D23	1332	T	20	0	20	0	0	0
D23	1341	C	20	0	0	0	20	0
D23	1346	T	20	0	1	0	0	0
D23	1347	T	20	0	20	0	0	0
D23	1356	T	20	0	0	20	0	0
D23	1386	C	20	0	0	20	0	0
D23	1458	T	20	0	20	0	0	0
D23	1476	G	20	8	0	0	0	0
D23	1479	G	20	0	0	0	20	0
D23	1480	G	20	0	20	0	0	0
D23	1486	C	20	0	0	0	1	0
D23	1498	T	20	0	20	0	0	0
D23	1499	G	20	19	0	0	0	0
D23	1515	A	20	0	0	20	0	0
D23	1563	T	20	0	1	0	0	0
D23	1612	A	20	0	1	0	0	0
D23	1613	A	20	0	0	20	0	0
D23	1630	G	20	20	0	0	0	0
D23	1637	A	20	0	20	0	0	0
D23	1653	T	20	0	20	0	0	0
D23	1659	A	20	0	0	1	0	0
D23	1660	G	20	1	0	0	0	0
D23	1674	T	20	0	10	0	0	0

LMX	836	A	16	0	0	0	1	0
LMX	837	C	16	0	0	0	16	0
LMX	838	T	16	0	1	0	0	0
LMX	852	G	16	1	0	0	0	0
LMX	860	G	16	0	0	0	1	0
LMX	874	T	16	0	1	0	0	0
LMX	912	A	16	0	0	2	0	0
LMX	918	T	16	0	0	2	0	0
LMX	925	A	16	0	0	1	0	0
LMX	930	G	16	0	1	0	0	0
LMX	931	A	16	0	0	1	0	0
LMX	942	G	16	16	0	0	0	0
LMX	951	A	16	0	0	1	0	0
LMX	988	T	16	0	2	0	0	0
LMX	1009	C	16	0	0	0	16	0
LMX	1054	A	16	0	0	1	0	0
LMX	1124	A	16	0	0	16	0	0
LMX	1125	C	16	0	0	0	16	0
LMX	1175	A	16	0	0	16	0	0
LMX	1219	A	16	0	0	0	16	0
LMX	1250	T	16	0	0	1	0	0
LMX	1252	T	16	0	0	16	0	0
LMX	1309	T	16	0	2	0	0	0
LMX	1320	C	16	16	0	0	0	0
LMX	1362	C	16	0	0	0	16	0
LMX	1376	G	16	1	0	0	0	0
LMX	1437	A	16	0	0	16	0	0
LMX	1474	T	16	0	2	0	0	0
LMX	1485	T	16	0	16	0	0	0
LMX	1497	C	16	0	0	0	16	0
LMX	1499	G	16	16	0	0	0	0
LMX	1508	T	16	16	0	0	0	0
LMX	1596	T	16	2	0	0	0	0
LMX	1613	G	16	2	0	0	0	0
LMX	1620	G	16	16	0	0	0	0
LMX	1632	A	16	0	16	0	0	0
LMX	1635	G	16	16	0	0	0	0
LMX	1649	C	16	16	0	0	0	0
LMX	1659	A	16	0	16	0	0	0
LMX	1676	A	16	0	0	0	1	0
LMX	1705	A	16	0	0	2	0	0
LMX	1762	A	16	0	0	0	6	0
LMX	1764	A	16	0	0	10	0	0
LMX	1769	C	16	0	0	0	16	0
LMX	1786	G	16	0	0	0	2	0
LMX	1820	A	16	0	16	0	0	0
LMX	1830	T	16	0	1	0	0	0
LMX	1875	A	16	0	0	1	0	0
LMX	2011	C	16	0	0	15	0	0
LMX	2023	C	16	15	0	0	0	0
LMX	2035	A	16	0	0	15	0	0
LMX	2063	C	16	2	0	0	0	0
LMX	2074	T	16	16	0	0	0	0
LMX	2098	G	16	0	1	0	0	0
LMX	2127	T	16	0	0	3	0	0
LMX	2147	G	16	1	0	0	0	0
LMX	2178	T	16	0	2	0	0	0
LMX	2188	A	16	0	0	1	0	0
LMX	2191	C	16	0	0	0	5	0
LMX	2203	T	16	5	0	0	0	0
LMX	2206	G	16	1	0	0	0	0
LMX	2242	T	16	1	0	0	0	0
LMX	2245	C	16	0	0	0	16	0
LMX	2278	T	16	0	1	0	0	0
LMX	2306	A	16	0	0	1	0	0
LMX	2307	A	16	0	0	2	0	0
LMX	2318	T	16	0	1	0	0	0
LMX	2327	C	16	1	0	0	0	0
LMX	2330	C	16	0	0	0	1	0
LMX	2339	A	16	0	0	3	0	0
LMX	2375	A	16	0	0	2	0	0
LMX	2438	G	16	1	0	0	0	0
LMX	2477	A	16	0	0	2	0	0
LMX	2563	A	16	0	0	2	0	0
LMX	2573	C	16	0	0	0	3	0
LMX	2574	A	16	0	16	0	0	0
LMX	2614	A	16	0	0	1	0	0
LMX	2630	C	16	4	0	0	0	0
LMX	2631	T	16	0	1	0	0	0
LMX	2675	A	16	0	0	16	0	0
LMX	2681	G	16	16	0	0	0	0
LMX	2810	C	16	0	0	0	16	0
LMX	2851	G	16	1	0	0	0	0
LMX	2889	G	16	2	0	0	0	0
LMX	2901	T	16	0	1	0	0	0
LMX	2946	A	16	0	0	16	0	0
LMX	2963	A	16	0	0	1	0	0
LMX	2973	G	16	0	0	0	16	0
LMX	2980	T	16	0	3	0	0	0
LMX	3026	C	16	0	0	0	15	0
LMX	3051	T	16	0	16	0	0	0
LMX	3069	T	16	0	0	7	0	0
LMX	3099	T	16	16	0	0	0	0
LMX	3100	T	16	0	2	0	0	0
LMX	3185	G	16	1	0	0	0	0

D23	1679	G	20	20	0	0	0	0
D23	1708	T	20	0	1	0	0	0
D23	1719	G	20	0	0	0	20	0
D23	1721	A	20	0	0	20	0	0
D23	1731	T	20	0	1	0	0	0
D23	1762	A	20	0	0	0	20	0
D23	1766	T	20	0	20	0	0	0
D23	1768	A	20	0	0	0	20	0
D23	1778	A	20	0	0	2	0	0
D23	1790	T	20	1	0	0	0	0
D23	1805	A	20	0	0	2	0	0
D23	1846	T	20	20	0	0	0	0
D23	1863	T	20	0	1	0	0	0
D23	1896	A	20	0	0	19	0	0
D23	1913	A	20	0	20	0	0	0
D23	1922	C	20	0	0	20	0	0
D23	1923	C	20	20	0	0	0	0
D23	1930	A	20	0	0	1	0	0
D23	1933	A	20	0	0	0	20	0
D23	1963	T	20	0	1	0	0	0
D23	1966	C	20	0	0	0	1	0
D23	1979	G	20	20	0	0	0	0
D23	1981	T	20	0	1	0	0	0
D23	1984	T	20	19	0	1	0	0
D23	1999	C	20	0	0	0	2	0
D23	2065	C	20	3	0	0	0	0
D23	2078	G	20	0	20	0	0	0
D23	2096	A	20	0	0	1	0	0
D23	2107	A	20	0	20	0	0	0
D23	2134	T	20	0	20	0	0	0
D23	2158	A	20	0	20	0	0	0
D23	2159	A	20	0	0	1	0	0
D23	2184	T	20	1	0	0	0	0
D23	2189	A	20	0	1	0	0	0
D23	2202	T	20	0	1	1	0	0
D23	2209	C	20	0	0	0	20	0
D23	2216	T	20	0	1	0	0	0
D23	2227	T	20	0	0	1	0	0
D23	2299	A	20	0	0	2	0	0
D23	2304	C	20	1	0	0	0	0
D23	2323	A	20	0	0	0	2	0
D23	2410	A	20	0	0	20	0	0
D23	2430	A	20	0	0	2	0	0
D23	2451	A	20	0	0	1	0	0
D23	2456	C	20	0	0	0	3	0
D23	2468	T	20	0	20	0	0	0
D23	2489	G	20	2	0	0	0	0
D23	2537	T	20	0	20	0	0	0
D23	2541	T	20	0	1	0	0	0
D23	2558	G	20	20	0	0	0	0
D23	2560	G	20	20	0	0	0	0
D23	2564	A	20	0	0	20	0	0
D23	2573	C	20	0	0	0	20	0
D23	2582	C	20	0	0	0	20	0
D23	2590	A	20	0	0	1	0	0
D23	2591	C	20	0	0	0	20	0
D23	2594	G	20	20	0	0	0	0
D23	2603	C	20	0	0	0	20	0
D23	2612	C	20	0	0	0	20	0
D23	2622	A	20	0	0	1	0	0
D23	2627	A	20	0	0	1	0	0
D23	2660	C	20	0	0	0	20	0
D23	2681	A	20	0	0	20	0	0
D23	2684	T	20	0	20	0	0	0
D23	2699	T	20	1	0	19	0	0
D23	2708	T	20	0	0	1	0	0
D23	2712	C	20	0	0	0	19	0
D23	2732	T	20	0	20	0	0	0
D23	2740	G	20	0	0	0	1	0
D23	2741	A	20	0	0	20	0	0
D23	2771	C	20	0	0	0	19	0
D23	2849	T	20	0	1	0	0	0
D23	2856	T	20	0	1	0	0	0
D23	2875	A	20	0	20	0	0	0
D23	2890	A	20	0	0	1	0	0
D23	2898	G	20	0	1	0	19	0
D23	2900	T	20	0	0	1	0	0
D23	2928	T	20	0	1	0	0	0
D23	2952	A	20	0	0	1	0	0
D23	2958	T	20	0	20	0	0	0
D23	2995	G	20	1	0	0	0	0
D23	3000	C	20	0	0	0	20	0
D23	3008	C	20	20	0	0	0	0
D23	3013	A	20	0	0	3	0	0
D23	3046	T	20	0	1	0	0	0
D23	3051	T	20	0	20	0	0	0
D23	3057	C	20	20	0	0	0	0
D23	3064	A	20	0	0	20	0	0
D23	3066	T	20	0	1	0	0	0
D23	3084	G	20	0	0	0	20	0
D23	3099	A	20	0	1	0	0	0
D23	3105	T	20	20	0	0	0	0
D23	3120	G	20	20	0	0	0	0
D23	3150	A	20	0	0	2	0	0
D23	3160	C	20	0	0	0	1	0
D23	3172	T	20	0	1	0	0	0
D23	3180	T	20	0	2	0	0	0
D23	3204	T	20	0	20	0	0	0
D02	6	C	16	0	0	0	1	0
D02	9	C	16	0	0	0	1	0
D02	10	C	16	0	0	5	11	0
D02	19	A	16	0	0	0	11	0
D02	24	T	16	1	0	0	0	0
D02	29	G	16	10	0	0	0	0
D02	36	G	16	16	0	0	0	0
D02	176	T	16	0	1	0	0	0
D02	189	T	16	0	0	11	0	0
D02	213	T	16	0	1	0	0	0
D02	241	G	16	10	0	0	0	0
D02	285	G	16	1	0	0	0	0
D02	323	A	16	0	0	1	0	0
D02	333	A	16	0	11	0	0	0
D02	339	C	16	0	0	0	11	0
D02	382	T	16	0	1	0	0	0
D02	387	G	16	11	0	0	0	0
D02	482	A	16	0	11	0	0	0
D02	495	C	16	0	0	0	1	0
D02	514	A	16	0	0	3	0	0
D02	522	C	16	0	0	0	2	0
D02	554	T	16	11	0	0	0	0
D02	558	C	16	0	0	0	8	0

D02	574	C	16	7	0	0	0	0
D02	587	G	16	8	0	0	0	0
D02	591	A	16	0	1	0	0	0
D02	636	A	16	0	0	0	7	0
D02	667	C	16	0	0	0	3	0
D02	670	G	16	1	0	0	0	0
D02	686	C	16	0	0	0	1	0
D02	688	A	16	0	16	0	0	0
D02	700	G	16	1	0	0	0	0
D02	705	T	16	0	0	1	0	0
D02	717	C	16	0	0	0	1	0
D02	739	A	16	0	0	1	0	0
D02	748	G	16	8	0	0	0	0
D02	749	T	16	1	0	0	0	0
D02	750	G	16	8	0	0	0	0
D02	770	T	16	0	8	0	0	0
D02	771	A	16	0	0	1	0	0
D02	798	T	16	0	1	0	0	0
D02	832	T	16	0	3	0	0	0
D02	837	C	16	0	0	0	8	0
D02	838	T	16	0	16	0	0	0
D02	849	T	16	5	0	0	0	0
D02	853	A	16	0	5	0	0	0
D02	912	A	16	0	0	8	0	0
D02	927	A	16	0	0	1	0	0
D02	942	G	16	15	0	0	0	0
D02	970	C	16	0	0	0	1	0
D02	981	G	16	0	1	0	0	0
D02	987	A	16	0	0	0	1	0
D02	994	A	16	0	6	0	0	0
D02	1000	T	16	0	0	2	0	0
D02	1009	C	16	0	0	0	16	0
D02	1029	T	16	0	15	0	0	0
D02	1042	T	16	0	1	0	0	0
D02	1044	C	16	0	0	0	16	0
D02	1077	A	16	0	10	0	0	0
D02	1095	T	16	0	1	0	0	0
D02	1124	A	16	0	0	16	0	0
D02	1125	C	16	0	0	0	16	0
D02	1126	A	16	0	1	0	0	0
D02	1128	A	16	0	6	0	0	0
D02	1135	A	16	0	0	6	0	0
D02	1139	A	16	0	2	0	0	0
D02	1173	G	16	14	1	0	0	0
D02	1175	A	16	0	0	16	0	0
D02	1218	C	16	0	0	0	1	0
D02	1252	T	16	0	0	16	0	0
D02	1254	T	16	0	0	1	0	0
D02	1278	A	16	0	0	1	0	0
D02	1287	T	16	0	16	0	0	0
D02	1306	A	16	0	16	0	0	0
D02	1320	C	16	16	0	0	0	0
D02	1321	C	16	2	0	0	0	0
D02	1337	A	16	0	1	0	0	0
D02	1350	T	16	0	0	1	0	0
D02	1362	C	16	0	0	0	16	0
D02	1365	A	16	0	1	0	0	0
D02	1375	T	16	0	1	0	0	0
D02	1381	C	16	0	0	0	1	0
D02	1417	T	16	0	1	0	0	0
D02	1425	T	16	0	16	0	0	0
D02	1432	C	16	0	0	0	1	0
D02	1437	A	16	0	0	16	0	0
D02	1450	G	16	1	0	0	0	0
D02	1480	A	16	0	0	1	0	0
D02	1485	T	16	0	16	0	0	0
D02	1497	C	16	0	0	0	16	0
D02	1499	G	16	11	0	0	0	0
D02	1508	T	16	15	0	0	0	0
D02	1511	G	16	1	0	0	0	0
D02	1544	T	16	16	0	0	0	0
D02	1553	T	16	0	1	0	0	0
D02	1575	C	16	0	0	0	1	0
D02	1583	A	16	0	0	1	0	0
D02	1632	A	16	0	16	0	0	0
D02	1635	G	16	16	0	0	0	0
D02	1637	C	16	15	0	1	0	0
D02	1647	G	16	1	0	0	0	0
D02	1652	G	16	3	0	0	0	0
D02	1655	T	16	0	2	0	0	0
D02	1657	A	16	0	0	1	0	0
D02	1691	C	16	0	0	0	1	0
D02	1742	G	16	14	0	0	2	0
D02	1747	A	16	0	5	0	0	0
D02	1748	G	16	0	0	0	9	0
D02	1751	G	16	0	0	0	8	0
D02	1752	A	16	0	0	1	1	0
D02	1753	T	16	5	11	0	0	0
D02	1762	A	16	0	0	0	16	0
D02	1763	G	16	1	0	0	0	0
D02	1766	C	16	0	0	0	10	0
D02	1768	T	16	11	0	0	0	0
D02	1769	C	16	0	0	0	16	0
D02	1773	C	16	0	0	0	8	0
D02	1820	A	16	0	16	0	0	0
D02	1846	A	16	0	0	0	1	0
D02	1853	T	16	0	1	0	0	0
D02	1862	G	16	0	0	0	1	0
D02	1896	G	16	11	0	0	0	0
D02	1899	G	16	1	0	0	0	0
D02	1917	A	16	0	0	0	1	0
D02	1944	T	16	0	1	0	0	0
D02	1961	T	16	11	0	0	0	0
D02	1962	C	16	1	0	0	0	0
D02	1966	T	16	0	1	0	0	0
D02	1975	G	16	8	0	0	0	0
D02	1983	G	16	11	0	0	0	0
D02	1984	G	16	16	0	0	0	0
D02	1987	C	16	0	0	0	1	0
D02	1990	T	16	0	9	0	0	0
D02	1991	C	16	10	0	0	1	0
D02	1999	C	16	2	0	0	0	0
D02	2003	T	16	11	0	0	0	0
D02	2011	C	16	0	0	16	0	0
D02	2035	A	16	0	0	16	0	0
D02	2037	A	16	0	0	1	0	0
D02	2038	A	16	0	0	1	0	0
D02	2044	T	16	0	9	0	0	0
D02	2053	C	16	0	0	0	7	0
D02	2074	T	16	16	0	0	0	0
D02	2075	G	16	16	0	0	0	0
D02	2083	T	16	0	5	0	0	0

D02	2086	G	16	0	0	0	11	0
D02	2089	T	16	0	0	16	0	0
D02	2091	A	16	0	0	1	0	0
D02	2097	T	16	10	0	1	0	0
D02	2098	G	16	0	0	0	11	0
D02	2109	C	16	1	0	0	0	0
D02	2120	A	16	0	0	5	0	0
D02	2126	T	16	0	1	0	0	0
D02	2131	A	16	0	11	0	0	0
D02	2145	G	16	11	0	0	0	0
D02	2150	T	16	5	0	0	0	0
D02	2160	G	16	1	0	0	0	0
D02	2176	T	16	0	8	0	0	0
D02	2191	C	16	0	0	0	16	0
D02	2198	C	16	5	0	0	0	0
D02	2203	T	16	0	0	16	0	0
D02	2219	T	16	0	1	0	0	0
D02	2239	A	16	0	16	0	0	0
D02	2245	C	16	0	0	0	16	0
D02	2260	G	16	16	0	0	0	0
D02	2266	C	16	0	0	0	16	0
D02	2271	T	16	0	1	0	0	0
D02	2290	C	16	5	0	0	0	0
D02	2304	C	16	11	0	0	0	0
D02	2345	G	16	11	0	0	0	0
D02	2351	C	16	0	0	10	0	0
D02	2352	G	16	1	0	0	0	0
D02	2364	C	16	0	0	11	0	0
D02	2369	A	16	0	0	11	0	0
D02	2376	G	16	1	0	0	0	0
D02	2401	A	16	0	0	16	0	0
D02	2456	T	16	0	16	0	0	0
D02	2489	G	16	0	0	0	1	0
D02	2538	T	16	0	1	0	0	0
D02	2567	C	16	0	0	0	5	0
D02	2590	T	16	0	1	0	0	0
D02	2618	A	16	0	0	16	0	0
D02	2621	A	16	0	0	16	0	0
D02	2636	C	16	0	0	0	16	0
D02	2642	T	16	0	1	0	0	0
D02	2659	A	16	0	8	0	0	0
D02	2660	C	16	3	0	2	0	0
D02	2663	T	16	0	0	1	0	0
D02	2675	A	16	0	0	16	0	0
D02	2681	G	16	16	0	0	0	0
D02	2711	G	16	16	0	0	0	0
D02	2720	T	16	0	10	0	0	0
D02	2810	C	16	0	0	0	16	0
D02	2853	A	16	0	0	1	0	0
D02	2854	G	16	8	0	0	1	0
D02	2865	C	16	2	0	0	0	0
D02	2875	A	16	0	0	1	0	0
D02	2946	A	16	0	0	5	0	0
D02	2973	G	16	0	0	0	5	0
D02	3040	T	16	0	5	0	0	0
D02	3045	G	16	0	0	0	5	0
D02	3051	T	16	0	5	0	0	0
D02	3057	T	16	5	0	0	0	0
D02	3059	A	16	0	0	1	0	0
D02	3087	G	16	5	0	0	0	0
D02	3088	G	16	0	0	0	5	0
D02	3099	T	16	5	0	0	0	0
D02	3144	G	16	1	0	0	0	0
D02	3154	A	16	0	0	4	0	0
D02	3166	C	16	0	0	0	1	0
D02	3183	A	16	0	0	11	0	0
D02	3196	C	16	0	0	0	1	0
D02	3198	T	16	11	0	0	0	0
D02	3209	A	16	0	0	11	0	0

Table S6 Amino acid variations for A epitope (aa124–aa147).

Genotype C				Genotype I	
**Experimental group**	**Number**	**Control group**	**Number**	**Experimental group**	**Number**
C124S	1	I126S	13	Y134N	11
T125M	3	I126G	2	P135L	8
I126T	24	Q129R	2	G145R	8
I126V	3	M133T	1	N146T	1
G130N	9	K141E	1		
G130R	5				
G130E	1				
T131I	1				
T131N	1				
M133T	5				
F134Y	14				
D144V	6				
D144A	3				

Number of substitutions per 100 amino acid

	**Experimental group**	**Control group**	**p**
**a' determinant (aa 124-147)**	4.76	0.91	0.0001

Table S7 Number of variation at Core Promoter and pre-Core region (nt1742-1900) in present study.

mutations	Experimental group (91)		Control group (87)		*p* value Chi-square test
	wild type	mutant type	wild type	mutant type	
A1762T	13	78	63	24	1.52064E-14
G1764A	13	78	58	29	2.92985E-12
T1753C	51	40	87	0	7.71992E-12
A1755C	76	15	87	0	0.00022653
G1742A	77	14	87	0	0.000410948
G1896A	77	14	87	0	0.000410948
T1802C	78	13	87	0	0.000742127
G1899A	78	13	87	0	0.000742127
T1768A	80	11	87	0	0.002392226
A1846T	77	14	85	2	0.005285676
C1773T	83	8	87	0	0.013586221
A1775G	76	15	82	5	0.042368534
T1861C	88	3	87	0	0.260341026
C1766T	81	10	72	15	0.324954096
C1799G	78	13	75	12	1

Table S8 Amino acid variations for genetype C HBV genome.

Genes	Position	Mutations	Experimental group (62 clones)		Control group (59 clones)		*p* value Chi-square test	Epitopes
			wild type	mutant type	wild type	mutant		
HBcAg	13	V13L	48	14	59	0	0	HLA I/II T,B
HBcAg	21	S21A	62	0	43	16	0	HLA I/II T,B
HBcAg	24	F24S	62	0	57	2	0.236	HLA I/II T,B
HBcAg	25	V25I	0	62	59	0	0	HLA I/II T,B
HBcAg	35	S35A	48	14	59	0	0	HLA I/II T,B
HBcAg	58	A58D	48	14	59	0	0	HLA II T,B
HBcAg	60	V60L	0	62	0	59	1	HLA I/II T,B
HBcAg	83	E83D	58	4	57	2	0.68	HLA I/II T,B
HBcAg	87	S87G/R	56	6	57	2	0.274	HLA I/II T,B
HBcAg	94	I94L	62	0	57	2	0.236	HLA I/II T,B
HBcAg	96	K96N	60	2	59	0	0.496	HLA I/II T,B
HBcAg	97	I97L	48	14	59	0	0	HLA I/II T,B
HBcAg	100	L100I	50	12	59	0	0	HLA I/II T,B
HBcAg	101	l101W	61	1	58	1	1	HLA I/II T,B
HBcAg	112	R112G	62	0	56	3	0.113	HLA I/II T,B
HBcAg	115	V115A	62	0	56	3	0.113	HLA I/II T,B
HBcAg	119	L119S	62	0	56	3	0.113	HLA I / II T
HBcAg	120	V120A	62	0	56	3	0.113	HLA I/II T,B
HBcAg	130	P130S/T	48	14	29	30	0.001	HLA I/II T,B
HBcAg	135	P135Q	61	1	58	1	1	HLA I/II T,B
HBcAg	148	V148I	48	14	59	0	0	HLA I/II T,B
HBcAg	152	R152Q	58	4	59	0	0.119	HLA I/II T,B
HBcAg	153	G153C	62	0	57	2	0.236	HLA I/II T,B
HBcAg	155	S155T	60	2	59	0	0.496	HLA II T,B
HBcAg	162	S162L	62	0	56	3	0.113	HLA I/II T,B
HBcAg	170	S170P	62	0	56	3	0.113	HLA II T,B
HBcAg	177	Q177R	60	2	54	5	0.265	HLA II T,B
HBcAg	180	E180G	62	0	44	15	0	HLA II T,B
HBxAg	4	R4G	56	6	59	0	0.028	B
HBxAg	5	L5V/P	16	46	29	30	0.009	B
HBxAg	21	V21F	58	4	59	0	0.119	B
HBxAg	29	S29P	0	62	0	59	1	B
HBxAg	30	V30I	62	0	41	18	0	B
HBxAg	32	S32R	58	4	59	0	0.119	B
HBxAg	35	G35R	54	8	59	0	0.006	B
HBxAg	36	G36S/A/T	0	62	12	47	0	B
HBxAg	38	P38S/L	60	2	59	0	0.496	B
HBxAg	42	L42S	0	62	12	47	0	B
HBxAg	43	S43P	55	7	44	15	0.059	B
HBxAg	47	A47T	54	8	44	15	0.105	B
HBxAg	48	N48D	0	62	0	59	1	B
HBxAg	59	P59S	62	0	57	2	0.236	HLA I T,B
HBxAg	61	C61R/Y	62	0	53	6	0.012	HLA II T,B
HBxAg	64	S64T	62	0	47	12	0	HLA II T,B
HBxAg	79	M79I	58	4	59	0	0.119	B
HBxAg	86	R86H/L/S	15	47	41	18	0	B
HBxAg	94	Y94H	0	62	0	59	1	HLA I T,B
HBxAg	95	K95N	62	0	45	14	0	HLA I/II T,B
HBxAg	96	R96K/G	60	2	59	0	0.496	HLA I/II T,B
HBxAg	101	S101P/A	52	10	44	15	0.263	HLA I/II T,B
HBxAg	112	F112S	58	4	59	0	0.119	HLA II T,B
HBxAg	116	V116L	29	33	44	15	0.003	HLA I/II T,B
HBxAg	123	L123S	62	0	41	18	0	HLA I/II T,B
HBxAg	127	I127T	33	29	59	0	0	HLA II T,B
HBxAg	130	K130M	13	49	41	18	0	HLA II T,B
HBxAg	131	I131V/T	49	13	35	24	0.029	HLA II T,B
HBxAg	132	Y132F	0	62	17	42	0	HLA II T,B
LHBsAg	1	M1T	61	1	58	1	1	
LHBsAg	10	K10Q	4	58	12	47	0.032	HLA II T
LHBsAg	14	T14S	62	0	54	5	0.025	HLA II T
LHBsAg	27	D27G	48	14	41	18	0.41	HLA I T,B
LHBsAg	35	G35R	62	0	56	3	0.113	HLA I T,B
LHBsAg	41	P41S	62	0	47	12	0	B
LHBsAg	50	D50G	60	2	59	0	0.496	
LHBsAg	51	H51Q	47	15	41	18	0.541	
LHBsAg	54	A54E	15	47	30	29	0.003	
LHBsAg	55	N55D/E	54	8	59	0	0.006	
LHBsAg	60	A60V	37	25	41	18	0.342	B

LHBsAg	62	A62S	47	15	41	18	0.541	B
LHBsAg	67	F67L	60	2	59	0	0.496	
LHBsAg	73	S73G	20	42	30	29	0.044	B
LHBsAg	77	W77S	52	10	59	0	0.001	HLA I T,B
LHBsAg	88	V88M/A	57	5	59	0	0.058	B
LHBsAg	90	A90V	49	13	44	15	0.667	B
LHBsAg	98	N98T	60	2	59	0	0.496	
LHBsAg	102	G102R	60	2	59	0	0.496	
LHBsAg	105	P105S	57	5	59	0	0.058	B
LHBsAg	120	M120T/V	48	14	59	0	0	HLA I/II T,B
LHBsAg	125	T125S	47	15	41	18	0.541	HLA I/II T,B
LHBsAg	128	H128L	48	14	59	0	0	HLA I/II T,B
LHBsAg	130	A130T	49	13	59	0	0	HLA I/II T,B
LHBsAg	132	L132Q	48	14	59	0	0	HLA I/II T,B
LHBsAg	133	D133G	62	0	57	2	0.236	HLA I/II T,B
LHBsAg	141	F141C	60	2	59	0	0.496	HLA II T,B
LHBsAg	152	N152S	59	3	58	1	0.619	HLA II T,B
LHBsAg	158	A158V	55	7	59	0	0.013	HLA II T,B
LHBsAg	161	I161V	62	0	41	18	0	HLA II T,B
LHBsAg	164	I164V	60	2	59	0	0.496	B
LHBsAg	165	F165S	48	14	59	0	0	
LHBsAg	168	T168I	49	13	59	0	0	
LHBsAg	173	A173P	14	48	0	59	0	HLA I T
LHBsAg	173	A173L	48	14	59	0	0	HLA I T
LHBsAg	174	N174S	62	0	55	4	0.054	HLA I T
LHBsAg	177	N177S	16	46	41	18	0	HLA I T/HLA II T
LHBsAg	178	T178I	48	14	59	0	0	HLA I T/HLA II T
LHBsAg	182	F182L	60	2	59	0	0.496	HLA I/II T,B
LHBsAg	184	G184R/K/T	46	16	59	0	0	HLA I/II T,B
LHBsAg	188	V188A	47	15	59	0	0	HLA I/II T,B
LHBsAg	194	F194S	60	2	59	0	0.496	HLA I T,B
LHBsAg	195	L195W	58	4	59	0	0.119	HLA I T,B
LHBsAg	195	L195S	60	2	59	0	0.496	HLA I T,B
LHBsAg	198	R198K	60	2	59	0	0.496	HLA I T,B
LHBsAg	208	S208P	62	0	57	2	0.236	HLA I T,B
LHBsAg	219	A219V/T	46	16	59	0	0	B
LHBsAg	220	P220S/T	46	16	59	0	0	B
LHBsAg	221	T221V/A	48	14	56	3	0.008	B
LHBsAg	223	P223R	48	14	59	0	0	B
LHBsAg	227	L227S	13	49	30	29	0.001	B
LHBsAg	235	S235L	57	5	53	6	0.759	B
LHBsAg	236	P236L	52	10	58	1	0.009	B
LHBsAg	237	T237I	60	2	59	0	0.496	B
LHBsAg	242	T242I	34	28	12	47	0	B
LHBsAg	250	C250Y	55	7	59	0	0.013	HLA I T
LHBsAg	254	F254L	59	3	59	0	0.244	HLA I T
LHBsAg	261	L261P	61	1	58	1	1	HLA I T
LHBsAg	269	L269W	60	2	59	0	0.496	HLA I T,B
LHBsAg	272	L272V	52	10	59	0	0.001	HLA I T,B
LHBsAg	273	D273G	61	1	56	3	0.356	HLA I T,B
LHBsAg	283	L283P	62	0	55	4	0.054	B
LHBsAg	284	L284I	53	9	59	0	0.003	B
LHBsAg	287	T287S/N/P	48	14	56	3	0.003	B
LHBsAg	296	K296R	48	14	59	0	0	HLA I T,B
LHBsAg	299	T299M	59	3	59	0	0.244	HLA I/II T,B
LHBsAg	300	I300V/T/G/S	34	28	44	15	0.036	HLA I/II T,B
LHBsAg	301	G301R/N	48	14	59	0	0	HLA I/II T,B
LHBsAg	303	Q303R	62	0	57	2	0.236	HLA I/II T,B
LHBsAg	307	M307T	57	5	58	1	0.208	HLA II T,B
LHBsAg	308	F308Y	48	14	59	0	0	HLA II T,B
LHBsAg	318	D318A/V	53	9	59	0	0.003	HLA I/II T,B
LHBsAg	323	C323R	62	0	56	3	0.113	
LHBsAg	333	A333G	48	14	59	0	0	HLA I T
LHBsAg	334	R334K	48	14	59	0	0	HLA I T
LHBsAg	336	L336Q	62	0	44	15	0	HLA I T
LHBsAg	342	V342A	47	15	59	0	0	HLA I T
LHBsAg	349	L349S	62	0	47	12	0	HLA I T
LHBsAg	351	V351A	62	0	56	3	0.113	HLA I T
LHBsAg	359	G359R	60	2	59	0	0.496	HLA I T
LHBsAg	368	A368V	0	62	13	46	0	
LHBsAg	371	M371T	60	2	59	0	0.496	HLA I T
LHBsAg	377	P377Q	58	4	59	0	0.119	HLA I T
LHBsAg	378	S378R	48	14	59	0	0	HLA I T

LHBsAg	381	N381S	48	14	59	0	0	HLA I T
LHBsAg	382	I382T	47	15	59	0	0	HLA I T
LHBsAg	384	S384N	47	15	41	18	0.541	HLA I T
LHBsAg	390	L390S	58	4	59	0	0.119	HLA I T
LHBsAg	398	V398A	58	4	59	0	0.119	
RT	7	S7A/T	0	62	0	59	1	
RT	9	H9Y	49	13	42	17	0.4	HLA I T
RT	13	N13H	34	28	41	18	0.134	
RT	16	I16T	60	2	59	0	0.496	
RT	18	R18K/N	46	16	59	0	0	
RT	19	T19A	60	2	59	0	0.496	
RT	23	V23A	62	0	57	2	0.236	
RT	38	T38A	48	14	47	12	0.827	HLA I T
RT	42	L42P	62	0	57	2	0.236	
RT	50	S50P	60	2	59	0	0.496	HLA I T
RT	53	S53N	48	14	59	0	0	HLA I T
RT	54	T54I/Y	46	16	59	0	0	
RT	55	H55R	48	14	56	3	0.008	
RT	88	F88S	59	3	59	0	0.244	
RT	91	I91L	35	27	41	18	0.188	
RT	106	S106C	51	11	59	0	0.001	HLA I T
RT	109	P109S	48	14	41	18	0.41	HLA I T
RT	118	T118N	53	9	59	0	0.003	
RT	121	N121K/I/T	48	14	56	3	0.008	
RT	122	F122I	14	48	13	46	0.558	
RT	123	N123D	48	14	59	0	0	
RT	124	Y124H	48	14	59	0	0	
RT	126	H126Y	62	0	47	12	0	
RT	128	T128A	62	0	41	18	0	
RT	129	M129L	49	13	59	0	0	
RT	129	M129V	57	5	59	0	0.058	
RT	131	D131N	48	14	59	0	0	
RT	134	D134G	60	2	57	2	1	
RT	134	D134E	61	1	46	13	0	
RT	135	T135S	0	62	12	47	0	
RT	138	R138K	48	14	59	0	0	
RT	139	N139H	53	9	59	0	0.003	
RT	145	L145M	60	2	59	0	0.496	
RT	157	L157S	62	0	56	3	0.113	HLA I T
RT	163	I163V	42	20	59	0	0	HLA I T
RT	170	P170S	59	3	59	0	0.244	
RT	193	R193K	60	2	59	0	0.496	
RT	213	S213T	48	14	59	0	0	HLA I T
RT	223	S223A	33	29	59	0	0	
RT	224	I224V	46	16	59	0	0	
RT	253	V253R/I	48	14	56	3	0.008	
RT	263	E263D	48	14	59	0	0	
RT	267	L267H/Q	14	48	27	32	0.008	
RT	269	I269L	14	48	36	23	0	
RT	271	P271Q	0	62	0	59	1	
RT	278	V278I	48	14	41	18	0.41	
RT	280	S280R	0	62	0	59	1	
RT	282	I282V	48	14	59	0	0	
RT	283	D283G	56	6	59	0	0.028	
RT	287	C287R	62	0	56	3	0.113	
RT	289	R289K	62	0	57	2	0.236	
RT	292	C292G	56	6	0	59	0	
RT	297	A297V	56	6	59	0	0.028	HLA I T

Table S9 Deletion , insertion and stop codon mutations of HBV quasispecies observed in the present study.

**Clinical spectrum**	**No. of Del , Ins or Stop codonmutations patients /total no.of patients**	**Patient ID (genotype)**	**No. of Del , Ins or Stop codon mutations clones/total no. of clones**	**Del/Insnt patterns(nt positions in fulllength HBV genome)**	**Del/Ins aa patterns (aa positions of related region)**
Experimental group	6/6	D10(B)	1/13	nt1627	Del and frameshift HBxAg at aa85 with stop codon
			1/13	nt3165-3189	Del and frameshift LHBsAg at aa98-106 with stop codon,Del and frameshift Pol at aa278-286 with stop codon
			2/13	nt1896G-A	PreC/C W28*
		D15(C)	4/14	nt2971-2988	Del LHBsAg at aa42-47, Pol at aa223-228
			4/14	nt3141-3165	Del LHBsAg aa66-112,Pol at aa246-292
			4/14	nt41-43	LHBsAg R137*
			1/14	nt1376G-A	Pol W762*
			1/14	nt1374	Del HBxAg start codon,
			4/14	nt1610	Pol W840*
			1/14	nt1099-1584	Del Pol at aa670-831
		D02(I)	11/16	nt2863-3138	Del LHBsAg aa6-97, Pol aa186-277
			8/16	nt503-526	Del LHBsAg aa291-298, Pol aa471-478
			1/16	nt670G-A	LHBsAg W346*
			1/16	nt700G-A	LHBsAg W356*
			8/16	nt750G-A	LHBsAg W373*
			11/16	nt1896G-A	PreC/C W28*
		D23(C)	1/20	nt1896G-A	PreC/C W28*
		D24(C)	1/13	nt3077	Del and frameshift LHBsAg at aa77 with stop codon, Del and frameshift Pol at aa257 with stop codon
		D07(C)	2/15	nt3022G-T	LHBsAg G59*
			1/15	nt79	Del and frameshift LHBsAg at aa149 with stop codon,Del and frameshift Pol at aa329 with stop codon
Control group	5/6	CFQ(C)	1/14	nt2859G-A	LHBsAg W4*
			1/14	nt3208C-T	LHBsAg Q121*
			1/14	nt1421	Del and frameshift Pol at aa776 with stop codon, Del and frameshift HBxAg at aa16 with stop codon
			1/14	nt2109	Del and frameshift PreC/C at aa 98 with stop codon
			2/14	nt2132-2217	Del PreC/C aa107-134
			1/14	nt2102-2217	Del PreC/C aa93-140
			5/14	nt1744-1763	Del and frameshift HBxAg at aa 124-130 with stop codon
			1/14	nt1755-1776	Del and frameshift HBxAg at aa 128-134 with stop codon
		KXY(C)	7/18	nt2848-2862	Del LHBsAg start codon, Del Pol aa181-186
			1/18	nt121	Del and frameshift LHBsAg at aa163 with stop codon,Del and frameshift Pol at aa343 with stop codon
			2/18	nt2197-2212	Del and frameshift PreC/C at aa128-133 with stop codon
			3/18	nt1647	Ins HBxAg aa93-100
			13/18	nt1755-1776	Del and frameshift HBxAg at aa 128-134 with stop codon
			3/18	nt1751-1769	Del and frameshift HBxAg at aa 126-132 with stop codon
		LYL(B)	2/12	nt121	Del and frameshift LHBsAg at aa163 with stop codon,Del and frameshift Pol at aa343 with stop codon
		XDH(C)	1/12	nt2003	Del and frameshift PreC/C at aa64 with stop codon
			2/16	nt2848-2865	Del LHBsAg start codon with stop codon,Del Pol aa181-186
			1/16	nt302G-A	Pol W404*

		LMX(I)	1/16	nt1376G-A	Pol W762*
			1/16	nt1988-2043	Del and frameshift PreC/C at aa59-77 with stop codon
			1/16	nt2081-2241	Del and frameshift PreC/C at aa90-143 with stop codon
			1/16	nt2087-2230	Del PreC/C aa92-139
			9/16	nt2134-2220	Del PreC/C aa108-136
